# Integrating continental mainland and islands in temperate East Asia: liverworts and hornworts of the Korean Peninsula

**DOI:** 10.3897/phytokeys.176.56874

**Published:** 2021-04-20

**Authors:** Seung Se Choi, Vadim Bakalin, Seung Jin Park

**Affiliations:** 1 Team of National Ecosystem Survey, National Institute of Ecology, Seocheon, 33657, Republic of Korea National Institute of Ecology Seocheon Republic of Korea; 2 Botanical Garden-Institute, Makovskogo Street, 142, Vladivostok, 690024, Russia Botanical Garden-Institute Vladivostok Russia; 3 Department of Life Science, Jeonbuk National University, Jeonju, 54896, Republic of Korea Jeonbuk National University Jeonju Republic of Korea

**Keywords:** Anthocerotae, distribution, Hepaticae, Korean peninsula, phytogeography

## Abstract

The liverwort and hornwort flora of the Korean Peninsula possesses some unique traits arising from the geographic position of the Peninsula, where the mainland flora meets insular flora. This flora is still not exhaustively studied, due not only to political reasons, but also because much less attention has been paid than to adjacent lands by hepaticologists. A checklist presented is based on a study of ca. 15,500 specimens collected by the authors and a review of relevant literature. This study provides the checklist of liverworts and hornworts known from Korea and the geographical distribution of each species within the peninsula. The liverworts and hornworts in Korean flora include 346 taxa (326 species, 16 subspecies and four varieties) in 112 genera and 50 families. Since 2007, 75 taxa of liverworts and four taxa of hornworts are reported as new to the Korean Peninsula, with a number of the new records arising following application of new taxonomic concepts that have become apparent over the last few decades. While compiling the checklist, 42 species, previously reported to Korea, are excluded from the Korean liverwort flora.

## Introduction

The Korean Peninsula is situated on the easternmost temperate edge of the Pacific Asia mainland. It belongs to the East Asiatic floristic region and it has close floristic relationships with Manchuria (in the broad sense of this term) and Japan (Takhtajan 1986). Korea shows a remarkable variation in climatic conditions regarding temperature and precipitation. In addition, the complex mountain system that covers nearly 70% of the total area and ca. 3,400 islands along the west and south coast create a great diversity of habitats throughout the area. The flora, therefore, shows high diversity, caused by the range of communities from warm temperate, merging subtropical in the southern part to cold temperate and alpine types in the northern and high mountain regions.

The first reports of liverworts for the Korean Peninsula were those by Stephani (1909) – an incidental result obtained in the course of study of Japanese flora by U. Faurie (Bakalin and Katagiri 2014). Since then, Japanese botanists, starting with Nakai (1918), have studied liverworts and hornworts in Korea. Hong (1960a) and Choe (1975) were the first Koreans who revised liverworts and hornworts in Korea (see Taxonomic History below). Since the 1990s, studies on liverworts and hornworts in Korea were generally accomplished by reviews of literature data (Hong 1997; Yamada and Choe 1997; Park and Choi 2007). In some cases, as authors simply provided the lists of taxa without any references even to published papers, the origin of some reports could not be determined. Therefore, despite the existence of many publications dealing with Korean liverworts, the distribution of taxa within the Peninsula and even the certain presence of some species remain questionable. This is in stark contrast to the obvious progress in knowledge of vascular plants over the last several decades as exemplified by several monographic treatments and even some illustrated guidebooks (Lee 1980; Lee 1996; Lee 2006; Park 2007). Previously, the illustrated Flora and Fauna of Korea 24 and Korean Spore Plant 8 (Choe 1980; Kim and Hwang 1991) attempted to fill this gap, but actually they provided only poor data, based on limited amount of collected material and could hardly be regarded as sufficient and satisfactory in the current time. Hence, we attempt a full revision of all available collections, including those newly collected within the past 12 years and literature sources. We provide original information on the distribution of taxa within the Peninsula and arrange them in accordance with the currently accepted taxonomy, supported by recent advances in molecular-genetic research. We stress that the liverwort and hornwort flora, due to unique geological, environmental, and geographical factors, is far from a simple ‘incomplete copy’ of Japanese or North-East China floras (Choi et al. 2010, 2011, 2012a, b, c, 2020a, b; Bakalin et al. 2020a). Moreover, Korean flora possesses many unique geological, environmental, and geographical factors caused by the Peninsula being situated between continental mainland and islands on the migration route of inter-exchanges within various regions in East Asia and even within East and Northeast Asia in the broader context.

## Historical background

The history of knowledge about liverworts and hornworts in Korea can be divided into four stages, based on botanists involved at the different time periods (Table 1). The first stage (1900–1930) is the starting stage, the second stage (1930–1960) includes studies conducted mainly by Japanese botanists, the third stage (1960–2007) includes studies by the first Korean professional bryologists Drs. W.H. Hong, D.-M. Choe and Y.H Kim and the last stage (2008–present) includes studies by various botanists in both North and South Korea.

**Table 1.** Taxonomical history of four phases, based on representative botanists for the Korean Peninsula.

Phases	Year	Main authors	Main collectors
First	1900–1930	Stephani F	Faurie U, Taquet E
Second	1930–1960	Horikawa Y	Horikawa Y
		Hattori S	Hattori S
Third	1960–2007	Hong WS	Hong WS
		Choe DM	Choe DM
Fourth	2008–present	Kim YH, Hwang HJ	Kim YH, Hwang HJ
		Choi SS, Bakalin VA	Choi SS, Bakalin VA

### First stage (1900–1930)

Faurie (1846–1915; referenced in Bakalin and Katagiri 2014) was the first who collected liverworts on the Korean Peninsula. In collaboration with Taquet (1873–1952; referenced in Oh 1976), he sampled diverse hepatics and mosses while amassing a large collection of vascular plants. The liverwort collections were studied by Stephani (1909, 1910a, b, c, 1911, 1916, 1917a, b, c, 1922, 1924) and listed in his well-known Species Hepaticarum. Eight species [*Frullania fusco-virens*, *Frullania koreana* (= *Frullania hamatiloba*), *Anthoceros koreanus* (= *Pheoceros carolinianus*), *Plagiochasma koreanum* (= *Plagiochasma japonicum*), *Solenostoma koreanum* (=*Protosolenostoma fusiforme*), *Jungermannia decurrens* (= *Solenostoma faurieanum*), *Lepidozia coreana* (= *Lepidozia subtransversa*), and *Mastigobryum coreanum* (= *Bazzania tridens*)] from the collection were described by Stephani as new to science and three species [*Frullania muscicola*, *Ptilidium sacculatum* (= *Trichocoleopsis sacculata*), and *Madotheca tosana* (= Porella acutifolia subsp. tosana)] were recorded as new to the Korean Peninsula. Nakai (1918) reported one hepatic [*Scapania dentata* (= *Scapania undulata*)] and 31 mosses.

### Second stage (1930–1960)

During this time period, Korean hepatics have been studied mostly by Japanese botanists, including Horikawa (1932, 1934a, b, c, d, 1935a, b, c, 1936a, b, c, 1951a, b, 1955), Uno and Takahashi (1940), Hattori (1941, 1943a, b, 1944a, b, 1948, 1950, 1967, 1970, 1975a), Hattori and Mizutani (1958), Hattori et al. (1962a, b), Ando (1955, 1960), Amakawa (1959, 1960), Inoue (1958a, b, 1959a, b, 1962), Yamada (1979, 1989). Besides, Reimers (1931) studied materials collected by Klautke and listed in his well-known paper, “Ein Beitrag zur Moosflora von Korea”. Nine species [*Plagiochila delavayi*, *Jamesoniella autumnalis* (= *Syzygiella autumnalis*), *Mastigobryum bidentulum* (= *Bazzania bidentula*), *Blepharostoma trichophyllum*, *Radula japonica*, *Frullania jackii* (= *Frullania davurica*), *Frullania clavellata* (= *Frullania appendiculata*), *Lejeunea cavifolia* and *Lejeunea compacta*] were recorded as new to the Korean Peninsula and one species [*Ptilidium sacculatum* (= *Trichocoleopsis sacculata*)] was additionally recorded. Horikawa conducted numerous bryofloristic research studies in Asia. He reported 67 species as new to the Korean Peninsula and one species (*Fimbriaria koreana* (= *Asterella leptophylla*)) as new to science (Horikawa 1932, 1934a, b, c, d, 1935a, b, c, 1936a, b, c, 1951a, b, 1955). Uno and Takahashi (1940) listed four species *Brachiolejeunea sandvicensis* (= *Acrolejeunea sandvicensis*), *Frullania fauriana*, Frullania moniliata subsp. obscura (= *Frullania appendiculata*) and *Madotheca setigera* (= Porella caespitans var. cordifolia) from Mt Jiri. Hattori (1941, 1943a, b, 1948, 1950) recorded eight species for Korean Peninsula: *Bazzania coreana* (= *Bazzania tridens*), *Jungermannia cordifolia* (= *Jungermannia exsertifolia*), *Targionia hypophylla*, *Plagiochila ovalifolia*, Frullania nepalensis var. nishiyamensis (= *Frullania nepalensis*), *Riccia fluitans* and *Riccia glauca*. He also studied specimens from Mt. Jiri and Mt. Halla collected by Hong and described a species new to science (*Metacalypogeia quelpaertensis* (= *Eocalypogeia quelpaertensis*)) (Hattori et al. 1962a, 1962b). Ando (1955, 1960) recorded three species (Porella vernicosa subsp. vernicosa, P. vernicosa subsp. fauriei (= *P. fauriei*) and P. vernicosa subsp. gracillima (= *P. gracillima*)) in his papers on *Porella* complex. Amakawa (1960) reported one species (*Nardia sieboldii* (= *Nardia assamica*)) from Korea. Inoue (1962) described *Plagiochila quelpaertensis* (= *P. ovalifolia*) as new to science collected by Hong.

### Third stage (1960–2007)

The first Korean botanist who studied and published on liverworts and hornworts was Won Shic Hong (1919–2014; 1960a, b, c, 1962a, b, c, d, 1966, 1997, 2003). He reported 149 taxa in 53 genera and 24 families, based on a study of ca. 5,000 herbarium specimens (Hong 1962a). Additionally, Hong (1997) markedly expanded his list that counts 259 taxa belonging to 76 genera in 37 families on the basis of personal collections and literature records, including those for North Korea (Gao and Cao 1983; Gao and Chang 1983a, b; Koponen et al. 1983; Huneck et al. 1987). Hong also provided identification keys to 263 known or expected taxa in the Korean Peninsula (Hong 2003). Hong (1962a) was the first who compiled the manual for the Korean liverworts, published in English. Hong’s follower, Du Moon Choe (1925–2014; 1975, 1979, 1980, 1983), conducted bryofloristic research studies in South Korea. He published a manual of Korean bryophytes in the Korean language, which includes 201 liverwort taxa in 66 genera (Choe 1980). Later, in collaboration with the Japanese hepaticologist Kohsaku Yamada, 236 taxa of Hepaticae were assembled in a special checklist providing information on distributions of species within administrative provinces of both countries on the Korean Peninsula (Choe and Yamada 1979, 1997, 1998, 2000; Yamada and Choe 1997). In North Korea, Gao and Cao (1983) compiled the list of bryophytes of Mt. Baekdu, including 85 liverworts. Kim and Hwang (1991) provided description and identification keys to 207 known taxa on the Korean Peninsula. Song and Yamada (2006, 2009a, b) examined the hepatic flora of Jeju Island, Mt. Jiri and Mt. Gaya. Kwang Woo Park and Kyeong Choi (2007) published a list of bryophytes for the Korean Peninsula, based on literature data. The list includes 281 taxa belonging in 81 genera.

### Fourth stage (2008-present)

Our own studies on Korean liverworts and hornworts started in 2008 and revealed many new species, including those new to science. As was found previously, the Korean liverwort flora was still poorly understood and peculiar traits of flora of this Peninsula were simply overlooked. Bakalin et al. (2009, 2020a) described *Tritomaria koreana* and *Solenostoma jirisanense* from Mt. Jiri as new to science. Choi et al. (2010, 2011, 2012a, b, c, 2020a, b) continuously reported unrecorded species from the Korean hepatic flora. Concurrently with our research, Lee et al. (2011) published the National List of Species of Korea, including 277 liverworts and eight hornworts, based on literature sources. After Hong (1962a), the number of genera and species of liverworts and hornworts in Korea, based on literature, has been listed in Table 2.

Taking into account that checklists commonly become outdated every 10 years and many new additions have been obtained within the last stage of Korean bryophyte flora recognition, the present checklist marks the new ‘frontier’ in the knowledge of Korean bryophytes that, at the same time, is the starting point for new achievements in this field.

**Table 2.** The number of genera and species of liverworts and hornworts in Korean Peninsula, based on literature after Hong (1962a).

No.	No. of genera	No. of taxa	Literature cited
1	53	149	Hong (1962a)
2	41	135	Hong (1966)
3	66	201	Choe (1980)
4	73	207	Kim and Hwang (1991)
5	76	266	Hong (1997)
6	75	236	Yamada and Choe (1997)
7	80	263	Hong (2003)
8	83	285	Park and Choi (2007)
9	87	281	Lee et al. (2011)
10	112	346	Present study

## Materials and methods

**Figure 1.**
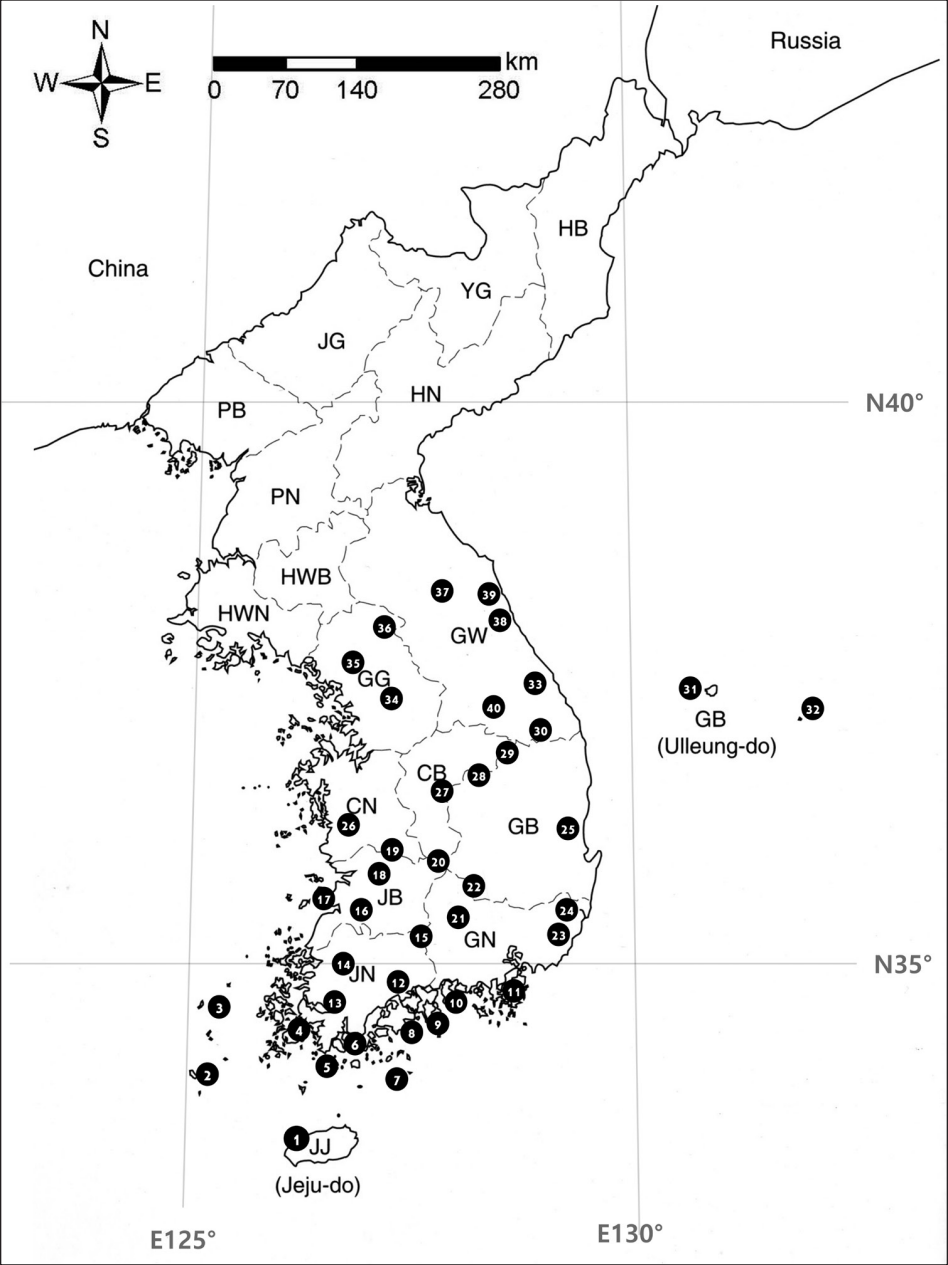
The surveyed areas in the Korean Peninsula

Materials for this study, which include approximately 15,500 specimens, were collected mostly by Choi, with a short participation in the field research by Bakalin between October 2007 and May 2020 at various sites in Korea (Table 3, Figure 1). All the specimens are kept in Jeonbuk National University Herbarium (JNU) and Herbarium of the Botanical Garden-Institute (VBGI). The unsurveyed areas in the present study were investigated by a review of relevant reliable literature (Hong 1997, 2003; Kim and Hwang 1991; Yamada and Choe 1997) and herbarium specimens (G, HIRO, NICH, PE, VLA, VBGI). The analysis of the distribution pattern was based on floristic elements (latitudinal type) and area type (longitudinal types) identified for each occurring species in Bakalin (2010). Nomenclature mostly follows Söderström et al. (2016) with some updates from recent literature (Solenostomataceae (Bakalin et al. 2020a), *Apopellia* (Schütz et al. 2016) etc.). The *Pseudolophozia* concept was followed after Konstantinova and Vilnet (2009) despite in the World liverwort checklist (Söderström et al. 2016) this genus is regarded as the synonym of *Barabilophozia*.

**Table 3.** The surveyed areas in the Korean Peninsula.

No.	Survey area	No.	Survey area	No.	Survey area	No.	Survey area
1	Is. Jeju	11	Is. Geoje	21	Mt. Hwangmae	31	Is. Ulleungdo
2	Is. Gageo	12	Mt. Chogye	22	Mt. Gaya	32	Is. Dokdo
3	Is. Heuksan	13	Mt. Weolchul	23	Mt. Sinbul	33	Mt. Deokhang
4	Mt. Duryun	14	Mt. Samgak	24	Mujechi wetland	34	Mt. Nam
5	Is. Bogil	15	Mt. Jiri	25	Mt. Juwang	35	Mt. Bukhan
6	Is. Wan	16	Mt. Naejang	26	Mt. Gyeryong	36	Mt. Myeongji
7	Is. Geomoon	17	Mt. Naebyeon	27	Mt. Sokri	37	Hwacheon-gun
8	Is. Oenalo	18	Mt. Moak	28	Mt. Weolak	38	Mt. Odae
9	Mt. Geumo	19	Mt. Daedun	29	Mt. Sobaek	39	Mt. Seolak
10	Mt. Geum	20	Mt. Deogyu	30	Mt. Taebaek	40	River Dong

## Results

The liverwort and hornwort flora of Korea includes 346 taxa (326 species, 16 subspecies and four varieties) belonging to 112 genera and 50 families. The list includes 75 taxa of liverworts and four hornworts reported as new to the Korean Peninsula within the last twelve years. Two species of liverworts are endemic to Korea. *Solenostoma jirisanense* Bakalin et Choi S.S. was described as new to science from Mt. Jirisan. The striking characteristics of *S. jirisanense* are red to purple pigmentation shoots, suddenly turned up perianth in the female branches and frequent biconcentric oil-bodies (Bakalin et al. 2020a). *Marsupella koreana* Bakalin et Fedosov was described as new to science from Mt. Jirisan, Mt. Deogyusan and Mt. Gayasan (Bakalin et al. 2019). The striking characteristics of the *M. koreana* are conduplicate leaves, purple pigmentation of shoots, narrowly revolute leaf margin and plants loosely dilated to the perianth.

While compiling the hepatic flora, 42 species, previously reported for Korea, are excluded from the Korean liverwort flora. Amongst them, 14 species (*Calypogeia granulata* Inoue, *Cephalozia hamatiloba* Steph., *Delavayella serrata* Steph., *Gongylanthus ericetorum* (Raddi) Nees, *Jungermannia plagiochilacea* Grolle, *Lopholejeunea subfusca* (Nees) Schiffn., *Marchantia pinnata* Steph., *Metalejeunea cucullata* (Reinw., Blume etNess) Grolle, *Moerckia japonica* Inoue, *Plagiochila delavayi* Steph., *Radula chinensis* Steph., *Riccia frostii* Austin, *Riccia nipponica* S.Hatt. and *Scapania ligulata* Steph.) were reported for the Korean flora by Park and Choi (2007) on an unclear basis (no references to literature were provided at all). Since we were not able to find the location of vouchers and the original sources for these reports, we exclude them from the flora. *Lopholejeunea subfusca* (Nees) Schiffn. was reported by Hong (1962a) from Mt. Jiri. The distribution of *L. subfusca* covers tropical and subtropical areas, so its occurrence in Mt. Jirisan is doubtful. The plants most probably belong to *Acanthocoleus yoshinaganus* (S.Hatt.) Mizut., which is relatively common on Mt. Jiri, but it was not recorded by Hong (1962a). *Porella pinnata* L., *Lejeunea cavifolia* Steph. and *Riccardia incurvata* Lindb. were reported by Kim and Hwang (1991) from the northern part of Korea. However, the distribution of these three species is European-North American and hardly occurred in East Asia. The first one probably belongs to *Porella grandiloba*, whereas the second may belong to *Lejeunea japonica* and the last may belong to any *Riccardia* species (most probably *R. multifida*). See excluded and doubtful records below for the rest of the details.

In the analysis of area types (longitudinal type), 168 taxa (48.6%) are East Asian, 92 (26.6%) are circumpolar, 31 (9.0%) are broadly Asian, 21 (6.1%) are multi-areal and 13 (3.8%) are amphioceanic (Table 4). This result distinctly shows that the Korean Peninsula belongs to the Eastern Asia floristic region (Takhtajan 1986).

In an analysis of flora elements (latitudinal), 82 taxa (23.76%) are temperate, 52 (15.0%) are temperate-subtropical, 49 (14.2%) are subtropical, 48 (13.9%) are arctic-boreal, 32 (9.2%) are boreal-temperate, 28 (8.1%) are boreal and 19 (5.5%) are cosmopolitan (Table 5). Temperate floristic elements occurred in the largest proportion, followed by a large number of temperate-subtropical elements. The subtropical species are mainly distributed in Jeju Island and the southern part of the Korean Peninsula. The arctic-boreal species are mainly distributed in high mountains of the northern part in Korea and rarely distributed on the peaks of Mt. Halla, Mt. Jiri and Mt. Seorak. They presumably should be common in the highest elevations of North Korea, but we do not have any information on this issue.

**Table 4.** The longitude fractional composition of the Korean liverworts and hornworts.

Area type (longitudinal types)	Taxa	Percentage (%)
Amphioceanic	13	3.8
Amphipacific	7	2.0
Asia	31	9.0
Asian-American	6	1.7
Asian-Euro	2	0.6
Circumpolar	92	26.6
Disjunctive	2	0.6
Eastern Asia	168	48.6
Eastern Asia-American	3	0.9
South-eastern Asia	1	0.3
Multi-area	21	6.1

**Table 5.** The latitude fractional composition in Korean liverworts and hornworts within other regions.

Floristic elements (latitudinal types)	Taxa	Percentage (%)
Arctic	22	6.4
Arctic-boreal	48	13.9
Boreal	28	8.1
Boreal-temperate	32	9.2
Temperate	82	23.7
Temperate-subtropical	52	15.0
Subtropical	49	14.2
Subtropical-tropical	14	4.0
Multizone	19	5.5

### Checklist of the Liverworts and Hornworts of the Korean Peninsula (Alphabetical List)

This checklist includes 346 taxa and, amongst them, marked with an asterisk (*), are new records for the Korean Peninsula obtained from 2007. Open circles (°) indicate the species was not collected or observed by ourselves, but found in trustworthy papers. The localities for every species in the Korean Peninsula are listed, based on the specimens identified by the authors and on the following literature cited in brackets. The abbreviations for the provinces of Korean Peninsula are as follows (Figure 1), **CB**: Chungcheongbuk-do, **CN**: Chungcheongnam-do, **GB**: Gyeongsangbuk-do, **GN**: Gyeongsangnam-do, **GG**: Gyeonggi-do, **GW**: Gangwon-do, **HB**: Hamgyeongbuk-do, **HN**: Hamgyeongnam-do, **HWN**: Hwanghaenam-do, **HWB**: Hwanghaebuk-do, **JB**: Jeollabuk-do, **JG**: Jagang-do, **JN**: Jeollanam-do, **JJ**: Jeju-do, **PB**: Pyeonganbuk-do, **PN**: Pyeongannam-do, **YG**: Yanggang-do. The synonyms in the checklist are cited very occasionally if the species were recorded in literature under that name. All specimens examined are deposited in JNU, KB and VBGI. The genera are arranged alphabetically, as are the species within the genus. Nomenclature mostly follows Söderström et al. (2016) with some updates from recent literature mentioned above.

#### Anthocerotae

***Anthoceros* L.** (Anthocerotaceae)

***Anthoceros agrestis*** Paton – **GB** [Ulleung-gun, Jeodong-Dodong, 21 Oct 2010, *Choi 8796* (JNU)], **CN** [Choe 1980, 1983 as *Anthoceros punctatus*], **GW** [Choe 1983 as *Anthoceros punctatus*], **PN** [Kim and Hwang 1991 as *Anthoceros punctatus*], **JG** [Kim and Hwang 1991 as *Anthoceros punctatus*], **HB** [Kim and Hwang 1991 as *Anthoceros punctatus*], **Korea** [Hong 1997, 2003 as *Anthoceros punctatus*]

*Anthoceros angustus Steph. – **JJ** [Jeju-si, Tamla valley, 10 Apr 2012, *Choi 120320* (JNU)]

*Anthoceros subtilis Steph. – **JJ** [Jeju-si, Tamla valley, 10 Apr 2012, *Choi 121015* (JNU)]

***Folioceros* D.C.Bharadwaj** (Anthocerotaceae)

*Folioceros fuciformis (Mont.) D.C.Bhardwaj – **JJ** [Jeju-si, Tamla valley, 25 Sep 2012, *Choi 120971b* (JNU)]

***Notothylas* Sull. ex A.Gray** (Notothylaceae)

***Notothylas orbicularis*** (Schwein.) Sull. – **JJ** [Jeju-do, Che Oreum 19 Jun 2011, *Choi 110955* (JNU)], **CN** [Choe 1975, 1979 as *Notothylas japonica*, Choe 1980, 1983], **PN** [Kim and Hwang 1991], **JG** [Kim and Hwang 1991], **HB** [Kim and Hwang 1991], **Korea** [Choe and Choi 1980, Hong 1997, 2003]

°***Noto thylas temperata*** J.Haseg. – **CN** [Yamada and Choe 2002]

***Phaeoceros* Prosk.** (Notothylaceae)

***Phaeoceros carolinianus*** (Michx.) Prosk. – **JJ** [Jeju-si, Museum of Stones, 9 Aug 2010, *Choi 7865* (JNU); Stephani 1916 as *Anthoceros koreanus*, Hasegawa 1984 as Phaeoceros laevis subsp. carolinianus, Song and Yamada 2006], **JN** [Choe 1975, 1980 as Phaeoceros laevis subsp. carolinianus], **GN** [Choe 1975, 1980 as Phaeoceros laevis subsp. carolinianus], **CN** [Choe 1975, 1980 as Phaeoceros laevis subsp. carolinianus], **GB** [Ulleung-gun, Anpyeongjeon, 20 Oct 2010, *Choi 8709* (JNU); Choe 1975, 1980 as Phaeoceros laevis subsp. carolinianus], **GW** [Inje-gun, Mt. Seolak, Baekdamsa Teample, 14 Oct 2010, *Choi 8660* (JNU)], **Korea** [Schuster 1992b as Phaeoceros laevis subsp. carolinianus, Choe and Choi 1980 as Phaeoceros laevis subsp. carolinianus, Hong 1997, 2003]

***Phaeoceros laevis*** (L.) Prosk. – **JJ** [Seogwipo-si, Andeok valley, 15 Mar 2012, *Choi 120027* (JNU)], **GW** [Kim and Hwang 1991 as *Anthoceros laevis*], **PN** [Kim and Hwang 1991 as *Anthoceros laevis*], **HB** [Kim and Hwang 1991 as *Anthoceros laevis*], **Korea** [Hasegawa 1984 as *Phaeoceros miyakeanus*, Aur and Zhang 1985, Schuster 1992b as *Anthoceors laevis*, Hong 1962a; Hong 1997, 2003]

***Megaceros* Campb.** (Dendrocerotaceae)

*Megaceros flagellaris (Mitt.) Steph. – **JJ** [Seogwipo-si, Dongsu Brigde, 2 Nov 2011, *Choi 111485* (JNU)]

#### Hepaticae

***Acanthocoleus* R.M.Schust.** (Lejeuneaceae)

***Acanthocoleus yoshinaganus*** (S.Hatt.) Kruijt – **JN** [Song and Yamada 2009b as *Dicranolejeunea yoshinagana*], **JB** [Jangsu-gun, Mt. Jangan, Banghwadong valley, 30 Apr 2009, *Choi 3582* (JNU)], **GN** [Hamyang-gun, Mt. Jiri, Chilseon valley, 28 Sep 2010, *Choi 8122* (JNU); Hattori et al. 1962b as *Dicranolejeunea yoshinagana*, Hong 1962a, 1966 as *Lopholejeunea subfusca*], **GB** [Cheongsong-gun, Mt. Juwang, The 2^nd^ waterfall, 8 Nov 2010, *Choi 8919* (JNU)], **CN** [Choe 1980], **GW** [Inje-gun, Mt. Seolak, Baekdamsa Temple, 28 Aug 2009, *Choi 5272* (JNU)], **Korea** [Mizutani 1961 as *Dicranolejeunea yoshinagana*, Inoue 1976 as *Dicranolejeunea yoshinagana*, Choe and Yamada 1974 as *Dicranolejeunea yoshinagana*, Choe and Choi 1980 as *Dicranolejeunea yoshinagana*, Hong 1997, 2003 as *Dicranolejeunea yoshinagana*]

***Acrobolbus* Nees** (Acrobolbaceae)

***Acrobolbus ciliatus*** (Mitt.) Schiffn. – **GN** [Hamyang-gun, Mt. Jiri, Chilseon valley, 28 Sep 2010, *Choi 8144* (JNU)], **GW** [Inje-gun, Mt. Seolak, Socheong shelter valley, 12 May 2011, *Choi 110347* (JNU)], **YG** [Kim and Hwang 1991], **HN** [Gao and Chang 1983a, b], **Korea** [Hong 1997, 2003]

***Acrolejeunea* (Spruce) Schiffn.** (Lejeuneaceae)

***Acrolejeunea pusilla*** (Steph.) Grolle et Gradst. – **JJ** [Jeju-si, *Cryptomeria japonica* forest, 25 Aug 2010, *Choi 8002* (JNU); Song and Yamada 2006], **JN** [Yeoam-gun, Mt. Weolchul, Dogapsa Temple, 1 Dec 2010, *Choi 9056* (JNU); Song and Yamada 2009b], **GN** [Geoje-si, Oryung reservoir, 16 Mar 2011, *Choi 110015* (JNU); Song and Yamada 2009a], **GW** [Yeongweol-gun, River donggang, near the Donggang, 29 Sep 2009, *Choi 5244* (JNU); Kim and Hwang 1991 as *Ptychocoleus nipponicus*], **Korea** [Hong 1997, 2003]

***Acrolejeunea sandvicensis*** (Gottsche) Steph. – **JJ** [Jeju-si, Che Oruem, 27 Aug 2010, *Choi 8063* (JNU); Hong 1966 as *Brachiolejeunea sandvicensis*, Song and Yamada 2006 as *Trocholejeunea sandvicensis*], **JN** [Gurye-gun, Mt. Jiri, Nododan, 5 Aug 2010, *Choi 7549* (JNU); Uno and Takahashi 1940 as *Brachiolejeunea sandvicensis*, Hong 1962a, 1966 as *Brachiolejeunea sandvicensis*, Song and Yamada 2009b as *Trocholejeunea sandvicensis*], **JB** [Buan-gun, Mt. Naebyeon, Jikso waterfall, *Choi 7297* (JNU); Hong 1966 as *Brachiolejeunea sandvicensis*], **GN** [Geoje-si, Oryung reservoir, 16 Mar 2011, *Choi 110003* (JNU); Hattori et al. 1962b as *Brachiolejeunea sandvicensis*, Hong 1962a, 1966 as *Brachiolejeunea sandvicensis*, Song and Yamada 2009a as *Trocholejeunea sandvicensis*], **GB** [Horikawa 1955 as *Brachiolejeunea sandvicensis*], **CN** [Gongju-si, Mt. Gyeryong, Temple Donghaksa valley, 8 Jul 2009, *Choi 4109* (JNU); Choe 1979 as *Trocholejeunea sandvicensis*, Mizutani 1989 as *Trocholejeunea sandvicensis*], **CB** [Hong 1962a, 1962d, 1966 as *Brachiolejeunea sandvicensis*], **GG** [Hong 1960b, 1960c as *Brachiolejeunea sandvicensis*, Hong and Kim 1961a as *Brachiolejeunea sandvicensis*, Hong 1962a, 1966 as *Brachiolejeunea sandvicensis*], **GW** [Inje-gun, Mt. Seolak, Bongjeongam, 14 Oct 2010, *Choi 8575* (JNU); Hong 1962a, 1966 as *Brachiolejeunea sandvicensis*, Gao and Chang 1983a, b as *Trocholejeunea sandvicensis*, Kim et al. 1995 as *Trocholejeunea sandvicensis*], **HWN** [Kim and Hwang 1991 as *Trocholejeunea sandvicensis*], **JG** [Kim and Hwang 1991 as *Trocholejeunea sandvicensis*], **PN** [Gao and Chang 1983a, b as *Trocholejeunea sandvicensis*], **PB** [Kim and Hwang 1991 as *Trocholejeunea sandvicensis*], **YG** [Gao and Chang 1983a, b as *Trocholejeunea sandvicensis*], **Korea** [Mizutani 1961 as *Brachiolejeunea sandvicensis*, Shin 1968, 1970 as *Trocholejeunea sandvicensis*, Iwatsuki and Mizutani 1972 as *Trocholejeunea sandvicensis*, Choe and Yamada 1974 as *Trocholejeunea sandvicensis*, Inoue 1976 as *Trocholejeunea sandvicensis*, Choe and Choi 1980 as *Trocholejeunea sandvicensis*, Gao and Chang 1981 as *Brachiolejeunea sandvicensis*, Miller et al. 1983 as *Trocholejeunea sandvicensis*, Aur and Zhang 1985 as *Trocholejeunea sandvicensis*, Li 1985 as *Trocholejeunea sandvicensis*, Hong 1997, 2003 as *Trocholejeunea sandvicensis*]

***Alobiellopsis* R.M.Schust.** (Cephaloziaceae)

*Alobiellopsis parvifolia (Steph.) R.M.Schust. – **JJ** [Jeju-si, Mulyeongari, 19 Sep 2011, *Choi 110973* (JNU)]

***Anastrepta* (Lindb.) Schiffn.** (Lophoziaceae)

°***Anastrepta orcadensis*** (Hook.) Schiffn. – **HN** [Kim and Hwang 1991], **Korea** [Hong 1997, 2003]

***Anastrophyllum* (Spruce) Steph.** (Lophoziaceae)

***Anastrophyllum assimile*** (Mitt.) Steph. – **JN** [Hong 1966 as *Anastrophyllum reichardtii*, Choe 1975, 1980, 1983 as *Anastrophyllum reichardtii*], **GN** [Geochang-gun, Mt. Namdeogyu, top of mountain, 11 Nov 2010, *Choi 8967* (JNU); Hattori et al. 1962b as *Anastrophyllum reichardtii*, Song and Yamada 2009b], **GW** [Inje-gun, Mt. Seolak, Socheong, 21 Sep 2009, *Choi 5153* (JNU)], **PB** [Kim and Hwang 1991], **YG** [Kim and Hwang 1991], **HN** [Kim and Hwang 1991], **HB** [Kim and Hwang 1991], **Korea** [Grolle 1964; Kitagawa 1966; Schuster 1969; Hattori 1971; Iwatsuki and Mizutani 1972; Choe and Yamada 1974; Hattori 1975a; Choe and Choi 1980 as *Anastrophyllum reichardtii*, Gao and Chang 1981; Aur and Zhang 1985; Long and Grolle 1990; Hong 1997, 2003]

*Anastrophyllum michauxii (F.Weber) H.Buch – **GW** [Inje-gun, Mt. Seolak, Jungcheong, 21 Sep 2009, *Choi 5152* (JNU)]

***Aneura* Dumort.** (Aneuraceae)

*Aneura maxima (Schiffn.) Steph. – **JJ** [Seogwipo-si, Bolrae Oreum, 5 Sep 2012, *Choi 120753* (JNU)], **JN** [Yeoam-gun, Mt. Weolchul, Dogapsa Temple, 1 Dec 2010, *Choi 9074* (JNU)], **GB** [Ulsan-si, Mt. Jeongjok, Mujechi 1 neup, 30 Sep 2010, *Choi 8304* (JNU)].

***Aneura pinguis*** (L.) Dumort. – **JJ** [Jeju-si, Musu stream, Goangryeong 2^nd^ Bridge, 18 Mar 2012, *Choi 120112* (JNU)], **JN** [Haenam-gun, Mt. Duryun, Duryunsa Temple, 23 Apr 2010, *Choi 7311* (JNU)], **JB** [Buan-gun, Mt. Naebyen, Beadrock near road, 10 Mar 2009, *Choi 3379* (JNU)], **GN** [Geoje-si, Oryung reservoir, 16 Mar 2011, *Choi 110027* (JNU)], **GB** [Cheongsong-gun, Mt. Juwang, Weoloe valley, 9 Nov 2010, *Choi 8940* (JNU)], **CN** [Choe 1980], **GG** [Hong 1962a as *Riccardia pinguis*, Choe 1980], **GW** [Sokcho-si, Mt. Seolak, Yangpok shelter area, 13 May 2011, *Choi 110382* (JNU); Kim et al. 1995], **JG** [Kim and Hwang 1991], **YG** [Gao and Chang 1983a, b], **HN** [Kim and Hwang 1991], **HB** [Gao and Chang 1983a, 1983b], **Korea** [Choe and Yamada 1974; Inoue 1976; Miller et al. 1983; Choe and Choi 1980; Hong 1997, 2003]

***Anthelia* (Dumort.) Dumort.** (Antheliaceae)

*Anthelia juratzkana (Limpr.) Trevis. – **JJ** [Seogwipo-si, Baekrokdam, Northwestern wall, 7 Sep 2012, *Choi 120810* (JNU)]

***Apopellia* (Grolle) Nebel & D.Quandt** (Pelliaceae)

***Apopellia endiviifolia*** (Dicks.) Nebel et D.Quandt – **JJ** [Seogwipo-si, Bolrae Oreum, 5 Sep 2012, *Choi 120729* (JNU); Hong 1962a as *Pellia fabbroniana*, Song and Yamada 2006 as *Pellia endiviifolia*], **JN** [Gurye-gun, Mt. Jiri, Nogodan, 19 Sep 2009, *Choi 5045* (JNU); Hong 1962a as *Pellia fabbroniana*, Song and Yamada 2009b as *Pellia endiviifolia*], **JB** [Muju-gun, Mt. Deogyu, 28 Nov 2007, *Choi 10029*; Hong 1962a as *Pellia fabbroniana*], **GN** [Hamyang-gun, Mt. Jiri, Chilseon valley, 28 Sep 2010, *Choi 8141* (JNU)], **GB** [Cheongsong-gun, Mt. Juwang, Weoloe valley, 9 Nov 2010, *Choi 8941* (JNU); Hong 1962a, 1962c as *Pellia fabbroniana*], **CN** [Choe 1979 as *Pellia endiviifolia*], **GG** [Hong and Kim 1961a as *Pellia fabbroniana*, Hong 1962a as *Pellia fabbroniana*], **GW** [Jeongseon-gun, River Donggnag, Limstone, 7 Sep 2011, *Choi 110903*; Hong 1962a as *Pellia fabbroniana*, Kim et al. 1995 as *Pellia endiviifolia*], **PN** [Kim and Hwang 1991 as *Pellia endiviifolia*], **JG** [Kim and Hwang 1991], **HN** [Kim and Hwang 1991 as *Pellia endiviifolia*], **HB** [Gao and Chang 1983a, 1983b as *Pellia endiviifolia*, Kim and Hwang 1991 as *Pellia endiviifolia*], **Korea** [Shin 1968, 1970 as *Pellia endiviifolia*, Choe and Yamada 1974 as *Pellia endiviifolia*, Inoue 1976 as *Pellia endiviifolia*, Choe and Choi 1980 as *Pellia endiviifolia*, Hong 1997, 2003 as *Pellia endiviifolia*]

***Asterella* P.Beauv.** (Aytoniaceae)

***Asterella cruciata*** (Steph.) Horik. – **CN** [Choe 1975 as *Asterella chichibuensis*], **GW** [Yeongweol-gun, River donggang, near the Donggang, 29 Sep 2009, *Choi 5250* (JNU)], **Korea** [Choe 1980 as *Asterella odora*, Choe and Choi 1980 as *Asterella odora*, Hong 1997, 2003 as *Asterella chichibuensis* and *Asterella odora*]

***Asterella leptophylla*** (Mont.) Grolle – **GN** [Sancheong-gun, Mt. Jiri, Jangsanri valley, 13 Jun 2009, *Choi 3675* (JNU)], **GW** [Jeongseon-gun, River Donggang, near ther Donggang, 17 Aug 2010, *Choi 7943*; Horikawa 1936a as *Fimbriaria koreana*, Horikawa 1951a as *Asterella koreana*, Choe 1975, 1980, 1983 as *Asterella koreana*], **Korea** [Choe and Choi 1980 as *Asterella koreana*, Hong 1997, 2003 as *Asterella koreana*]

***Barbilophozia* Loeske** (Anastrophyllaceae)

***Barbilophozia barbata*** (Schmidel ex Schreb.) Loeske – **GW** [Inje-gun, Mt. Seolak, Socheong, 21 Sep 2009, *Choi 5192* (JNU); Kim et al. 1995], **PB** [Kim and Hwang 1991], **JG** [Kim and Hwang 1991], **YG** [Gao and Chang 1983a, 1983b], **HN** [Kim and Hwang 1991], HB [Kim and Hwang 1991], **Korea** [Hong 1997, 2003]

***Bazzania* Gray** (Lepidoziaceae)

***Bazzania denudata*** (Lindenb. et Gottsche) Trevis. – **JJ** [Jeju-si, Mt. Halla, Seongpanak- Baekrokdam, 8 Aug 2010, *Choi 7721* (JNU); Hattori et al. 1962a as *Bazzania ovifolia*, Hong 1962a as *Bazzania ovifolia*, Hong 1966 as Bazzania denudata subsp. ovifolia, Choe 1975, 1983 as *Bazzania ovifolia*, Song and Yamada 2006], **JN** [Sinan-gun, Is. Gageodo, Bolryemi seashore 22 Apr 2012, *Choi 120393* (JNU); Hong 1962a as *Bazzania ovifolia*, Hong 1966 as Bazzania denudata subsp. ovifolia, Choe 1975, 1983 as *Bazzania ovifolia*, Song and Yamada 2009b], **GN** [Geoje-si, Oryung reservoir, 16 Mar 2011, *Choi 110103* (JNU); Hattori et al. 1962b as *Bazzania ovifolia*], **GB** [Ulleung-gun, Seonginbong, 20 Oct 2010, *Choi 8735* (JNU)], **GW** [Inje-gun, Mt. Seolak, Hangyeoryeong, 28 Aug 2009, *Choi 4259* (JNU); Hong and Kim 1960 as *Bazzania ovifolia*, Hong 1960a, 1962a as *Bazzania ovifolia*, Choe 1983 as *Bazzania ovifolia*, Kim et al. 1995], **JG** [Kim and Hwang 1991], **YG** [Kim and Hwang 1991], **HN** [Kim and Hwang 1991], **HB** [Kim and Hwang 1991], **Korea** [Choe and Yamada 1974, Inoue 1974, 1981 as *Bazzania ovifolia*, Choe and Choi 1980 as *Bazzania ovifolia*, Hong 1997]

* ***Bazzania imbricata*** (Mitt.) S.Hatt. – **GN** [Hamyang-gun, Mt. Jiri, Hansin stream, 12 Oct 2019, *Bakalin & Choi Kor-79-6-19* (VBGI)].

*Bazzania japonica (Sande Lac.) Lindb. – **JJ** [Jeju-si, Mt. Halla, Baekrokdam, 21 Sep 2012, *Choi 120888b* (JNU)]

*Bazzania manczurica Bakalin – **GW** [Jeongseon-gun, Gohan-eup, Mt. Hambaek, 23 Jun 2017, *Bum 170346* (JNU)]

*Bazzania parabidentula Bakalin – **JJ** [Hong and Kim 1961b as *Bazzania bidentula*, Hong 1962a, 1966 as *Bazzania bidentula*, Choe 1975, 1980 as *Bazzania bidentula*, Song and Yamada 2006 as *Bazzania bidentula*], **GB** [Choe 1975, 1980 as *Bazzania bidentula*], **GW** [Taebaeksi, Hyeol-dong, Mt. Taebaek, Danggol valley, 24 Jun 2017, *Bum 170378c* (JNU); Hong 1962a, 1966 as *Bazzania bidentula*, Kim et al. 1995 as *Bazzania bidentula*], **JG** [Kim and Hwang 1991 as *Bazzania bidentula*], **YG** [Kim and Hwang 1991 as *Bazzania bidentula*], **HN** [Kim and Hwang 1991 as *Bazzania bidentula*], **HB** [Kim and Hwang 1991 as *Bazzania bidentula*], **Korea** [Reimers 1931 as *Mastiogbryum bidentulum*, Horikawa 1934a as *Bazzania bidentula*, Hattori 1944a, c as *Bazzania bidentula*, Hattori and Mizutani 1958 as *Bazzania bidentula*, Iwatsuki and Mizutani 1972 as *Bazzania bidentula*, Choe and Yamada 1974 as *Bazzania bidentula*, Gao and Chang 1981 as *Bazzania bidentula*, Inoue 1981 as *Bazzania bidentula*, Choe and Choi 1980 as *Bazzania bidentula*, Aur and Zhang 1985 as *Bazzania bidentula*, Li 1985 as *Bazzania bidentula*, Hong 1997, 2003 as *Bazzania bidentula*]

***Bazzania pompeana*** (Sande Lac.) Mitt. – **JJ** [Jeju-si, Musu stream, 28 Oct 2010, *Choi 8848* (JNU); Hattori et al. 1962a; Hong 1962a, 1966; Choe 1980; Song and Yamada 2006], **JN** [Goheung-gun, Is. Oenarodo, Mt. Bongrae, valley, 20 May 2011, *Choi 110591* (JNU); Hong 1966, Choe 1980], **JB** [Jeongeup-si, Mt. Naejang, Geumseon valley, 16 Mar 2009, *Choi 3486* (JNU); Choe 1980], **GN** [Geoje-si, Oryung reservoir, 16 Mar 2011, *Choi 110032*], **CN** [Choe 1979], **Korea** [Choe and Yamada 1974; Choe and Choi 1980; Hong 1997, 2003]

***Bazzania tricrenata*** (Wahlenb.) Lindb. – **JJ** [Jeju-si, Mt. Halla, Baekrokdam, 8 Aug 2010, *Choi 7752* (JNU); Hong and Kim 1961b, Hattori et al. 1962a, Hong 1966, Choe 1975, 1980, Song and Yamada 2006], **JN** [Gurye-gun, Mt. Jiri, Nogodan, 29 Apr 2009, *Choi 3571* (JNU); Hong 1966, Song and Yamada 2009b], **GN** [Hamyang-gun, Mt. Jiri, Cheonwnagbong, 29 Sep 2010, *Choi 8267* (JNU); Hattori et al. 1962b, Choe 1975, 1980], **GB** [Choe 1980], **GW** [Inje-gun, Mt. Seolak, Jungcheong, 21 Sep 2009, *Choi 5120* (JNU); Hong 1966, Kim et al. 1995], **YG** [Gao and Chang 1983a, 1983b], **HN** [Kim and Hwang 1991], **HB** [Kim and Hwang 1991], **Korea** [Iwatsuki and Mizutani 1972; Choe and Yamada 1974; Choe and Choi 1980; Gao and Chang 1981; Aur and Zhang 1985; Li 1985; Hong 1997, 2003]

***Bazzania tridens*** (Reinw., Blume et Nees) Trevis. – **JJ** [Seogwipo-si, Is. Beomseom, 21 Mar 2012, *Choi 120245* (JNU)], **JN** [Jindo-gun, Mt. Yegwi, ridge, 11 Feb 2010, *Choi 7112* (JNU)], **GW** [Kim and Hwang 1991], **Korea** [Stephani 1924 as *Mastigobryum coreanum*, Hattori and Mizutani 1958 as *Mastigobryum coreanum* and *Bazzania albicans*, Hong 1962a as *Bazzania albicans*, Shin 1968, 1970 as *Bazzania albicans*, Kitagawa 1972, 1978; Choe and Yamada 1974; Choe and Choi 1980; Choe 1983; Miller et al. 1983; Hong 1997, 2003]

***Bazzania trilobata*** (L.) Gray – **GN** [Hamyang-gun, Mt. Jiri, Chilseon valley, 28 Sep 2010, *Choi 8159* (JNU)], **GG** [Hong 1960c, Choe 1980], **GW** [Inje-gun, Mt. Seolak, Bongjeongam, 14 Oct 2010, *Choi 8592* (JNU); Hong and Kim 1960; Hong 1962a, 1966; Choe 1975, 1980; Kim and Hwang 1991; Kim et al. 1995], **YG** [Kim and Hwang 1991], **Korea** [Choe and Yamada 1974; Choe and Choi 1980; Kitagawa 1981; Hong 1997, 2003]

***Biantheridion* (Grolle) Konstant. et Vilnet** (Anastrophyllaceae)

°***Biantheridion undulifolium*** (Nees) Konstant. et Vilnet –**YG** [Gao and Chang 1983a, 1983b as *Jamesoniella undulifolia* (Nees) Müll.Frib., Kim and Hwang 1991 as *Jamesoniella undulifolia* (Nees) Müll.Frib.]

***Blasia* L.** (Blasiaceae)

***Blasia pusilla*** L. – **JJ** [Jeju-si, Tamla valley, 10 Apr 2012, *Choi 120305* (JNU)], **GB** [Hong 1962a, Choe 1980], **GG** [Hong and Kim 1961a; Hong 1962a, c; Choe 1980], **GW** [Hong and Kim 1960; Hong 1962a; Choe 1980], **HB** [Kim and Hwang 1991], **Korea** [Choe and Yamada 1974; Choe and Choi 1980; Gao and Chang 1981; Aur and Zhang 1985; Schuster 1992a; Hong 1997, 2003]

***Blepharostoma* (Dumort.) Dumort.** (Pseudolepicoleaceae)

*Blepharostoma epilithica Vilnet et Bakalin – **GW** [Inje-gun, Mt. Seolak, *Bakalin Kor-11-16-11* (VBGI); Bakalin et al. 2020b]

***Blepharostoma minor*** Horik. – **JJ** [Jeju-si, Musu stream, 28 Oct 2010, *Choi 8834* (JNU); Horikawa 1951b; Hattori et al 1962a; Hong 1966; Choe 1980; Song and Yamada 2006], **JN** [Goheung-gun, Is. Seongdudo, shoreline, 20 May 2011, *Choi 110608* (JNU); Hong 1966; Song and Yamada 2009b], **JB** [Hong 1966, Choe1980], **GN** [Geoje-si, Mt. Noja, Forest lodge, 17 Mar 2011, *Choi 110055* (JNU); Hattori et al. 1962b; Choe 1980], **GB** [Ulleung-gun, Seonginbong, 20 Oct 2010, *Choi 8742* (JNU); Hong 1966; Choe 1980], **CN** [Choe 1979], CB [Hong 1962d, 1966], **GG** [Hong 1966, Choe 1980], **GW** [Inje-gun, Mt. Seolak, Hangyeoryeong, 21 Sep 2009, *Choi 5060* (JNU); Horikawa 1951b, Kim et al. 1995], **HWN** [Kim and Hwang 1991], **PB** [Kim and Hwang 1991], **YG** [Kim and Hwang 1991], **HN** [Kim and Hwang 1991], **HB** [Kim and Hwang 1991], **Korea** [Shin 1968, 1970; Choe and Yamada 1974; Choe and Choi 1980; Hong 1997, 2003]

***Blepharostoma trichophyllum*** (L.) Dumort. – **JJ** [Jeju-si, Jindalrae shelter, 28 Oct 2011, *Choi 111189* (JNU); Horikawa 1934d, 1935b, 1951b; Hong and Kim 1961b; Hattori et al 1962a; Hong 1962a, 1966; Choe 1980; Song and Yamada 2006], **JN** [Hong 1966, Song and Yamada 2009b], **GN** [Geochang-gun, Mt. Namdeogyu, top of mountain, 11 Nov 2010, *Choi 8982* (JNU); Hong and Yoo 1961; Hattori et al. 1962b; Hong 1962a; Choe 1980], **GB** [Hong 1962a, 1966], **CB** [Hong 1962a], **GG** [Hong 1962a, 1966; Choe 1980], **GW** [Inje-gun, Mt. Seolak, Hangyeoryeong, 28 Aug 2009, *Choi 4277* (JNU); Hong 1962a, 1966; Kim et al. 1995], **HWN** [Kim and Hwang 1991], **PB** [Kim and Hwang 1991], **YG** [Kim and Hwang 1991], **HN** [Kim and Hwang 1991], **HB** [Horikawa 1951a], **Korea** [Reimers 1931; Horikawa 1939; Hattori 1944a, 1971; Iwatsuki and Mizutani 1972; Choe and Yamada 1974; Choe and Choi 1980; Gao and Chang 1981; Hong 1997, 2003]

***Calycularia* Mitt.** (Calyculariaceae)

***Calycularia laxa*** Lindb. et Arnell – **JJ** [Jeju-si, Mt. Halla, Baekrokdam, 8 Aug 2010, *Choi 7760* (JNU)], **JN** [Gurye-gun, Mt. Jiri, Nogodan, 29 Apr 2009, *Choi 3534* (JNU); Choe 1983 as *Calycularia crispula*, Song and Yamada 2009b as *Calycularia crispula*], **GN** [Geochang-gun, Mt. Namdeogyu, top of mountain, 11 Nov 2010, *Choi 8964* (JNU); Hattori et al. l962b as *Calycularia crispula*, Choe 1980, 1983], **GW** [Inje-gun, Mt. Seolak, Bongjeongam, 14 Oct 2010, *Choi 8599* (JNU)] **JG** [Kim and Hwang 1991 as *Calycularia crispula*], **Korea** [Iwatsuki and Mizutani 1972 as *Calycularia crispula*, Inoue 1976, 1981 as as *Calycularia crispula*, Choe and Yamada 1974 as *Calycularia crispula*, Choe and Choi 1980 as *Calycularia crispula*, Hong 1997, 2003 as *Calycularia crispula*]

***Calypogeia* Raddi** (Calypogeiaceae)

*Calypogeia angusta Steph. – **GN** [Sancheong-gun, Mt. Jiri, Rotari shelter, 14 Jun 2009, *Choi 3689a* (JNU)]

***Calypogeia arguta*** Nees et Mont. – **JJ** [Jeju-si, *Cryptomeria japonica* forest, 25 Aug 2010, *Choi 8003* (JNU); Hattori et al. 1962a; Hong 1962a, 1966; Choe 1975, 1980; Song and Yamada 2006], **JN** [Goheung-gun, Is. Oenarodo, Mt. Bongrae, valley, 20 May 2011, *Choi 110598* (JNU); Song and Yamada 2009b], **JB** [Buan-gun, Mt. Naebyen, Jikso Waterfall, 10 Mar 2009, *Choi 3306* (JNU)], **GN** [Geochang-gun, Mt. Namdeogyu, top of mountain, 11 Nov 2010, *Choi 8970* (JNU); Song and Yamada 2009a], **GB** [Ulsans-si, Mt. Jeongjok, Mujechi 1 neup, 30 Sep 2010, *Choi 8314* (JNU); Hong 1962a, c, 1966; Choe 1975], **CN** [Gongju-si, Mt. Gyeryong, Temple Donghaksa valley, 8 Jul 2009, *Choi 4390* (JNU)], **CB** [Choe 1980], **GG** [Hong 1962a, 1966; Choe 1975, 1980], **GW** [Inje-gun, Mt. Seolak, Hangyeoryeong, 28 Aug 2009, *Choi 4260* (JNU); Kim et al. 1995], **PN** [Kim and Hwang 1991], **JG** [Kim and Hwang 1991], **YG** [Kim and Hwang 1991], **HN** [Kim and Hwang 1991], **HB** [Kim and Hwang 1991], **Korea** [Choe and Yamada 1974; Choe and Choi 1980; Miller et al. 1983; Hong 1997, 2003]

*Calypogeia japonica Steph. – **JJ** [Seogwipo-si, Erimok valley, 6 Sep 2012, *Choi 120785* (JNU)]

***Calypogeia neesiana*** (C.Massal. et Carestia) Müll.Frib. – **GB** [Ulleung-gun, Seonginbong, 20 Oct 2010, *Choi 8715* (JNU)], **GW** [Kim et al. 1995], **PB** [Kim and Hwang 1991], **JG** [Kim and Hwang 1991], **YG** [Kim and Hwang 1991], **HN** [Kim and Hwang 1991], **HB** [Kim and Hwang 1991], **Korea** [Hong 1997, 2003]

***Calypogeia orientalis*** Buczkowska et Bakalin – **JJ** [Hong 1966 as *Calypogeia trichomanis*, Choe 1975, 1980 as *Calypogeia trichomanis*, Song and Yamada 2006 as *Calypogeia azurea*], **JN** [Hong 1962a, 1966 as *Calypogeia trichomanis*, Song and Yamada 2009b as *Calypogeia azurea*], **GN** [Sancheong-gun, Mt. Jiri, Hakseupwon area, 14 Jun 2009, *Choi 3685* (JNU); Choe 1975, 1980 as *Calypogeia trichomanis*], **GW** [Inje-gun, Mt. Seolak, Bongjeongam valley, 11 May 2011, *Choi 110199* (JNU)], **PB** [Kim and Hwang 1991 as *Calypogeia trichomanis*], **YG** [Kim and Hwang 1991 as *Calypogeia trichomanis*], **HN** [Kim and Hwang 1991 as *Calypogeia trichomanis*], **HB** [Kim and Hwang 1991 as *Calypogeia trichomanis*], **Korea** [Choe and Yamada 1974 as *Calypogeia trichomanis*, Choe and Choi 1980 as *Calypogeia trichomanis*, Hong 1997, 2003 as *Calypogeia azurea*]

***Calypogeia tosana*** (Steph.) Steph. – **JJ** [Jeju-si, Sumeunmulbyengdwi, 26 Aug 2010, *Choi 8011* (JNU); Hattori et al. 1962a; Hong 1962a as, Hong 1966; Miller et al.1983; Song and Yamada 2006], **JN** [Goheung-gun, Is. Seongdudo, shoreline, 20 May 2011, *Choi 110612* (JNU); Hong 1962a], **JB** [Buan-gun, Mt. Naebyen, Namyeochi, 10 Mar 2009, *Choi 3332* (JNU)], **GN** [Hamyang-gun, Mt. Jiri, Chilseon valley, 28 Sep 2010, *Choi 8150* (JNU)], **GB** [Ulsans-si, Mt. Jeongjok, Mujechi 4 neup, 30 Sep 2010, *Choi 8302* (JNU); Hong 1962c, 1966], **CN** [Nonsan-si, Mt. Daedun, Surak valley, 31 Mar 2009, *Choi 3423* (JNU)], **GG** [Hong and Kim 1961a, Hong 1962a, 1966], **GW** [Inje-gun, Mt. Seolak, Hangyeoryeong, 28 Aug 2009, *Choi 4261* (JNU); Hong 1962a, 1966, Kim and Hwang 1991, Kim et al. 1995], **HB** [Kim and Hwang 1991], **Korea** [Shin 1970; Inoue 1974, 1981; Choe and Yamada 1974; Choe and Choi 1980; Hong 1997, 2003]

°***Calypogeia yoshinagana*** Steph. – **JJ** [Hattori et al. 1962a as Calypogeia tosana var. yoshinagana, Hong 1962a as Calypogeia tosana var. yoshinagana], **Korea** [Choe and Yamada 1974 as Calypogeia tosana var. yoshinagana]

***Cavicularia* Steph.** (Blasiaceae)

*Cavicularia densa Steph. – **JJ** [Jeju-si, Goangryeongcheon stream, 14 May 2012, C*hoi 120467* (JNU)]

***Cephalozia* (Dumort.) Dumort.** (Cephaloziaceae)

°***Cephalozia ambigua*** C.Massal. – **GW** [Kim and Hwang 1991], **PB** [Kim and Hwang 1991], **YG** [Kim and Hwang 1991], **HN** [Kim and Hwang 1991], **Korea** [Hong 1997, 2003]

***Cephalozia bicuspidata*** (L.) Dumort. – **JJ** [Jeju-si, Mt. Halla, Baekrokdam, 21 Sep 2012, *Choi 120920* (JNU)], **JB** [Namwon-si, Mt. Jiri, Jeongryeongchi area, 26 Aug 2009, *Choi 4230* (JNU)], **GN** [Geochang-gun, Mt. Namdeogyu, top of mountain, 11 Nov 2010, *Choi 8963* (JNU)], **GW** [Inje-gun, Mt. Seolak, Socheong, 21 Sep 2009, *Choi 5166* (JNU)], **Korea** [Hong 1997, 2003]

***Cephalozia lacinulata*** (J.B.Jack ex Gottsche et Rabenh.) Spruce – **JJ** [Jeju-si, Jindalrae shelter, 28 Oct 2011, *Choi 111178* (JNU)], **GW** [Inje-gun, Mt. Seolak, Hangyeoryeong, 28 Aug 2009, *Choi 4268* (JNU); Gao and Chang 1983a, b; Kim and Hwang 1991; Kim et al. 1995], **YG** [Gao and Chang 1983a, b; Kim and Hwang 1991], **HN** [Kim and Hwang 1991], **Korea** [Hong 1997, 2003]

***Cephalozia otaruensis*** Steph. – **JJ** [Jeju-si, Jindalrae shelter, 28 Oct 2011, *Choi 111215* (JNU); Song and Yamada 2006], **JN** [Goheung-gun, Is. Seongdudo, shoreline, 20 May 2011, *Choi 110614* (JNU)], **JB** [Jeongeup-si, Mt. Naejang, Meokbaengigol valley, 28 Jun 2010, *Choi 7433* (JNU)], **GN** [Hamyang-gun, Mt. Jiri, Chilseon valley, 28 Sep 2010, *Choi 8115* (JNU); Hattori et al. 1962b as Cephalozia bicuspidata subsp. otaruensis, Song and Yamada 2009b], **GB** [Ulsans-si, Mt. Jeongjok, Mujechi 4 neup, 30 Sep 2010, *Choi 8303* (JNU)], **CN** [Choe 1979 as Cephalozia bicuspidata subsp. otaruensis], **GG** [Hong 1962a, 1966 as Cephalozia bicuspidata subsp. otaruensis, Choe 1980 as Cephalozia bicuspidata subsp. otaruensis], **GW** [Inje-gun, Mt. Seolak, Jungcheong, 21 Sep 2009, *Choi 5127*; Choe 1980 as Cephalozia bicuspidata subsp. otaruensis], **JG** [Kim and Hwang 1991], **HN** [Kim and Hwang 1991], **HB** [Kim and Hwang 1991], **Korea** [Inoue 1974, 1981 as *Cephalozia otaruensis*Choe and Yamada 1974; Choe and Choi 1980 as Cephalozia bicuspidata subsp. otaruensis, Hong 1997, 2003]

***Cephaloziella* (Spruce) Schiffn.** (Cephaloziellaceae)

***Cephaloziella divaricata*** (Sm.) Schiffn. – **JN** [Wando-gun, Is. Bogil, ridge, 16 Nov 2010, *Choi 9011* (JNU)], **GN** [Sancheong-gun, Mt. Jiri, below Cheonwangbong, 15 Jun 2009, *Choi 3786* (JNU)], **GB** [Hong 1966 as *Cephaloziella byssacea*, Choe 1980, 1983 as *Cephaloziella byssacea*], **GW** [Inje-gun, Mt. Seolak, Socheong, 21 Sep 2009, *Choi 5186* (JNU)], **Korea** [Choe and Choi 1980 as *Cephaloziella byssacea*, Hong 1997, 2003]

*Cephaloziella hampeana (Nees) Schiffn. ex Loeske – **JN** [Gurye-gun, Mt. Jiri, Nododan, 5 Aug 2010, *Choi 7558* (JNU)], **JB** [Namwon-si, Mt. Jiri, Jeongryeongchi area, 26 Aug 2009, *Choi 4236* (JNU)], **GN** [Geochang-gun, Mt. Namdeogyu, top of mountain, 11 Nov 2010, *Choi 8956* (JNU)], **GW** [Inje-gun, Mt. Seolak, Hangyeoryeong, 21 Sep 2009, *Choi 5052* (JNU)].

*Cephaloziella massalogi (Spruce) Müll.Frib. – **JN** [Goheung-gun, Is. Oenarodo, Mt. Bongrae, valley, 20 May 2011, *Choi 110592* (JNU)], **GN** [Hapcheon-gun, Mt. Hangmae, ridge, 9 Sep 2009, *Choi 4389* (JNU)].

***Cephaloziella microphylla*** (Steph.) Douin – **JJ** [Seogwipo-si, Hyodon stream, 7 Aug 2010, *Choi 7601* (JNU)], **JN** [Goangyang-si, Mt. Baekun, 1 Aug 2009, *Choi 4199* (JNU)], **JB** [Namwon-si, Mt. Jiri, Baemsagol valley, 19 Jun 2009, *Choi 3887* (JNU)], **GN** [Hamcheon-gun, Mt. Hwangmae, ridge, 3 Aug 2010, *Choi 7486* (JNU)], **CN** [Gongju-si, Mt. Gyeryong, Temple Donghaksa valley, 8 Jul 2009, *Choi 4089* (JNU); Choe 1980], **GG** [Choe 1980], **GW** [Inje-gun, Mt. Seolak, Jungcheong, 21 Sep 2009, *Choi 5140* (JNU); Kim and Hwang 1991, Kim et al. 1995], **Korea** [Choe and Choi 1980; Hong 1997, 2003]

***Cephaloziella spinicaulis*** Douin – **JJ** [Jeju-si, Musu stream, Goangryeong 2^nd^ Bridge, 17 Mar 2012, *Choi 120062* (JNU); Hattori et al. 1962a as *Cephaloziella echinata*, Kitagawa 1965a, Song and Yamada 2006], **JN** [Goangyang-si, Mt. Baekun, 1 Aug 2009, *Choi 4198* (JNU); Song and Yamada 2009b], **JB** [Buan-gun, Mt. Naebyen, Beadrock near road, 10 Mar 2009, *Choi 3357* (JNU)], **GN** [Geoje-si, Oryung reservoir, 16 Mar 2011, *Choi 110004* (JNU); Hong 1962a, 1966 as *Cephaloziella echinata*, Choe 1980, Song and Yamada 2009a], **CN** [Gongju-si, Mt. Gyeryong, Temple Donghaksa valley, 8 Jul 2009, *Choi 4095* (JNU); Choe 1979, 1980], **GW** [Taebaek-si, Mt. Taebaek, Janggunbong, 15 Sep 2009, *Choi 4443* (JNU); Kim et al. 1995], **PB** [Kim and Hwang 1991], **HN** [Kim and Hwang 1991], **Korea** [Iwatsuki and Mizutani 1972; Choe and Yamada 1974; Choe and Choi 1980; Hong 1997, 2003]

***Cephaloziella spinigera*** (Lindb.) Jørg. – **JJ** [Seogwipo-si, Bolrae Oreum, 5 Sep 2012, *Choi 120749* (JNU)], **GN** [Hamcheon-gun, Mt. Hwangmae, ridge, 3 Aug 2010, *Choi 7499* (JNU)], **GG** [Choe and Yamada 1997 as *Cephaloziella subdentata*], **Korea** [Hong 2003]

*Cephaloziella varians (Gottsche) Steph. – **JN** [Jangheung-gun, Mt. Cheongoan, top of mountain, 19 May 2011, *Choi 110533* (JNU)], **GN** [Namhae-gun, Mt. Geum, top of mountain, 21 May 2011, *Choi 110649*]

***Cheilolejeunea* (Spruce) Steph.** (Lejeuneaceae)

*Cheilolejeunea japonica (Horik.) W.Ye et R.L.Zhu – **GW** [Sokcho-si, Mt. Seolak, 9 Sep 2009, *Choi 5142* (JNU)]

***Cheilolejeunea nipponica*** (S.Hatt.) S.Hatt. – **JJ** [Jeju-si, Musu stream, Goangryeong 2^nd^ Bridge, 18 Mar 2012, *Choi 120106* (JNU); Song and Yamada 2006]

***Cheilolejeunea obtusifolia*** (Steph.) S.Hatt. – **JJ** [Jeju-si, Musu stream, Goangryeong 2^nd^ Bridge, 18 Mar 2012, *Choi 120133* (JNU)], **JN** [Jangheung-gun, Mt. Cheongoan, top of mountain, 19 May 2011, *Choi 110542* (JNU); Hong 1966, Song and Yamada 2009b], **JB** [Buan-gun, Mt. Naebyen, Jikso Waterfall, 10 Mar 2009, *Choi 3305* (JNU); Hong 1966], **GN** [Geochang-gun, Mt. Namdeogyu, top of mountain, 11 Nov 2010, *Choi 8952* (JNU); Hattori et al. 1962b], **GB** [Cheongsong-gun, Mt. Juwang, Jeolgol, 9 Nov 2010, *Choi 8922* (JNU)], **CN** [Gongju-si, Mt. Gyeryong, Temple Donghaksa valley, 8 Jul 2009, *Choi 4078* (JNU); Choe 1979], **GG** [Hong 1966], **GW** [Inje-gun, Mt. Seolak, Hangyeoryeong, 21 Sep 2009, *Choi 5051* (JNU); Hong 1966, Kim and Hwang 1991], **Korea** [Shin 1968, 1970; Mizutani 1961; Iwatsuki and Mizutani 1972; Choe and Yamada 1974; Choe and Choi 1980; Hong 1997, 2003]

°***Cheilolejeunea obtusilobula*** (S.Hatt.) S.Hatt. – **GG** [Hong 1962a, 1966], **Korea** [Miller et al. 1983; Hong 1997, 2003]

***Cheilolejeunea trapezia*** (Nees) Kachroo et R.M.Schust. – **JJ** [Seogwipo-si, Is. Beomseom, 21 Mar 2012, *Choi 120235* (JNU); Hong 1966 as *Cheilolejeunea imbricata*, Choe 1980, 1983 as *Cheilolejeunea imbricata*, Song and Yamada 2006], **JN** [Jindo-gun, Is. Gwanmae, 23 Nov 2008, *Choi 20081123-94-02* (JNU)], **Korea** [Choe and Choi 1980 as *Cheilolejeunea imbricata*, Hong 1997, 2003 as *Cheilolejeunea imbricata*]

***Chiastocaulon* Carl** (Plagiochilaceae)

°***Chiastocaulon dendroides*** (Nees) Carl – **PN** [Gao and Chang 1983a, b as *Plagiochila dendroides*], **JG** [Kim and Hwang 1991 as *Plagiochila dendroides*], **Korea** [Hong 1997, 2003 as *Plagiochila dendroides*]

***Chiloscyphus* Corda** (Lophocoleaceae)

***Chiloscyphus pallescens*** (Ehrh.) Dumort. – **JN** [Gurye-gun, Mt. Jiri, Nogodan, 29 Apr 2009, *Choi 3553* (JNU)], **JB** [Buan-gun, Mt. Naebyen, Beadrock near road, 10 Mar 2009, *Choi 3364* (JNU)], **GN** [Hamyang-gun, Mt. Jiri, Chilseon valley, 28 Sep 2010, *Choi 8194* (JNU)], **CB** [Hong 1966; Choe 1980], **GW** [Hong 1966], **PN** [Kim and Hwang 1991], **JG** [Kim and Hwang 1991], **HB** [Gao and Chang 1983a, b], **Korea** [Choe and Choi 1980; Long and Grolle 1990; Hong 1997, 2003]

***Chiloscyphus polyanthos*** (L.) Corda – **JJ** [Jeju-si, Hancheon Bridge, 14 Mar 2012, *Choi 120001* (JNU); Horikawa 1934c; Hattori et al. 1962a; Song and Yamada 2006], **JN** [Goheung-gun, Mt. Palyeoung, Forest lodge valley, 23 Jun 2009, *Choi 4015* (JNU); Hong 1962a, Hong 1966, Choe 1980, 1983 as *Jungermannia polyanthos*, Song and Yamada 2009b], **JB** [Jangsu-gun, Mt. Waryong, Forest lodge, 10 May 2012, *Choi 120580* (JNU)], **GN** [Geoje-si, Oryung reservoir, 16 Mar 2011, *Choi 110024* (JNU); Horikawa 1934c, Hattori et al. 1962b, Hong 1962a, 1966, Song and Yamada 2009a], **GB** [Hong 1962a, 1962c, 1966], **CN** [Choe 1979], **CB** [Hong 1962d, 1966], **GG** [Hong 1960c; Hong and Kim 1961a; Hong 1962a, 1966], **GW** [Inje-gun, Mt. Seolak, Seolak waterfall, 21 Sep 2009, *Choi 5220* (JNU); Hong and Kim 1960; Hong 1962a, 1966; Gao and Chang 1983a, b; Kim et al. 1995], **HWN** [Kim and Hwang 1991], **HWB** [Kim and Hwang 1991], **PB** [Kim and Hwang 1991], **JG** [Kim and Hwang 1991], **HB** [Kim and Hwang 1991], **Korea** [Horikawa 1934d; Choe and Yamada 1974; Hattori 1975a; Gao and Chang 1981; Aur and Zhang 1985; Choe and Choi 1980 as *Jungermannia polyanthos*, Hong 1997, 2003]

***Clevea* Lindb.** (Cleveaceae)

*Clevea nana (Shimizu et S.Hatt.) Borovich. et Bakalin – **GW** [Yeongweol-gun, River Donggang, 17 Aug 2010, *Choi 7944* (JNU)]

***Cololejeunea* (Spruce) Steph.** (Lejeuneaceae)

°***Cololejeunea denticulata*** (Horik.) S.Hatt. – **GW** [Kim et al. 1995], Korea [Hong 1997, 2003]

***Cololejeunea japonica*** (Schiffn.) Mizut. – **JJ** [Jeju-si, Museum of Stones, 9 Aug 2010, *Choi 7777* (JNU); Song and Yamada 2006], **JN** [Goheung-gun, Is. Seongdudo, shoreline, 20 May 2011, *Choi 110607* (JNU); Choe 1980, 1983], **JB** [Jeongeup-si, Mt. Naejang, Geumseon valley, 14 Mar 2010, *Choi 7255* (JNU)], **GN** [Song and Yamada 2009a], **Korea** [Kitagawa 1981; Choe and Choi 1980; Hong 1997, 2003]

°***Cololejeunea kodamae*** Kamim. – **JJ** [Hong and Kim 1961b; Hattori et al. 1962a; Hong 1962a, 1966; Inoue 1976; Choe 1980; Song and Yamada 2006], **GG** [Hong 1966; Choe 1980], **Korea** [Mizutani 1961; Choe and Yamada 1974; Choe and Choi 1980; Hong 1997, 2003]

***Cololejeunea longifolia*** (Mitt.) Benedix ex Mizut. – **JJ** [Jeju-si, Musu stream, 28 Oct 2010, *Choi 8822* (JNU)], **JN** [Haenam-gun, Mt. Duryun, Taehengsa Temple valley, 18 May 2011, *Choi 110451* (JNU)], **JB** [Buan-gun, Mt. Naebyen, Namyeochi, 10 Mar 2009, *Choi 3336* (JNU)], **CN** [Choe 1980], **GN** [Geoje-si, Oryung reservoir, 16 Mar 2011, *Choi 110017* (JNU); Horikawa 1934b as *Physocolea leptolejeuneoides*], **GB** [Cheongsong-gun, Mt. Juwang, The 2^nd^ waterfall, 8 Nov 2010, *Choi 8915* (JNU)], **GG** [Hong 1962a, 1966 as *Cololejeunea minuta*, Choe 1980], **GW** [Pyeochang-gun, Mt. Odae, Jinbugogye, 29 Aug 2009, *Choi 4287* (JNU); Kim and Hwang 1991, Kim et al. 1995], **PB** [Kim and Hwang 1991], **Korea** [Hattori 1944a as *Cololejeunea minuta*, Choe and Yamada 1974; Choe and Choi 1980; Hong 1997, 2003]

***Cololejeunea macounii*** (Spruce) A.Evans – **JJ** [Jeju-si, Mt. Halla, Baekrokdam, 8 Aug 2010, *Choi 7756* (JNU); Hong and Kim 1961b; Hattori et al. 1962a; Hong 1962a, 1966; Choe 1980, 1983; Song and Yamada 2006], **GN** [Geochang-gun, Mt. Namdeogyu, top of mountain, 11 Nov 2010, *Choi 8986* (JNU)], **GW** [Inje-gun, Mt. Seolak, Taecheongbong, 21 Sep 2009, *Choi 5207* (JNU)], **HWB** [Kim and Hwang 1991], **Korea** [Shin 1968; Iwatsuki and Mizutani 1972; Choe and Yamada 1974; Choe and Choi 1980; Hong 1997, 2003]

***Cololejeunea ornata*** A.Evans – **JJ** [Jeju-si, Gwangryeongcheon stream, 4 May 2012, *Choi 120471* (JNU)], **GW** [Hong 1962a, b, 1966; Choe 1980, 1983], **YG** [Kim and Hwang 1991], **HN** [Kim and Hwang 1991], **Korea** [Mizutani 1961; Choe and Yamada 1974; Choe and Choi 1980; Hong 1997, 2003]

***Cololejeunea planissima*** (Mitt.) Abeyw. – **JJ** [Horikawa 1939 as *Cololejeunea aoshimensis*, Hattori 1942 as *Cololejeunea aoshimensis*, Chen and Wu 1964 as *Pedinolejeunea aochimensis*, Hong 1962a, 1966 as *Cololejeunea aoshimensis*, Li 1985 as *Pedinolejeunea aoshimensis*, Song and Yamada 2006], **JN** [Goheung-gun, Is. Oenarodo, shoreline, 20 May 2011, *Choi 110603* (JNU)], **GN** [Geoje-si, seashore, 9 Jan 2008, *Choi 20080109-02-10* (JNU)], **Korea** [Shin 1968, 1970 as *Cololejeunea aoshimensis*, Choe and Yamada 1974 as *Cololejeunea aoshimensis*, Choe and Choi 1980 as *Cololejeunea aoshimensis*, Choe 1980, 1983 as *Cololejeunea aoshimensis*, Zhu and Wang 1992; Hong 1997, 2003,]

***Cololejeunea raduliloba*** Steph. – **JJ** [Horikawa 1934b as *Leptocolea longilobula*, Hattori 1942 as *Leptocolea longilobula*, Mizutani 1961; Hong 1962a; Choe 1980, 1983; Song and Yamada 2006], **JN** [Wando-gun, Sangwhangbong, valley, 9 Feb 2010, *Choi 3204* (JNU)], **GN** [Geoje-si, Oryung reservoir, 16 Mar 2011, *Choi 110016* (JNU)], **Korea** [Shin 1968, 1970; Choe and Yamada 1974; Choe and Choi 1980; Hong 1997]

***Cololejeunea shikokiana*** (Horik.) S.Hatt. – **JJ** [Choe 1980, 1983; Song and Yamada 2006], **JN** [Yeoam-gun, Mt. Weolchul, Dogapsa Temple, 1 Dec 2010, *Choi 2006* (JNU)], **Korea** [Choe and Choi 1980; Hong 1997, 2003]

***Cololejeunea spinosa*** (Horik.) Pandé et R.N.Misra – **JN** [Wando-gun, Sangwhangbong, valley, 9 Feb 2010, *Choi 7022* (JNU)], **GW** [Kim and Hwang 1991; Kim et al. 1995], **Korea** [Hong 1997, 2003]

***Cololejeunea subkodamae*** Mizut. – **JJ** [Jeju-si, Musu stream, Goangryeong 2^nd^ Bridge, 17 Mar 2012, *Choi 120063* (JNU)], **JN** [Yeoam-gun, Mt. Weolchul, Dogapsa Temple, 1 Dec 2010, *Choi 9072* (JNU)], **GN** [Geoje-si, Oryung reservoir, 16 Mar 2011, *Choi 110005* (JNU)], **GG** [Yamada and Choe 1997], **HWN** [Kim and Hwang 1991]

°***Cololejeunea trichomanis*** (Gottsche) Steph. – **GG** [Hong 1966 as *Cololejeunea goebelii*, Choe 1980, 1983 as *Cololejeunea goebelii*], **Korea** [Choe and Choi 1980 as *Cololejeunea goebelii*, Hong 1997, 2003 as *Cololejeunea goegelii*]

***Conocephalum* Hill** (Conocephalaceae)

***Conocephalum japonicum*** (Thunb.) Grolle – **JJ** [Jeju-si, Gwangryeongcheon stream, 4 May 2012, *Choi 120425c* (JNU); Horikawa 1935a as *Conocephalum supradecompositum*, Hattori et al. 1962a as *Conocephalum supradecompositum*, Choe 1975 as *Conocephalum supradecompositum*, Song and Yamada 2006], **JB** [Namwon-si, Mt. Jiri, Simwon valley, 20 Jun 2009, *Choi 3971* (JNU)], **GN** [Hong 1962a as *Conocephalum supradecompositum*], **GB** [Cheongsong-gun, Mt. Juwang, Jeolgol, 9 Nov 2010, *Choi 8923* (JNU); Hong 1962c as *Conocephalum supradecompositum*], **CN** [Choe 1980 as *Conocephalum supradecompositum*], **GG** [Hong 1962a as *Conocephalum supradecompositum*], **GW** [Jeongseon-gun, River Donggnag, Limstone, 7 Sep 2011, *Choi 110900* (JNU); Hong 1962a as *Conocephalum supradecompositum*, Kim et al. 1995, Choe 1975, 1980 as *Conocephalum supradecompositum*], **PN** [Gao and Chang 1983a, b as *Conocephalum supradecompositum*.], **PB** [Kim and Hwang 1991 as *Conocephalum supradecompositum*], **JG** [Kim and Hwang 1991 as *Conocephalum supradecompositum*], **HB** [Kim and Hwang 1991 as *Conocephalum supradecompositum*], **Korea** [Horikawa 1939 as *Conocephalum supradecompositum*, Iwatsuki and Mizutani 1972 as *Conocephalum supradecompositum*, Inoue 1974 as *Conocephalum supradecompositum*, Choe and Yamada 1974 as *Conocephalum supradecompositum*, Choe and Choi 1980 as *Conocephalum supradecompositum*, Gao and Chang 1981 as *Conocephalum supradecompositum*, Aur and Zhang 1985 as *Conocephalum supradecompositum*, Li 1985 as *Conocephalum supradecompositum*, Long and Grolle 1990; Hong 1997, 2003]

***Conocephalum salebrosum*** Szweyk., Buczk. et Odrzyk. – **JJ** [Jeju-si, Gwangryeongcheon stream, 4 May 2012, *Choi 120461* (JNU); Horikawa 1935a as *Conocephalum conicum*, Hattori et al. 1962a as *Conocephalum conicum*, Hong 1962a as *Conocephalum conicum*, Song and Yamada 2006 as *Conocephalum conicum*], **JN** [Gurye-gun, Mt. Jiri, Nogodan, 19 Sep 2009, *Choi 5044* (JNU)], **JB** [Buan-gun, Mt. Naebyen, Beadrock near road, 10 Mar 2009, *Choi 3376* (JNU)], **GN** [Hamyang-gun, Mt. Jiri, Chilseon valley, 28 Sep 2010, *Choi 8124* (JNU); Song and Yamada 2009a as *Conocephalum salebrosum*], **GB** [Cheongsong-gun, Mt. Juwang, Jeolgol, 9 Nov 2010, *Choi 8921* (JNU); Horikawa 1955], **CN** [Choe 1979 as *Conocephalum conicum*], **GG** [Hong and Kim 1961a as *Conocephalum conicum*, Hong 1962a as *Conocephalum conicum*], **GW** [Hwancheon-gun, *Cypripedium japonicum* area, 24 Jul 2009, *Choi 4145*; Hong 1962a as *Conocephalum conicum*, Kim and Hwang 1991 as *Conocephalum conicum*, Kim et al. 1995 as *Conocephalum conicum*], **HWN** [Kim and Hwang 1991 as *Conocephalum conicum*], **PB** [Kim and Hwang 1991 as *Conocephalum conicum*], **PN** [Horikawa 1935a as *Conocephalum conicum*, Gao and Chang 1983a, b as *Conocephalum conicum*], **JG** [Kim and Hwang 1991 as *Conocephalum conicum*], **YG** [Kim and Hwang 1991 as *Conocephalum conicum*], **HN** [Kim and Hwang 1991 as *Conocephalum conicum*], **HB** [Kim and Hwang 1991 as *Conocephalum conicum*], **Korea** [Horikawa 1934a, 1939 as *Conocephalum conicum*, Hattori 1971 as *Conocephalum conicum*, Choe and Yamada 1974 as *Conocephalum conicum*, Choe and Choi 1980 as *Conocephalum conicum*, Gao and Chang 1981 as *Conocephalum conicum*, Hong 1997, 2003 as *Conocephalum conicum*]

***Cryptolophocolea* L. Söderstr., Crand.-Stotl., Stotler et Váňa** (Lophocoleaceae)

°***Cryptolophocolea compacta*** (Mitt.) L. Söderstr. – **JN** [Hong 1966 as *Lophocolea compacta*, Song and Yamada 2009b as *Lophocolea compacta*], **JB** [Hong 1966 as *Lophocolea compacta*], **GN** [Hattori et al. 1962b as *Lophocolea compacta*], **GB** [Hong 1966 as *Lophocolea compacta*], **GG** [Hong 1966 as *Lophocolea compacta*], **GW** [Hong 1962a, 1962b, 1966 as *Lophocolea compacta*], **YG** [Kim and Hwang 1991 as *Lophocolea compacta*], **HN** [Kim and Hwang 1991 as *Lophocolea compacta*], **HB** [Kim and Hwang 1991 as *Lophocolea compacta*], **Korea** [Choe and Yamada 1974 as *Lophocolea compacta*, Inoue 1974 as *Lophocolea compacta*, Choe and Choi 1980 as *Lophocolea compacta*, Hong 1997, 2003 as *Lophocolea compacta*]

***Cylindrocolea* R.M.Schust.** (Cephaloziellaceae)

*Cylindrocolea kiaeri (Austin) Váňa – **GN** [Sancheong-gun, Sicheon-myeon, Mt. Jiri, Jungsanri valley, 10 Nov 2019, *Choi 1910844a* (JNU)], **CB** [Boeun-gun, Mt. Sokri, Cheonwhangbong area, 18 Oct 2019, *Choi 1910568* (JNU)]

***Cylindrocolea recurvifolia*** (Steph.) Inoue – **JJ** [Seogwipo-si, Hyodon stream, 7 Aug 2010, *Choi 7663* (JNU); Kitagawa 1965a as *Cephaloziella revurvifolia*, Song and Yamada 2006], **JN** [Goangyang-si, Mt. Baekun, 1 Aug 2009, *Choi 4200* (JNU); Hong 1962a as *Cephaloziella revurvifolia*], **JB** [Buan-gun, Mt. Naebyen, Jikso Waterfall, 10 Mar 2009, *Choi 330* (JNU)*4*], **GN** [Hapcheon-gun, Mt, Gaya, Sangwangbong, 8 Sep 2009, *Choi 4366* (JNU); Hong 1962a], **CN** [Gongju-si, Mt. Gyeryong, Temple Donghaksa valley, 8 Jul 2009, *Choi 4094* (JNU)], **GG** [Hong 1960c,1962a, 1966 as *Cephaloziella revurvifolia*, Choe 1980], **GW** [Sokcho-si, Mt. Seolak, Biseondae, 11 Oct 2010, *Choi 8344* (JNU); Hong 1962a, 1966 as *Cephaloziella revurvifolia*, Kitagawa 1965a as *Cephaloziella revurvifolia*, Kim and Hwang 1991; Kim et al. 1995], **Korea** [Iwatsuki and Mizutani 1972; Inoue 1974; Choe and Yamada 1974; Choe and Choi 1980; Hong 1997, 2003]

***Diplophyllum* (Dumort.) Dumort.** (Scapaniaceae)

***Diplophyllum albicans*** (L.) Dumort. – **JJ** [Jeju-si, Mt. Halla, Baekrokdam, 8 Aug 2010, *Choi 7761* (JNU)], **JN** [Gurye-gun, Mt. Jiri, Nogodan, 29 Apr 2009, *Choi* 3521 (JNU); Hong 1962a, 1966; Choe 1983; Song and Yamada 2009b], **JB** [Jeongeup-si, Mt. Naejang, Geumseon valley, 16 Mar 2009, *Choi 3494* (JNU)], **GN** [Geochang-gun, Mt. Namdeogyu, top of mountain, 11 Nov 2010, *Choi 8975* (JNU); Hattori et al. 1962b; Choe 1980, 1983], **GW** [Inje-gun, Mt. Seolak, Bongjeongam, 14 Oct 2010, *Choi 8585* (JNU); Kim et al. 1995], **PN** [Gao and Chang 1983b], **PB** [Kim and Hwang 1991], **YG** [Kim and Hwang 1991], **HN** [Gao and Chang 1983a], **Korea** [Choe and Yamada 1974; Schuster 1974; Choe and Choi 1980; Hong 1997, 2003]

***Diplophyllum andrewsii*** A.Evans – **JN** [Gurye-gun, Mt. Jiri, 29 Apr 2009, *Choi 3521* (JNU)], **GW** [Jeongseon-gun, Mt. Seokbyeong, 30 Sep 2009, *Choi 5280* (JNU); Kim et al. 1995], **HN** [Kim and Hwang 1991], **HB** [Kim and Hwang 1991], **Korea** [Hong 1997, 2003]

***Diplophyllum serrulatum*** (Müll.Frib.) Steph. – **JJ** [Horikawa 1934b; Horikawa 1939; Hattori 1944a; Amakawa and Hattori 1955; Hong 1966; Choe 1980; Song and Yamada 2006], **JB** [Buan-gun, Mt. Naebyen, Beadrock near road, 10 Mar 2009, *Choi 3363* (JNU); Hong 1966, Choe 1980], **GN** [Namhae-gun, Mt. Geum, valley, 21 May 2011, *Choi 110629* (JNU)], GB [Hong 1966, Choe 1980], **CN** [Choe 1980], **GG** [Hong 1966; Choe 1980], **GW** [Inje-gun, Mt. Seolak, Bongjeongam, 14 Oct 2010, *Choi 8620* (JNU)], **HB** [Kim and Hwang 1991], **Korea** [Hong 1962a; Iwatsuki and Mizutani 1972; Choe and Yamada 1974; Choe and Choi 1980; Hong 1997, 2003]

°***Diplophyllum sibiricum*** Vilnet et Bakalin – **GW** [Kim and Hwang 1991 as *Diplophyllum obtusifolium*], **PN** [Kim and Hwang 1991 as *Diplophyllum obtusifolium*], **YG** [Kim and Hwang 1991 as *Diplophyllum obtusifolium*], **HN** [Kim and Hwang 1991 as *Diplophyllum obtusifolium*], **HB** [Kim and Hwang 1991 as *Diplophyllum obtusifolium*]

***Diplophyllum taxifolium*** (Wahlenb.) Dumort. – **JJ** [Jeju-si, Tamla valley, 10 Apr 2012, *Choi 120295* (JNU); Horikawa 1934c, 1936a; Amakawa and Hattori 1955], **JB** [Jinan-gun, Unilam-Banilam stream, 22 Jul 2009, *Choi 4132* (JNU)], **GN** [Hamyang-gun, Mt. Jiri, Cheonwnagbong, 28 Sep 2010, *Choi 9126* (JNU)], **GB** [Ulleung-gun, Mireukbong, 21 Oct 2010, *Choi 8793* (JNU); Hong 1962a, c, 1966; Choe 1980], **GG** [Hong 1966; Choe 1980], **GW** [Inje-gun, Mt. Seolak, Socheong shelter valley, 11 May 2011, *Choi 110233* (JNU); Gao and Chang 1983a, b; Kim et al. 1995], **PB** [Kim and Hwang 1991], **JG** [Kim and Hwang 1991], **YG** [Kim and Hwang 1991], **HN** [Kim and Hwang 1991], **HB** [Gao and Chang 1983a, b; Kim and Hwang 1991], **Korea** [Iwatsuki and Mizutani 1972; Inoue 1974; Choe and Yamada 1974; Choe and Choi 1980; Gao and Chang 1981; Aur and Zhang 1985; Hong 1997, 2003]

***Douinia* (C.E.O.Jensen) H.Buch** (Scapaniaceae)

***Douinia plicata*** (Lindb.) Konstant. et Vilnet – **JJ** [Seogwipo-si, Mt. Halla 21 Jun 2011, *Choi 111095* (JNU)], **GN** [Hamyang-gun, Mt. Jiri, Chilseon valley, 28 Sep 2010, *Choi 8173* (JNU); Choe 1980 as *Diplophyllum plicatum*], **GB** [Ulleung-gun, Seonginbong, 20 Oct 2010, *Choi 8765* (JNU)], **GW** [Inje-gun, Mt. Seolak, Baekdamsam Temple valley, 11 May 2011, *Choi 110147* (JNU); Horikawa 1936d as *Diplophyllum plcatum*, Song 1987 as *Macrodiplophyllum plicatum*, Choe 1980 as *Diplophyllum plicatum*, Kim et al. 1995 as *Diplophyllum plcatum*], **PN** [Gao and Chang 1983a, 1983b as *Macrodiplophyllum plicatum*], **PB** [Kim and Hwang 1991 as *Diplophyllum plcatum*], **YG** [Kim and Hwang 1991 as *Diplophyllum plcatum*], **HN** [Kim and Hwang 1991 as *Diplophyllum plcatum*], **HB** [Kim and Hwang 1991 as *Diplophyllum plcatum*], **Korea** [Horikawa 1955 as *Diplophyllum plcatum*, Amakawa and Hattori 1955 as *Macrodiplophyllum plcatum*, Hong 1962a as *Macrodiplophyllum plicatum*, Iwatsuki and Mizutani 1972 as *Macrodiplophyllum plicatum*, Inoue 1974 as *Diplophyllum plcatum*, Choe and Yamada 1974 as *Diplophyllum plcatum*, Gao and Chang 1981 as *Macrodiplophyllum plicatum*, Choe and Choi 1980 as *Diplophyllum plicatum*, Hong 1997, 2003 as *Diplophyllum plcatum*]

***Drepanolejeunea* (Spruce) Steph.** (Lejeuneaceae)

*Drepanolejeunea angustifolia (Mitt.) Grolle – **JJ** [Jeju-si, Baekrokdam, 28 Oct 2011, *Choi 111242* (JNU)], **GN** [Hamcheon-gun, Mt. Gaya, top of mountain, 28 Apr 2009, *Choi 4371* (JNU)].

***Dumortiera* Nees** (Dumortieraceae)

***Dumortiera hirsuta*** (Sw.) Nees – **JJ** [Jeju-si, Musu stream, 28 Oct 2010, *Choi 8829* (JNU); Horikawa 1935a; Hattori et al. 1962a; Hong 1962a; Choe 1975, 1980; Miller et al. 1983; Song and Yamada 2006], **JN** [Haenam-gun, Mt. Duryun, Taehengsa Temple valley, 18 May 2011, *Choi 110458* (JNU)], **JB** [Choe 1980], **CN** [Choe 1979], **GG** [Choe 1980], **Korea** [Shin 1970; Choe and Yamada 1974; Choe and Choi 1980; Hong 1997, 2003]

***Eocalypogeia* (R.M.Schust.) R.M.Schust**. (Calypogeiaceae)

***Eocalypogeia quelpaertensis*** (S.Hatt. et Inoue) R.M.Schust. – **JJ** [Jeju-si, Musu stream, 28 Oct 2010, *Choi 8844* (JNU); Hattori et al. 1962a as *Metacalypogeia quelpaertensis*, Hong 1966 as *Metacalypogeia quelpaertensis*, Iwatsuki and Mizutani 1972 as *Metacalypogeia quelpaertensis*, Choe 1975, 1980, 1983 as *Metacalypogeia quelpaertensis*, Inoue 1986 as *Metacalypogeia quelpaertensis*, Song and Yamada 2006], **GB** [Ulleung-gun, Seonginbong, 22 Aug 2008, *Choi 20080822-6-54* (JNU)], **Korea** [Choe and Yamada 1974 as *Metacalypogeia quelpaertensis*, Choe and Choi 1980 as *Metacalypogeia quelpaertensis*, Hong 1997, 2003 as *Metacalypogeia quelpaertensis*]

***Fossombronia* Raddi** (Fossombroniaceae)

***Fossombronia japonica*** Schiffn. – **JJ** [Jeju-si, *Cryptomeria japonica* forest, 25 Aug 2010, *Choi 8005* (JNU)], **GN** [Hamcheon-gun, Mt. Hwangmae, ridge, 3 Aug 2010, *Choi 7487* (JNU)], **GB** [Ulsan-si, Mt. Jeongjok, Mujechi 1 neup, 30 Sep 2010, *Choi 8309* (JNU)], **CN** [Choe 1980, 1983 as *Fossombronia cristula*], **GW** [Yeongweol-gun, River donggang, near the Donggang, 29 Sep 2009, *Choi 5240* (JNU)], **Korea** [Choe and Choi 1980 as *Fossombronia cristula*, Hong 1997, 2003 as Fossombronia foveolata var. cristula]

°***Fossombronia pusilla*** (L.) Nees. – **GW** [Kim and Hwang 1991, Kim et al. 1995], **PN** [Gao and Chang 1983a, 1983b], **PB** [Kim and Hwang 1991], **Korea** [Hong 1997, 2003]

***Frullania* Raddi** (Frullaniaceae)

***Frullania amplicrania*** Steph. – **JN** [Sinan-gun, Is. Gageo, The 2^nd^ villiage-Lighthouse, 2 Mar 2010, *Choi 7211* (JNU)], **GB** [Choe and Yamada 2000], **Korea** [Hong 2003]

°***Frullania aoshimensis*** Horik. – **GN** [Song and Yamada 2009a]

***Frullania appendiculata*** Steph. – **JJ** [Jeju-si, Musu stream, Goangryeong 2^nd^ Bridge, 17 Mar 2012, *Choi 120087* (JNU); Horikawa 1934d*as Frullania moniliata*, Horikawa 1955, Hattori et al. 1962a as Frullania tamarisci subsp. monilata, Hong 1962a as Frullania tamarisci subsp. monilata, Song and Yamada 2006 as Frullania tamarisci subsp. obscura], **JN** [Gurye-gun, Mt. Jiri, Banyabong, 5 Aug 2010, *Choi 7564* (JNU); Uno and Takahashi 1940 as Frullania moniliata subsp. obscura, Hong 1962a, 1966 as Frullania tamarisci subsp. monilata, Song and Yamada 2009b as Frullania tamarisci subsp. obscura], **JB** [Buan-gun, Mt. Naebyen, Jikso Waterfall, 10 Mar 2009, *Choi 3307* (JNU); Hong 1966 as Frullania tamarisci subsp. monilata], **GN** [Geochang-gun, Mt. Namdeogyu, top of mountain, 11 Nov 2010, *Choi 8945* (JNU); Horikawa 1934d*as Frullania moniliata*, Hong and Yoo 1961*as Frullania moniliata*, Hattori et al. 1962b as Frullania tamarisci subsp. monilata, Hong 1962a, 1966 as Frullania tamarisci subsp. monilata, Song and Yamada 2009a as Frullania tamarisci subsp. obscura], **GB** [Cheongsong-gun, Mt. Juwang, The 2^nd^ waterfall, 8 Nov 2010, *Choi 8920* (JNU); Horikawa 1955 as Frullania tamarisci subsp. obscura, Hong 1966 as Frullania tamarisci subsp. monilata], **CN** [Horikawa 1934d*as Frullania moniliata*, Choe 1979 as *Frullania tamarisci*], **CB** [Hong 1962a, 1962b, 1966 as Frullania tamarisci subsp. monilata], **GG** [Hong 1960b as Frullania moniliata subsp. obscura, Hong and Kim 1961a as Frullania tamarisci subsp. monilata, Hong 1962a, 1966 as Frullania tamarisci subsp. monilata], **GW** [Inje-gun, Mt. Seolak, Bongjeongam valley, 11 May 2011, *Choi 110200* (JNU); Horikawa 1955; Hong and Kim 1960; Hong 1962a, 1966 as Frullania tamarisci subsp. monilata, Kim et al. 1995 as *Frullania tamarisci*], **HWN** [Kim and Hwang 1991 as *Frullania tamarisci*], **PN** [Kim and Hwang 1991 as *Frullania tamarisci*], **PB** [Kim and Hwang 1991 as *Frullania tamarisci*], **YG** [Gao and Chang 1983a, 1983b as *Frullania tamarisci*], **HN** [Kim and Hwang 1991*as Frullania moniliata*], **HB** [Gao and Chang 1983b as *Frullania tamarisci*], **Korea** [Reimers 1931 as *Frullania clavellata*, Horikawa 1934a, 1939 as *Frullania moniliata*, Hattori 1944a as Frullania tamarisci subsp. obscura, Kamimura 1961 as Frullania tamarisci subsp. monilata, Shin 1968 as Frullania tamarisci subsp. monilata, Choe and Yamada 1974 as Frullania moniliata subsp. obscura, Choe and Choi 1980 as Frullania tamarisci subsp. obscura, Gao and Chang 1981 as Frullania tamarisci subsp. obscura, Hattori and Lin 1985 as Frullania tamarisci subsp. obscura, Li 1985 as Frullania tamarisci subsp. moniliata, Aur and Zhang 1985 as Frullania tamarisci subsp. moniliata, Chao and Lin 1991 as *Frullania tamarisci*, Hong 1997, 2003 as Frullania tamarisci subsp. obscura]

°***Frullania austinii*** J.J.Atwood, Vilnet, Mamontov et Konstant. – **GW** [Kim and Hwang 1991 as *Frullania bolanderi*], HN [Kim and Hwang 1991 as *Frullania bolanderi*], **HB** [Kim and Hwang 1991 as *Frullania bolanderi*], **Korea** [Choe 1980 as *Frullania bolanderi*, Hong 1997, 2003 as *Frullania bolanderi*]

°***Frullania brotheri*** Steph. – **Korea** [Choe 1980; Choe and Choi 1980; Hong 1997, 2003]

*Frullania crispiplicata Yuzawa et S.Hatt. – **JJ** [Seogwipo-si, Bolrae Oreum, 5 Sep 2012, *Choi 120750* (JNU)], **GN** [Namhae-gun, Mt. Geum, 21 May 2011, *Choi 110646* (JNU)]

***Frullania davurica*** Hampe ex Gottsche, Lindenb. et Nees – **JJ** [Jeju-si, Musu stream, Goangryeong 2^nd^ Bridge, 17 Mar 2012, *Choi 120079* (JNU); Hong 1966, Song and Yamada 2006], **JN** [Hong 1962a, 1966 as Frullania jackii subsp. japonica, Song and Yamada 2009b], **JB** [Hong 1966 as Frullania jackii subsp. japonica], **GN** [Hong and Yoo 1961 as *Frullania japonica*, Song and Yamada 2009a], **GB** [Hong 1966 as Frullania jackii subsp. japonica], **CN** [Hong 1966 as Frullania jackii subsp. japonica], **CB** [Hong 1962a as Frullania jackii subsp. japonica, Hong 1962d as *Frullania japonica*], **GG** [Hong l960b as as *Frullania japonica*, Hong and Kim 1961a as *Frullania jackii*, Hong 1962a, 1966 as Frullania jackii subsp. japonica], **GW** [Hong 1966 as Frullania jackii subsp. japonica, Kim et al. 1995], **HWN** [Kim and Hwang 1991], **PN** [Gao and Chang 1983a, b], **PB** [Kim and Hwang 1991], **JG** [Kim and Hwang 1991], **YG** [Kim and Hwang 1991], **HN** [Kim and Hwang 1991], **HB** [Kim and Hwang 1991], **Korea** [Reimers 1931 as *Frullania jackii*, Horikawa 1934a, 1939 as *Frullania japonica*, Kamimura 1961 as Frullania jackii subsp. japonica, Iwatsuki and Mizutani 1972 as Frullania jackii subsp. japonica, Choe and Yamada 1974 as Frullania jackii subsp. japonica, Inoue 1976 as *Frullania jackii*, Inoue 1981, Choe and Choi 1980 as Frullania jackii subsp. japonica, Hattori and Lin 1985, Li 1985 as Frullania jackii subsp. japonica, Chao and Lin 1992a, b; Hong 1997, 2003]

***Frullania densiloba*** Steph. ex A.Evans – **JJ** [Shin 1970, Kamimura 1961, Choe 1980, Miller et al. 1983, Song and Yamada 2006], **JN** [Jangheung-gun, Mt. Cheongoan, valley, 19 May 2011, *Choi 110512* (JNU)], **GN** [Namhae-gun, Mt. Geum, top of mountain, 21 May 2011, *Choi 110640* (JNU); Song and Yamada 2009a], **Korea** [Shin 1968; Iwatsuki and Mizutani 1972; Inoue 1981; Hattori and Lin 1985; Chao and Lin 1991, 1992b; Choe and Yamada 1974; Choe and Choi 1980; Hong 1997, 2003]

***Frullania diversitexta*** Steph. – **JJ** [Choe 1980, Song and Yamada 2006], **JN** [Hong 1962a, 1966; Choe 1980; Song and Yamada 2009b], **JB** [Muju-gun, Mt. Deogyu, 25 Jun 2008, *Choi 10680* (JNU)], **GN** [Hong and Yoo 1961; Hattori et al. 1962b; Hong 1966; Choe1980; Song and Yamada 2009a], **GG** [Hong 1962a, 1966, Choe 1980], **Korea** [Kamimura 1961; Iwatsuki and Mizutani 1972; Hattori and Lin 1985; Choe and Yamada 1974; Choe and Choi 1980; Hong 1997, 2003]

°***Frullania ericoides*** (Nees) Mont. – **JJ** [Choe 1980; Song and Yamada 2006], **JN** [Song and Yamada 2009b], **JB** [Hong 1966 as *Frullania squarrosa*, Choe 1980], **GN** [Song and Yamada 2009a] , **CN** [Hong l962a, 1966 as *Frullania squarrosa*, Choe 1979], **GG** [Hong l966 as *Frullania squarrosa*, Choe 1980], **GW** [Hong l962a as *Frullania squarrosa*, Kim et al. 1995], **HWN** [Kim and Hwang 1991], **PN** [Kim and Hwang 1991], **PB** [Kim and Hwang 1991], **JG** [Kim and Hwang 1991], **YG** [Kim and Hwang 1991], **HN** [Kim and Hwang 1991], **HB** [Kim and Hwang 1991], **Korea** [Kamimura 1961 as *Frullania squarrosa*, Li 1985 as *Frullania squarrosa*, Choe and Yamada 1974; Choe and Choi 1980; Hong 1997, 2003]

***Frullania fauriana*** Steph. – **JJ** [Seogwipo-si, Bolrae Oreum, 5 Sep 2012, *Choi 120748* (JNU)], **JN** [Uno and Takahashi 1940, Song and Yamada 2009b], **GN** [Hong and Yoo 1961], **GB** [Hong 1966; Choe 1980, 1983], **Korea** [Hong 1962a; Shin 1968; Miller et al. 1983; Choe and Choi 1980; Hong 1997, 2003]

***Frullania fuscovirens*** Steph. – **JJ** [Jeju-si, Tamla valley, 25 Sep 2012, *Choi 120998* (JNU)], **YG** [Kim and Hwang 1991], **Korea** [Stephani 1910b; Hattori 1980 as Frullania valida var. fuscovirens, Hattori and Lin 1985; Hong 1997, 2003]

***Frullania hamatiloba*** Steph. – **JJ** [Jeju-si, Tamla valley, 10 Apr 2012, *Choi 120297* (JNU); Hong 1966; Song and Yamada 2006], **JN** [Gurye-gun, Mt. Jiri, Nododan, 5 Aug 2010, *Choi 7545* (JNU); Hong 1966, Song and Yamada 2009b], **JB** [Jangsu-gun, Mt. Waryong, Forest lodge, 10 May 2012, *Choi 120577* (JNU); Hong 1966], **GN** [Hong 1966], **GB** [Hong 1966], **GG** [Hong 1962a, 1966], **GW** [Hong 1962a, 1966], **HN** [Choe 1983 as *Frullania koreana*], **Korea** [Stephani 1911 as *Frullania koreana*, Kamimura 1961; Hattori 1980 as *Frullania koreana*, Iwatsuki and Mizutani 1972; Inoue 1981; Choe and Yamada 1974; Hong 1997 as *Frullania koreana*, Choe and Choi 1980 as *Frullania koreana*, Choe 1983 as *Frullania koreana*, Hong 2003]

***Frullania inflata*** Gottsche – **JJ** [Jeju-si, Hancheon Bridge, 14 Mar 2012, *Choi 120013* (JNU); Song and Yamada 2006], **JN** [Yesu-gun, Mt. Geumo, Yulso-ri valley, 24 Feb 2010, *Choi 7120* (JNU)], JB [Choe 1980], **GN** [Hong 1966 as *Frullania mayebarae*, Choe 1980; Song and Yamada 2009a], **CN** [Choe 1980], **GG** [Hong 1966 as *Frullania mayebarae*], Korea [Hattori and Lin 1985; Choe and Choi 1980; Hong 1997, 2003]

°***Frullania inflexa*** Mitt. – **GW** [Hong 1962a as *Frullania delavayi*], **Korea** [Hong 1997, 2003]

***Frullania kagoshimensis*** Steph. – **JJ** [Hong 1966, Song and Yamada 2006], **JN** [Gurye-gun, Mt. Jiri, Nododan, 5 Aug 2010, *Choi 7561* (JNU); Hong 1966; Song and Yamada 2009b], **JB** [Namwon-si, Mt. Jiri, Jeongryeongchi area, 26 Aug 2009, *Choi 4250* (JNU)], **GN** [Geoje-si, Oryung reservoir, 16 Mar 2011, *Choi 110035* (JNU)], **GB** [Ulsan-si, Mt. Sinbul, Danjo neup, 1 Oct 2010, *Choi 8329* (JNU); Hong 1966], **GG** [Hong 1966], **GW** [Inje-gun, Mt. Seolak, Hangyeoryeong, 21 Sep 2009, *Choi 5097* (JNU); Hong 1966], **Korea** [Choe and Choi 1980; Hong 1997, 2003]

***Frullania muscicola*** Steph. – **JJ** [Jeju-si, Hancheon Bridge, 14 Mar 2012, *Choi 120003* (JNU); Hattori et al. 1962a; Hong 1962a, 1966; Choe 1980 as Frullania muscicola var. inuena, Song and Yamada 2006], **JN** [Hwasun-gun, Psilotun nudum area, 1 Aug 2009, *Choi 4202* (JNU); Hong 1962a, 1966; Song and Yamada 2009b], **JB** [Muju-gun, Mt. Jeoksang, below Waterfall, 25 Mar 2009, *Choi 3411* (JNU); Hong 1966], **GN** [Geoje-si, Oryung reservoir, 16 Mar 2011, *Choi 110012* (JNU); Hong and Yoo 1961; Hattori et al. 1962b; Song and Yamada 2009a], **GB** [Yeongju-si, Mt. Sobaek, Birobong area, 2 Sep 2009, *Choi 4310* (JNU); Hong 1962a as Frullania muscicola var. inuena, Hong 1966], **CN** [Gongju-si, Mt. Gyeryong, Temple Donghaksa valley, 8 Jul 2009, *Choi 4122* (JNU); Choe 1972, 1979], **CB** [Hong 1962a, d], **GG** [Hong 1962a, 1966 as Frullania muscicola var. inuena, Choe 1980 as Frullania muscicola var. inuena], **GW** [Inje-gun, Mt. Seolak, Hangyeoryeong, 28 Aug 2009, *Choi 4274* (JNU); Hong 1962a, 1966; Kim et al. 1995], **HWN** [Kim and Hwang 1991], **PN** [Kim and Hwang 1991], **PB** [Kim and Hwang 1991], **JG** [Kim and Hwang 1991], **YG** [Gao and Chang 1983a, 1983b], **HN** [Kim and Hwang 1991], **HB** [Kim and Hwang 1991], **Korea** [Stephani 1910c; Kamimura 1961; Shin 1970; Iwatsuki and Mizutani 1972; Hattori 1981; Inoue 1981; Gao and Chang 1981; Hattori and Lin 1985; Aur and Zhang 1985; Li 1985; Chao and Lin 1992a; Choe and Yamada 1974; Choe and Choi 1980 as Frullania brittoniae subsp. truncatifolia, Hong 1997, 2003]

°***Frullania nepalensis*** (Spreng.) Lehm. et Lindenb. – **JJ** [Horikawa 1935a as *Frullania nishiyamensis*, Song and Yamada 2006], **Korea** [Hattori 1941 as Frullania nepalensis var. nishiyamensis, Hattori 1944a as Frullania nepalensis var. nishiyamensis, Kamimura 1961 as *Frullania nishiyamensis*, Hong 1962a as *Frullania nishiyamensis*, Choe and Yamada 1974 as *Frullania nishiyamensis*, Hattori 1975b; Choe 1980; Choe and Choi 1980; Li 1985 as Frullania*nishiyamensis*, Hong 1997, 2003]

***Frullania osumiensis*** (S.Hatt.) S.Hatt. – **JJ** [Jeju-si, Gyoraeri stream, 11 Apr 2012, *Choi 120362* (JNU); Hong 1966, Song and Yamada 2006], **JN** [Sinan-gun, Is. Gageo, Mt. Doksil, 2 Mar 2010, *Choi 7200* (JNU); Hong 1966], **GN** [Geoje-si, Oryung reservoir, 16 Mar 2011, *Choi 110010* (JNU); Hong 1966], **GB** [Hong 1966], **GW** [Hong 1966 as Frullania osumiensis var. orbiculata, Hong 1997, 2003, Choe and Choi 1980 as Frullania osumiensis var. orbiculata, Choe 1983 as Frullania osumiensis var. orbiculata]

°***Frullania parvistipula*** Steph. – **JN** [Song and Yamada 2009b], **GN** [Song and Yamada 2009a], **GB** [Choe and Yamada 2000], **HN** [Kim and Hwang 1991], **Korea** [Hong 2003]

***Frullania pedicellata*** Steph. – **JJ** [Jeju-si, Sumeunmulbyengdwi, 26 Aug 2010, *Choi 8031* (JNU)], **JN** [Goheung-gun, Is. Oenarodo, shoreline, 20 May 2011, *Choi 110600* (JNU)], **JB** [Namwon-si, Mt. Jiri, Jeongryeongchi area, 26 Aug 2009, *Choi 4208* (JNU)], **GN** [Hapcheon-gun, Mt, Gaya, Baekwondong area, 8 Sep 2009, *Choi 4342* (JNU); Hong 1962a], **CN** [Choe 1972, 1979, 1980], **CB** [Yeongdong-gun, Mt. Minjuji, Forest lodge, 19 May 2012, *Choi 120588* (JNU)], **GG** [Hong and Kim 1961a; Choe 1980], **GW** [Jeongseon-gun, River Donggang, near ther Donggang, 17 Aug 2010, *Choi 4280* (JNU); Hong 1962a], **Korea** [Choe and Yamada 1974; Choe and Choi 1980; Hong 1997, 2003]

*Frullania polyptera Taylor – **JJ** [Jeju-si, Mt. Halla, Baekrokdam, 8 Aug 2010, *Choi 7775* (JNU)], **JB** [Muju-gun, Mt. Deogyu, 27 Jun 2008, *Choi 1083* (JNU)], **GW** [Jeongseon-gun, River Donggang, near ther Donggang, 17 Aug 2010, *Choi 7933* (JNU)]

***Frullania schensiana*** C.Massal. – **JJ** [Jeju-si, Sumeunmulbyengdwi, 26 Aug 2010, *Choi 8021* (JNU); Choe 1980], **JN** [Hong 1962a, 1966; Choe 1980; Song and Yamada 2009b], **GN** [Geoje-si, Mt. Noja, Forest lodge, 17 Mar 2011, *Choi 110044* (JNU); Hong and Yoo 1961; Hattori et al. 1962b], **GB** [Ulleung-gun, Naribunji, 21 Oct 2010, *Choi 8770* (JNU)], **GW** [Hong 1962a], **Korea** [Hattori and Lin 1985; Choe and Yamada 1974; Choe and Choi 1980; Hong 1997, 2003]

***Frullania taradakensis*** Steph. – **JJ** [Jeju-si, Tamla valley, 25 Sep 2012, *Choi 120999* (JNU); Hong 1966; Song and Yamada 2006], **JN** [Hong 1962a, 1966; Song and Yamada 2009b], **JB** [Hong 1966], **GN** [Hong and Yoo 1961; Hattori et al. 1962b; Hong 1966; Choe 1980; Song and Yamada 2009a], **GB** [Hong 1962a, 1966], **CN** [Choe 1979], **CB** [Hong 1962a, d, 1966], **GG** [Hong and Kim 1961a; Hong 1962a, 1966], **GW** [Hong 1962a, 1966; Kim and Hwang 1991], **PB** [Kim and Hwang 1991], **HN** [Kim and Hwang 1991], **Korea** [Kamimura 1961; Inoue 1981; Choe and Yamada 1974; Hattori and Lin 1985; Choe and Choi 1980; Hong 1997, 2003]

***Frullania usamiensis*** Steph. – **JJ** [Jeju-si, Mt. Halla, Seongpanak-Baekrokdam, 8 Aug 2010, *Choi 7712* (JNU); Choe 1980], **JN** [Gurye-gun, Mt. Jiri, Nododan, 5 Aug 2010, *Choi 7536* (JNU); Hong 1962a, 1966, Song and Yamada 2009b], **JB** [Jangsu-gun, Mt. Waryong, Forest lodge, 10 May 2012, *Choi 120572* (JNU)], **GN** [Hamcheon-gun, Mt. Hwangmae, ridge, 3 Aug 2010, *Choi 7508* (JNU); Hong and Yoo 1961; Hattori et al. 1962b; Hong 1962a; Choe 1980; Song and Yamada 2009a], **GB** [Cheongsong-gun, Mt. Juwang, Jabang waterfall, 8 Nov 2010, *Choi 8907* (JNU)], **CB** [Yeongdong-gun, Mt. Minjuji, Forest lodge, 19 May 2012, *Choi 120590* (JNU)], **GW** [Inje-gun, Mt. Seolak, Hangyeoryeong, 28 Aug 2009, *Choi 4285* (JNU); Hong 1962a, 1966; Choe 1980; Kim and Hwang 1991], **PB** [Kim and Hwang 1991], **JG** [Kim and Hwang 1991], **HN** [Kim and Hwang 1991], **Korea** [Kamimura 1961; Inoue 1981; Choe and Yamada 1974; Choe and Choi 1980; Hong 1997, 2003]

***Fuscocephaloziopsis* Fulford** (Cephaloziaceae)

°**Fuscocephaloziopsis catenulata subsp. catenulata** (Huebener) Váňa et L.Söderstr. – **PB** [Kim and Hwang 1991 as *Cephalozia catenulata*], **YG** [Gao and Chang 1983a, 1983b as *Cephalozia catenulata*], **HN** [Kim and Hwang 1991 as *Cephalozia catenulata*], **HB** [Kim and Hwang 1991 as *Cephalozia catenulata*]

**Fuscocephaloziopsis catenulata subsp. nipponica** (S.Hatt.) Váňa et L.Söderstr. – **GN** [Hamyang-gun, Mt. Jiri, Hansin valley, 21 May 2010, *Choi 7380* (JNU), Geoje-si, Oryung reservoir, 16 Mar 2011, *Choi 110108* (JNU)], **GW** [Inje-gun, Mt. Seolak, Bongjeongam, 14 Oct 2010, *Choi 8584* (JNU); Kim and Hwang 1991 as *Cephalozia nipponica*, Kim et al. 1995 as *Cephalozia nipponica*], **PB** [Kim and Hwang as *Cephalozia nipponica*], **YG** [Kim and Hwang as *Cephalozia nipponica*], **HN** [Kim and Hwang 1991 as *Cephalozia nipponica*], **HB** [Kim and Hwang 1991 as *Cephalozia nipponica*], **Korea** [Hong 1997, 2003 as Cephalozia catenulata subsp. nipponica]

***Fuscocephaloziopsis leucantha*** (Spruce) Váňa et L.Söderstr. – **GN** [Hamyang-gun, Mt. Jiri, Cheonwnagbong, 29 Sep 2010, *Choi 8260* (JNU); Choe 1980], **CN** [Choe 1980 as *Cephalozia leucantha*], **GW** [Inje-gun, Mt. Seolak, Bongjeongam, 14 Oct 2010, *Choi 8598* (JNU); Kim and Hwang 1991 as *Cephalozia leucantha*, Kim et al. 1995 as *Cephalozia leucantha*], **JG** [Kim and Hwang 1991 as *Cephalozia leucantha*], **YG** [Kim and Hwang 1991 as *Cephalozia leucantha*], **HB** [Kim and Hwang 1991 as *Cephalozia leucantha*], **Korea** [Choe and Choi 1980 as *Cephalozia leucantha*, Hong 1997, 2003 as *Cephalozia leucantha*]

***Fuscocephaloziopsis lunulifolia*** (Dumort.) Váňa et L.Söderstr. – **JJ** [Jeju-si, Mt. Halla, Seongpanak-Baekrokdam, 8 Aug 2010, *Choi 7722* (JNU)], **GN** [Hamyang-gun, Mt. Jiri, Chilseon valley, 28 Sep 2010, *Choi 8188* (JNU)], **GB** [Ulleung-gun, Seonginbong, 20 Oct 2010, *Choi 8748* (JNU)], **GW** [Inje-gun, Mt. Seolak, Hangyeoryeong, 28 Aug 2009, *Choi 4260* (JNU); Kim et al. 1995 as *Cephalozia lunulifolia*], **JG** [Kim and Hwang 1991 as *Cephalozia lunulifolia*], **YG** [Kim and Hwang 1991 as *Cephalozia lunulifolia*], **HN** [Kim and Hwang 1991 as *Cephalozia lunulifolia*], **Korea** [Hong 1997, 2003 as *Cephalozia lunulifolia*]

***Geocalyx* Nees** (Geocalycaceae)

***Geocalyx lancistipulus*** (Steph.) S.Hatt. – **JJ** [Jeju-si, Musu stream, Goangryeong 2^nd^ Bridge, 18 Mar 2012, *Choi 120157* (JNU)], **GB** [Ulleung-gun, Seonginbong, 20 Oct 2010, *Choi 8741* (JNU)], **PN** [Kim and Hwang 1991], **Korea** [Hong 1997, 2003]

***Gymnomitrion* Corda** (Gymnomitriaceae)

***Gymnomitrion commutatum*** (Limpr.) Schiffn. – **JJ** [Seogwipo-si, Baekrokdam, Northwestern wall, 7 Sep 2012, *Choi 120924* (JNU)]

***Gymnomitrion faurianum*** (Steph.) Horik. – **JJ** [Jeju-si, Mt. Halla, Baekrokdam, 8 Aug 2010, *Choi 7759* (JNU)], **GW** [Sokcho-si, Mt. Seolak, Jungcheong, 21 Sep 2009, *Choi 5148* (JNU)]

*Gymnomitrion noguchianum S.Hatt. – **JJ** [Seogwipo-si, Mt. Halla, Baekrokdam, 6 Sep 2012, *Choi 120819* (JNU)]

***Gymnomitrion parvitextum*** (Steph.) Mamontov, Konstant. et Potemkin – **JJ** [Jeju-si, Mt. Halla, Baekrokdam, 21 Sep 2012, *Choi 120917b* (JNU)], **GN** [Sancheong-gun, Mt. Jiri, below Cheonwangbong, 15 Jun 2009, *Choi 3822* (JNU)], **GG** [Hong 1960b as *Marsupella parvietxta*, Hong 1962a, 1966 as *Marsupella commutata*, Choe 1983 as *Marsupella commutata*], **GW** [Inje-gun, Mt. Seolak, Bongjeongam valley, 11 May 2011, *Choi 110229* (JNU)], **HN** [Kim and Hwang 1991 as *Marsupella commutata*], **YG** [Gao and Chang 1983a, b as *Marsupella commutata*], **Korea** [Choe and Yamada 1974 as *Marsupella commutata*, Long and Grolle 1990 as *Marsupella commutata*, Choe and Choi 1980 as *Marsupella commutata*, Hong 1997, 2003 as *Marsupella commutata*]

***Haplomitrium* Nees** (Haplomitriaceae)

*Haplomitrium mnioides (Lindb.) R.M.Schust. – **JJ** [Jeju-si, Hyodon stream, 7 Aug 2010, *Choi 7613* (JNU)]

***Harpanthus* Nees** (Geocalycaceae)

°***Harpanthus flotovianus*** (Nees) Nees – **JN** [Choe 1983], **GN** [Choe 1980], **Korea** [Choe and Choi 1980; Hong 1997, 2003]

*Harpanthus scutatus (F.Weber et D.Mohr) Spruce – **GN** [Sancheong-gun, Mt. Jiri, Cheonwnagbong, 15 Jun 2009, *Choi 3763a* (JNU)].

***Hattoria* R.M.Schust.** (Lophoziaceae)

*Hattoria yakushimensis (Horik.) R.M.Schust. – **GN** [Hapcheon-gun, Mt, Gaya, Sangwangbong, 8 Sep 2009, *Choi 4383* (JNU)]

***Hattorianthus* R.M.Schust. et Inoue** (Moerckiaceae)

***Hattorianthus erimonus*** (Steph.) R.M.Schust. et Inoue – **JB** [Muju-gun, Mt. Deogyu, 19 Mar 2008, *Choi 10272* (JNU)], **GN** [Hamyang-gun, Mt. Jiri, Byeoksoryeong, 29 Apr 2010, *Choi 7352* (JNU)], **GW** [Kim and Hwang 1991 as *Moerckia erimona*], **PN** [Gao and Chang 1983a, 1983b as *Moerckia erimona*], JG [Kim and Hwang 1991 as *Moerckia erimona*], **HB** [Kim and Hwang 1991 as *Moerckia erimona*], Korea [Hong 2003]

***Herbertus* Gray** (Herbertaceae)

***Herbertus aduncus*** (Dicks.) Gray – **JJ** [Jeju-si, Mt. Halla, Baekrokdam, 8 Aug 2010, *Choi 7764* (JNU); Hattori 1944a as *Herbertus pusillus*; Hattori et al. 1962a as Herbertus hutchinsiae subsp. schusteri, Hong 1966 as Herbertus hutchinsiae subsp. schusteri, Song and Yamada 2006], **JN** [Sinan-gun, Is. Gageo, Mt. Doksil, 2 Mar 2010, *Choi 7190* (JNU); Song and Yamada 2009b], **GN** [Hamyang-gun, Mt. Jiri, Hansin valley, 7 Oct 2009, *Choi 6015* (JNU), Geoje-si, Mt. Noja, Forest lodge, 17 Mar 2011, *Choi 110051* (JNU); Hattori et al. 1962b as Herbertus hutchinsiae subsp. schusteri, Hong 1962a, 1966 as Herbertus hutchinsiae subsp. schusteri, Song and Yamada 2009a], **GG** [Hong 1962a, 1966 as Herbertus hutchinsiae subsp. schusteri], **GW** [Inje-gun, Mt. Seolak, Socheong, 21 Sep 2009, *Choi 5197* (JNU); Kim et al. 1995], **HWN** [Kim and Hwang 1991], **PB** [Kim and Hwang 1991], **HN** [Kim and Hwang 1991], **HB** [Kim and Hwang 1991], **Korea** [Hattori 1944b as *Herbertus pusillus*, Choe and Yamada 1974; Choe and Choi 1980; Inoue 1981; Hong 1997 as *Herbertus pusillus*, Hong 2003]

*Herbertus buchii Juslén – **JJ** [Jeju-si, Mt. Halla, Baekrokdam, 8 Aug 2010, *Choi 7762* (JNU)], **JN** [Gurye-gun, Mt. Jiri, Nododan, 5 Aug 2010, *Choi 7556* (JNU)], **GB** [Cheongsong-gun, Mt. Juwang, Weoloe valley, 9 Nov 2010, *Choi 8933* (JNU)], **GW** [Inje-gun, Mt. Seolak, Geiddaegicheonbong, 15 Oct 2010, *Choi 8663* (JNU)].

***Herbertus dicranus*** (Gottsche, Lindenb. et Nees) Trevis. – **JN** [Wando-gun, Sangwhangbong, valley, 9 Feb 2010, *Choi 3076* (JNU)], **JB** [Jeongeup-si, Mt. Naejang, Geumseon valley, 16 Mar 2009, *Choi 3481* (JNU)], **GN** [Sancheong-gun, Mt. Jiri, below Cheonwangbong, 14 Jun 2009, *Choi 3728* (JNU)], **CN** [Nonsan-si, Mt. Daedun, Surak valley, 31 Mar 2009, *Choi 3420* (JNU)]

***Heteroscyphus* Schiffn.** (Lophocoleaceae)

***Heteroscyphus argutus*** (Reinw., Blume et Nees) Schiffn. – **JJ** [Jeju-si, Bijarim, 8 Jul 2012, *Choi 120710* (JNU); Inoue 1981, Song and Yamada 2006], **JN** [Wando-gun, Sangwhangbong, valley, 9 Feb 2010, *Choi 3086* (JNU)], **Korea** [Inoue 1981; Hong 1997, 2003]

***Heteroscyphus coalitus*** (Hook.) Schiffn. – **JJ** [Jeju-si, Nokkome Oreum, 28 Oct 2010, *Choi 8811* (JNU); Hattori et al. 1962a as *Heteroscyphus bescherellei*, Hong 1966 as *Heteroscyphus bescherellei*, Miller et al. 1983 as *Heteroscyphus bescherellei*, Song and Yamada 2006], **JN** [Haenam-gun, Mt. Duryun, Duryunsa Temple, 23 Apr 2010, *Choi 7313* (JNU); Hong 1966 as *Heteroscyphus bescherellei*], **JB** [Buan-gun, Mt. Naebyen, Jikso Waterfall, 10 Mar 2009, *Choi 3316* (JNU); Hong 1962a, 1966 as *Heteroscyphus bescherellei*], **GW** [Yeongweol-gun, River donggang, near the Donggang, 29 Sep 2009, *Choi 5245* (JNU); Kim and Hwang 1991 as *Heteroscyphus bescherellei*, Kim et al. 1995 as *Heteroscyphus bescherellei*], **Korea** [Choe and Yamada 1974 as *Heteroscyphus bescherellei*, Inoue 1974 as *Heteroscyphus bescherellei*, Choe and Choi 1980 as *Heteroscyphus bescherellei*, Inoue 1988 as *Heteroscyphus bescherellei*, Hong 1997, 2003]

***Heteroscyphus planus*** (Mitt.) Schiffn. – **JJ** [Jeju-si, Nokkome Oreum, 28 Oct 2010, *Choi 8805* (JNU); Hong 1962a; Song and Yamada 2006], **JN** [Goheung-gun, Is. Oenarodo, shoreline, 20 May 2011, *Choi 110601* (JNU); Hong 1966], **JB** [Buan-gun, Mt. Naebyen, Namyeochi, 10 Mar 2009, *Choi 3329* (JNU); Hong 1966], **GN** [Hamyang-gun, Mt. Jiri, Chilseon valley, 28 Sep 2010, *Choi 8148* (JNU)], **CN** [Choe 1972, Song and Yamada 2009a], **GB** [Cheongsong-gun, Mt. Juwang, Jabang waterfall, 8 Nov 2010, *Choi 8912* (JNU); Hong 1962a, 1962c], **GG** [Hong 1962a], **GW** [Jeongseon-gun, River Donggang, near ther Donggang, 16 Aug 2010, *Choi 7912* (JNU); Hong 1962a], **HB** [Kim and Hwang 1991], **Korea** [Choe and Yamada 1974; Choe and Choi 1980; Hong 1997, 2003]

***Hygrobiella* Spruce** (Hygrobiellaceae)

***Hygrobiella laxifolia*** (Hook.) Spruce – **JJ** [Jeju-si, Musu stream, 19 Mar 2012, *Choi 120175* (JNU); Hattori et al 1962a; Song and Yamada 2006], **Korea** [Iwatsuki and Mizutani 1972; Choe and Yamada 1974; Choe 1980; Choe and Choi 1980; Hong 1997, 2003]

*Hygrobiella nishimurae N.Kitag. – **JJ** [Seogwipo-si, Donnaeko valley, 14 May 2015. *Bakalin Kor-30-58-15* (VBGI)]

***Isopaches* H.Buch** (Lophoziaceae)

*Isopaches bicrenatus (Schmidel ex Hoffm.) H.Buch – **GG** [Yeoncheon-gun, Yeoncheon-eup, Dongmak-ri, algific talus slope, 27 Aug 2012, *leg.* J.S. Kim *Kim 0899* (JNU, KB)]

***Jubula* Dumort.** (Jubulaceae)

**Jubula hutchinsiae subsp. japonica** (Steph.) Horik. et Ando – **JJ** [Jeju-si, Musu stream, Goangryeong 2^nd^ Bridge, 18 Mar 2012, *Choi 120161* (JNU); Horikawa 1935a, 1936b as *Jubula japonica*, Hattori 1944a as *Jubula japonica*, Kamimura 1961 as *Jubula japonica*, Shin 1968, 1970 as *Jubula japonica*, Song and Yamada 2006], **JB** [Hong 1966 as *Jubula japonica*, Choe 1980, 1983 as *Jubula japonica*], **GN** [Hamyang-gun, Mt. Jiri, Chilseon valley, 29 Sep 2010, *Choi 8276* (JNU)], **PN** [Gao and Chang 1983a, 1983b as *Jubula japonica*], **PB** [Kim and Hwang 1991 as *Jubula japonica*], **JG** [Kim and Hwang 1991 as *Jubula japonica*], **YG** [Kim and Hwang 1991 as *Jubula japonica*], **Korea** [Mizutani 1961 as *Jubula japonica*, Iwatuski and Mizutani 1972 as *Jubula japonica*, Inoue 1974 as *Jubula japonica*, Choe and Yamada 1974 as *Jubula japonica*, Guerke 1978 as *Jubula japonica*, Choe and Choi 1980 as *Jubula japonica*, Hong 1997, 2003 as *Jubula japonica*]

*Jubula hutchinsiae subsp. javanica (Steph.) Verd. – **JJ** [Jeju-si, Seogeomeun Oreum, 21 Jun 2011, *Choi 110858* (JNU)], **JN** [Goheung-gun, Is. Oenarodo, Mt. Bongrae, valley, 20 May 2011, *Choi 110573* (JNU)], **JB** [Buan-gun, Mt. Naebyen, Jikso Waterfall, 10 Mar 2009, *Choi 3311* (JNU)], **GN** [Geoje-si, Oryung reservoir, 16 Mar 2011, *Choi 110021* (JNU)], **GW** [Yeongweol-gun, River donggang, near the Donggang, 29 Sep 2009, *Choi 5234* (JNU)]

***Jungermannia* L.** (Jungermanniaceae)

***Jungermannia atrovirens*** Dumort. – **JJ** [Jeju-si, Musu stream, 19 Mar 2012, *Choi 120192* (JNU)], **JN** [Wando-gun, Sangwhangbong, valley, 9 Feb 2010, *Choi 7001* (JNU)], **JB** [[Namwon-gun, Mt. Jiri, Forest lodge, 28 May 2011, *Choi 110665* (JNU)], **GN** [Hapcheon-gun, Mt, Gaya, Baekwondong area, 8 Sep 2009, *Choi 4353* (JNU)], **GG** [Hong 1962a, 1966 as *Jungermannia tristis*, Choe 1980, 1983 as *Jungermannia tristis*], **GW** [Samcheok-si, Mt. Deokhang, ridge, 20 Jul 2010, *Choi 7447* (JNU)], **Korea** [Váňa 1973a; Choe and Yamada 1974; Choe and Choi 1980 as *Jungermannia tristis*, Hong 1997, 2003]

*Jungermannia borealis Damsh. et Váňa – **GB** [Ulleung-gun, Bongrae waterfall 19 Oct 2010, *Choi 8702* (JNU)]

**Jungermannia exsertifolia subsp. esxertifolia** Steph. – **JB** [Namwon-si, Mt. Jiri, Baemsagol valley, 19 Jun 2009, *Choi 3900* (JNU)], **GN** [Hong 1966 as Jungermannia cordifolia subsp. excertifolia, Choe 1980 as Jungermannia cordifolia subsp. excertifolia, Song and Yamada 2009a], **GW** [Jeongseon-gun, Mt. Seokbyeong, ridge, 30 Sep 2009, *Choi 5269* (JNU); Hong 1962a, 1966 as Jungermannia cordifolia subsp. excertifolia], **Korea** [Váňa 1973a; Váňa and Inoue 1983*Jungermannia exsertifolia*]

°**Jungermannia exsertifolia subsp. cordifolia** (Dumort.) Váňa – **HWN** [Gao and Chang 1983a, 1983b as *Solenostoma cordifolium* (Dumort.) Steph., Kim and Hwang 1991 as *Jungermannia cordifolia* Hook.], **Korea** [Choe and Choi 1980 as Jungermannia cordifolia subsp. exsertifolia, Hong 1997, 2003]

*Jungermannia pumila With. – **JJ** [Seogwipo-si, Suak valley, 29 Oct 2010, *Choi 9938b* (JNU)].

***Kurzia* G.Martens** (Lepidoziaceae)

***Kurzia makinoana*** (Steph.) Grolle – **JJ** [Seogwipo-si, Baekrokdam, Northwestern wall, 7 Sep 2012, *Choi 120831* (JNU); Hattori et al. 1962a as *Microlepidozia makinoana*, Hong 1962a, 1966 as *Microlepidozia makinoana*, Choe 1975, 1980, 1983; Song and Yamada 2006], **GN** [Hamyang-gun, Mt. Jiri, Chilseon valley, 28 Sep 2010, *Choi 8125* (JNU)], **GB** [Ulleung-gun, Seonginbong, 20 Oct 2010, *Choi 8729* (JNU)], **GW** [Inje-gun, Mt. Seolak, Bongjeongam, 14 Oct 2010, *Choi 8605*; Kim and Hwang 1991; Kim et al. 1995], **Korea** [Choe and Yamada 1974; Choe and Choi 1980; Long and Grolle 1990; Hong 1997, 2003]

***Lejeunea* Lib.** (Lejeuneaceae)

***Lejeunea anisophylla*** Mont. – **JJ** [Seogwipo-si, Is. Beomseom, 21 Mar 2012, *Choi 120247* (JNU)], **JN** [Wando-gun, Is. Bogil, ridge, 16 Nov 2010, *Choi 9004* (JNU)], **GG** [Lai et al. 2007], **Korea** [Park and Choi 2007]

*Lejeunea aquatica Horik. – **JJ** [Jeju-si, Musu stream, Goangryeong 2^nd^ Bridge, 18 Mar 2012, *Choi 120168* (JNU)], **JN** [Goheung-gun, Is. Oenarodo, Mt. Bongrae, valley, 20 May 2011, *Choi 110580* (JNU)], **GN** [Geoje-si, Oryung reservoir, 16 Mar 2011, *Choi 110014* (JNU)], **GW** [Jeongseon-gun, River Donggang, near ther Donggang, 16 Aug 2010, *Choi 7921* (JNU)]

***Lejeunea compacta*** (Steph.) Steph. – **JJ** [Seogwipo-si, Mt. Halla, Witse Oreum, 2 May 2012, *Choi 120441* (JNU); Horikawa 1934c as *Euosmolejeunea auriculata*, Hattori et al. 1962a, Hong 1962a, 1966, Song and Yamada 2006], **JN** [Suncheon-si, Mt. Jogyeo, Songgoangsa Temple, 7 Dec 2010, *Choi 9097* (JNU); Hong 1962a, 1966, Song and Yamada 2009b], **JB** [Hong 1966], **GB** [Cheongsong-gun, Mt. Juwang, Jabang waterfall, 8 Nov 2010, *Choi 8910* (JNU); Hong 1962a, 1966], **GN** [Hattori et al. 1962b], **GG** [Hong 1962a, 1966], **GW** [Sokcho-si, Mt. Seolak, Kkeutcheong, 13 Oct 2010, *Choi 8534* (JNU); Hong 1962a, 1966, Kim et al. 1995], **PB** [Kim and Hwang 1991], **YG** [Kim and Hwang 1991], **HN** [Kim and Hwang 1991], **Korea** [Reimers 1931; Shin 1968; Iwatsuki and Mizutani 1972; Inoue 1976; Choe and Yamada 1974; Choe and Choi 1980; Hong 1997, 2003]

***Lejeunea discreta*** Lindenb. – **JJ** [Jeju-si, Musu stream, Goangryeong 2^nd^ Bridge, 17 Mar 2012, *Choi 120060* (JNU); Song and Yamada 2006], **JN** [Haenam-gun, Mt. Duryun, Taehengsa Temple valley, 18 May 2011, *Choi 110474* (JNU)], **GN** [Hong 1966 as *Lejeunea vaginata*, Song and Yamada 2009a], **GG** [Hong 1960c, 1962a, 1966 as *Lejeunea vaginata*, Choe 1980], **Korea** [Choe and Yamada 1974, Choe and Choi 1980, Hong 1997, 2003]

***Lejeunea flava*** (Sw.) Nees – **JJ** [Seogwipo-si, Seondol valley, 20 Jun 2011, *Choi 110819* (JNU); Hong 1966, Choe 1980, Song and Yamada 2006], **JN** [Sinan-gun, Is. Gageo, Mt. Doksil, 2 Mar 2010, *Choi 7185* (JNU)], **Korea** [Choe and Choi 1980, Hong 1997, 2003]

***Lejeunea japonica*** Mitt. – **JJ** [Jeju-si, Musu stream, 19 Mar 2012, *Choi 120180* (JNU); Mizutani 1961; Hattori et al. 1962a; Hong 1962a, 1966; Song and Yamada 2006], **JN** [Goheung-gun, Is. Seongdudo, shoreline, 20 May 2011, *Choi 110604* (JNU); Hattori et al. 1962b; Hong 1962a, 1966; Song and Yamada 2009b], **JB** [Buan-gun, Mt. Naebyen, Jikso Waterfall, 10 Mar 2009, *Choi 3314* (JNU); Hong 1966], **GN** [Geoje-si, Oryung reservoir, 16 Mar 2011, *Choi 110011* (JNU); Hattori et al. 1962b; Hong 1962a, 1966; Song and Yamada 2009a], **GB** [Cheongsong-gun, Mt. Juwang, Weoloe valley, 9 Nov 2010, *Choi 8934* (JNU); Hong 1966], **CN** [Gongju-si, Mt. Gyeryong, Temple Donghaksa valley, 8 Jul 2009, *Choi 4073* (JNU); Choe 1979], **CB** [Hong 1962a, 1962d, 1966], GG [Hong and Kim 1961a; Hong 1962a, 1966], **GW** [Inje-gun, Mt. Seolak, Seolak waterfall, 21 Sep 2009, *Choi 5223* (JNU); Hong 1962a, 1966], **Korea** [Mizutani 1961; Shin 1968; Choe and Yamada 1974, noue 1976, Choe and Choi 1980, Hong 1997, 2003]

°***Lejeunea neelgherriana*** Gottsche – **GG** [Hong 1966 as *Lejeunea claviflora*, Choe 1980, 1983 as *Lejeunea claviflora*], **GW** [Choe 1983 as *Lejeuena claviflora*], **Korea** [Hong 1997, 2003 as *Lejeunea claviflora*]

*Lejeunea otiana S.Hatt. – **JJ** [Jeju-si, Hancheon Bridge, 14 Mar 2012, *Choi 120010* (JNU)], **JN** [Sinan-gun, Is. Gageo, Mt. Doksil, 2 Mar 2010, *Choi 7199* (JNU)].

°***Lejeunea pallidevirens*** S.Hatt. – **JN** [Park and Choi 2007]

***Lejeunea parva*** (S.Hatt.) Mizut. – **JJ** [Jeju-si, Musu stream, Goangryeong 2^nd^ Bridge, 17 Mar 2012, *Choi 120057* (JNU); Hattori et al. 1962a as *Lejeunea rotundistipula*, Hong 1962a, 1966 as *Lejeunea rotundistipula*, Song and Yamada 2006], **JN** [Goheung-gun, Is. Seongdudo, shoreline, 20 May 2011, *Choi 110615* (JNU); Hong 1966 as *Lejeunea rotundistipula*], **JB** [Jinan-gun, Unilam-Banilam stream, 22 Jul 2009, *Choi 4130* (JNU); Hong 1966 as *Lejeunea rotundistipula*], **GN** [Hamyang-gun, Mt. Jiri, Chilseon valley, 28 Sep 2010, *Choi 8117* (JNU); Song and Yamada 2009a], **GB** [Cheongsong-gun, Mt. Juwang, Jabang waterfall, 8 Nov 2010, *Choi 8911* (JNU); Hong 1966 as *Lejeunea rotundistipula*], **CN** [Gongju-si, Mt. Gyeryong, Temple Donghaksa valley, 8 Jul 2009, *Choi 4072* (JNU); Choe 1972 as *Lejeunea rotundistipula*, Choe 1979], **CB** [Hong 1962a, 1962b, 1966 as *Lejeunea rotundistipula*], **GG** [Hong 1962a, 1966 as *Lejeunea rotundistipula*], **GW** [Inje-gun, Mt. Seolak, Bongjeongam valley, 11 May 2011, *Choi 110146* (JNU); Kim et al. 1995], **HWN** [Kim and Hwang 1991], **Korea** [Mizutani 1961 as *Lejeunea rotundistipula*, Choe and Choi 1980; Zhu and Wang 1992; Hong 1997, 2003]

°***Lejeunea planiloba*** A.Evans – **JJ** [Hong 1966; Choe 1980, 1983; Song and Yamada 2006], **Korea** [Choe and Choi 1980; Hong 1997, 2003]

***Lepidozia* (Dumort.) Dumort.** (Lepidoziaceae)

°***Lepidozia fauriana*** Steph. – **JJ** [Hong 1966; Choe 1980, 1983; Song and Yamada 2006], **Korea** [Choe and Choi 1980; Hong 1997, 2003]

***Lepidozia reptans*** (L.) Dumort. – **JJ** [Jeju-si, Mt. Halla, Seongpanak- Baekrokdam, 8 Aug 2010, *Choi 7734* (JNU); Hong 1966, Choe 1975, 1980], **GN** [Hamyang-gun, Mt. Jiri, Cheonwnagbong, 29 Sep 2010, *Choi 8270*], **GB** [Yeongju-si, Mt. Sobaek, Birobong area, 2 Sep 2009, *Choi 4323* (JNU); Choe 1975, 1980], **GW** [Inje-gun, Mt. Seolak, Taecheongbong, 21 Sep 2009, *Choi 5212* (JNU); Hong 1962a, 1966; Gao and Chang 1983a,b; Kim et al. 1995], **PB** [Kim and Hwang 1991], **YG** [Kim and Hwang 1991], **HN** [Kim and Hwang 1991], **HB** [Kim and Hwang 1991], **Korea** [Choe and Yamada 1974; Kitagawa 1981; Choe and Choi 1980; Hong 1997, 2003]

***Lepidozia subtransversa*** Steph. – **JJ** [Stephani 1922 as *Lepidozia coreana*, Song and Yamada 2006], **GN** [Hamyang-gun, Mt. Jiri, Hansin valley, 7 Oct 2009, *Choi 6077* (JNU), Chilseon valley, 28 Sep 2010, *Choi 8138* (JNU)], **CN** [Choe 1980], **GW** [Inje-gun, Mt. Seolak, Hangyeoryeong, 21 Sep 2009, *Choi 5065* (JNU); Hong and Kim 1960 as *Lepidozia filamentosa*, Hong 1960a, 1962a, 1966 as *Lepidozia filamentosa*, Choe 1975 as *Lepidozia filamentosa*, Choe 1980], **HB** [Kim and Hwang 1991], **Korea** [Choe and Yamada 1974; Choe and Choi 1980; Hong 1997, 2003]

***Lepidozia vitrea*** Steph. – **JJ** [Jeju-si, Musu stream, Goangryeong 2^nd^ Bridge, 18 Mar 2012, *Choi 120101* (JNU); Hattori and Mizutani 1958; Hattori et al. 1962a; Hong 1962a, 1966; Choe 1975, 1980; Song and Yamada 2006], **JN** [Haenam-gun, Mt. Duryun, Taehengsa Temple valley, 18 May 2011, *Choi 110476* (JNU); Choe 1980], **GN** [Sancheong-gun, Mt. Jiri, Cheonwangbong, 3 Oct 2011, *Choi 111066* (JNU)], **YG** [Kim and Hwang 1991], **Korea** [Shin 1968, 1970; Inoue 1974; Choe and Yamada 1974; Choe and Choi 1980; Hong 1997, 2003]

***Liochlaena* Nees** (Delavayellaceae)

***Liochlaena subulata*** (A.Evans) Schljakov – **JJ** [Jeju-si, Mt. Halla, Seongpanak- Baekrokdam, 8 Aug 2010, *Choi 7744* (JNU); Horikawa 1934b, 1935b as *Haplozia lanceolata*, Hattori 1944a; Hong 1966 as Jungermannia lanceolata subsp. stephanii, Song and Yamada 2006 as *Jungermannia subulata*], **JN** [Hong 1962a, 1966 as Jungermannia lanceolata subsp. stephanii, Song and Yamada 2009b as *Jungermannia subulata*], **JB** [Jangsu-gun, Mt. Jangan, Banghwadong valley, 30 Apr 2009, *Choi 3581* (JNU); Hong 1966 as Jungermannia lanceolata subsp. stephanii], **GN** [Hattori et al. 1962b as Jungermannia lanceolata subsp. stephanii], **GB** [Yeongju-si, Mt. Sobaek, Birobong area, 2 Sep 2009, *Choi 4295* (JNU); Hong 1962a, 1962c, 1966 as Jungermannia lanceolata subsp. stephanii], **GG** [Hong 1962a, 1966 as Jungermannia lanceolata subsp. stephanii], **GW** [Inje-gun, Mt. Seolak, Hangyeoryeong, 28 Aug 2009, *Choi 4256* (JNU); Hong 1962a, 1966 as Jungermannia lanceolata subsp. stephanii], **HWN** [Kim and Hwang 1991 as *Jungermannia lanceolata*], **PN** [Kim and Hwang 1991 as *Jungermannia lanceolata*], **JG** [Kim and Hwang 1991 as *Jungermannia lanceolata*], **YG** [Gao and Chang 1983a, b as *Jungermannia lanceolata*], **PB** [Kim and Hwang 1991 as *Jungermannia lanceolata*], **Korea** [Váňa 1969, 1973b as *Jungermannia subulata*, Choe and Yamada 1974 as *Jungermannia subulata*, Kitagawa 1978 as *Jungermannia subulata*, Choe and Choi 1980 as *Jungermannia subulata*, Miller et al. 1983 as *Jungermannia subulata*, Long and Grolle 1990 as *Jungermannia subulata*, Hong 1997, 2003 as *Jungermannia subulata*]

***Lophocolea* (Dumort.) Dumort.** (Lophocoleaceae)

***Lophocolea bidentata*** (L.) Dumort. – **JJ** [Jeju-si, Mt. Halla, Seongpanak-Baekrokdam, 8 Aug 2010, *Choi 7739* (JNU); Hattori et al. 1962a as *Lophocolea cuspidata*, Hong 1962a, 1966 as *Lophocolea cuspidata*, Choe 1980, 1983 as *Lophocolea cuspidata*, Song and Yamada 2006], **GB** [Ulleung-gun, Mireukbong, 21 Oct 2010, *Choi 8787* (JNU); Hong 1966, Choe 1980], **GW** [Inje-gun, Mt. Seolak, Socheong shelter valley, 11 May 2011, *Choi 110241* (JNU); Hong 1966, Choe 1980, Kim et al. 1995], **HN** [Kim and Hwang 1991 as *Lophocolea cuspidata*], **HB** [Kim and Hwang 1991 as *Lophocolea cuspidata*], **Korea** [Choe and Yamada 1974 as *Lophocolea cuspidata*, Choe and Choi 1980 as *Lophocolea cusidata*, Hong 1997, 2003]

***Lophocolea heterophylla*** (Schrad.) Dumort. – **JJ** [Jeju-si, Mt. Halla, Seongpanak-Baekrokdam, 8 Aug 2010, *Choi 7740* (JNU); Hattori et al. 1962a, Song and Yamada 2006], **JN** [Gurye-gun, Mt. Jiri, Nogodan, 29 Apr 2009, *Choi 3552* (JNU)], **JB** [Buan-gun, Mt. Naebyen, Beadrock near road, 10 Mar 2009, *Choi 3362*; Hong 1966], **GN** [Geochang-gun, Mt. Namdeogyu, top of mountain, 11 Nov 2010, *Choi 8985* (JNU); Hong 1962a], **GB** [Yeongju-si, Mt. Sobaek, Birobong area, 2 Sep 2009, *Choi 4326* (JNU); Hong 1962a, 1962c, 1966], **CN** [Choe 1979], **GG** [Hong 1960c], **GW** [Inje-gun, Mt. Seolak, Socheong, 21 Sep 2009, *Choi 5180* (JNU); Hong 1962a, 1966; Kim et al. 1995], **HWN** [Gao and Chang 1983a, b], **PB** [Kim and Hwang 1991], **JG** [Kim and Hwang 1991], **YG** [Kim and Hwang 1991], **HN** [Kim and Hwang 1991], **HB** [Kim and Hwang 1991], **Korea** [Choe and Yamada 1974; Gao and Chang 1981; Choe and Choi 1980; Aur and Zhang 1985; Hong 1997, 2003]

***Lophocolea horikawana*** S.Hatt. – **JJ** [Jeju-si, Mt. Halla, Baekrokdam, 21 Sep 2012, *Choi 120903* (JNU); Hong 1962a, 1966; Choe 1980, 1983; Song and Yamada 2006], **JN** [Sinan-gun, Is. Gageodo, Bolryemi seashore 22 Apr 2012, *Choi 120400* (JNU)], **JB** [Namwon-si, Mt. Jiri, Jeongryeongchi area, 26 Aug 2009, *Choi 4242* (JNU)], **GN** [Hamyang-gun, Mt. Jiri, Chilseon valley, 28 Sep 2010, *Choi 8183* (JNU)], **GW** [Kim et al. 1995], **HB** [Kim and Hwang 1991], **Korea** [Choe and Choi 1980; Hong 1997, 2003]

***Lophocolea itoana*** Inoue – **JJ** [Choe 1980], **GW** [Inje-gun, Mt. Seolak, Socheong shelter valley, 11 May 2011, *Choi 110249* (JNU); Hong 1962a; Hong 1966; Choe 1980; Kim et al. 1995], **YG** [Kim and Hwang 1991], **Korea** [Choe and Choi 1980; Hong 1997, 2003]

***Lophocolea minor*** Nees – **JJ** [Jeju-si, Sumeunmulbyengdwi, 26 Aug 2010, *Choi 8028* (JNU); Horikawa 1934d; Hong and Kim 1961b; Hattori et al. 1962a; Hong 1966; Song and Yamada 2006], **JN** [Goheung-gun, Is. Seongdudo, shoreline, 20 May 2011, *Choi 110606* (JNU); Horikawa 1934d, Hong 1962a, Song and Yamada 2009b], **JB** [Hong 1966], **GN** [Hamyang-gun, Mt. Jiri, Chilseon valley, 28 Sep 2010, *Choi 8172* (JNU); Horikawa 1934d, Hong 1962a, 1966, Song and Yamada 2009a], **GB** [Ulleung-gun, Bongrye waterfall, 19 Oct 2010, *Choi 8700* (JNU); Hong 1962a, 1962c, 1966], **CN** [Gongju-si, Mt. Gyeryong, Temple Donghaksa valley, 8 Jul 2009, *Choi 4079* (JNU); Horikawa 1934d], **CB** [Hong 1962a, d, 1966], **GG** [Hong 1960b, 1960c, 1962a, 1966], **GW** [Inje-gun, Mt. Seolak, Hangyeoryeong, 28 Aug 2009, *Choi 4270* (JNU); Hong and Kim 1960, Hong 1962a, 1966; Kim et al. 1995], **HWN** [Kim and Hwang 1991], **PN** [Gao and Chang 1983a, 1983b], **PB** [Kim and Hwang 1991], **YG** [Kim and Hwang 1991], **HB** [Kim and Hwang 1991], **Korea** [Horikawa 1939; Hattori 1944a; Shin 1968, 1970; Choe and Yamada 1974; Choe and Choi 1980; Gao and Chang 1981; Aur and Zhang 1985; Hong 1997, 2003]

***Lophozia* (Dumort.) Dumort.** (Lophoziaceae)

°***Lophozia ascendens*** (Warnst.) R.M.Schust. – **HN** [Kim and Hwang 1991], **YG** [Gao and Chang 1983a, 1983b, Kim and Hwang 1991], **Korea** [Aur and Zhang 1985; Hong 1997, 2003]

***Lophozia guttulata*** (Lindb. et Arnell) A.Evans – **JJ** [Jeju-si, Mt. Halla, Seongpanak-Baekrokdam, 8 Aug 2010, *Choi 7741* (JNU); Hattori et al. 1962a as *Lophozia porphyroleuca*, Hong 1962a, 1966 as *Lophozia porphyroleuca*, Choe 1975, 1980, 1983; Song and Yamada 2006 as *Lophozia longiflora*], **JN** [Sinan-gun, Is. Gageodo, Bolryemi seashore 22 Apr 2012, *Choi 120392* (JNU)], **GN** [Hamyang-gun, Mt. Jiri, Cheonwnagbong, 29 Sep 2010, *Choi 8265* (JNU)], **GW** [Kim et al. 1995], **JG** [Kim and Hwang 1991], **YG** [Kim and Hwang 1991], **HN** [Kim and Hwang 1991], **HB** [Kim and Hwang 1991], **Korea** [Kitagawa 1965b as *Lophozia fauriana*, Schuster 1969 as *Lophozia porphyroleuca*, Iwatsuki and Mizutani 1972 as *Lophozia fauriana*, Choe and Yamada 1974; Inoue 1974, 1981; Choe and Choi 1980; Hong 1997, 2003 as *Lophozia longiflora*]

*Lophozia lantratovae Bakalin – **GW** [Sokcho-si, Mt. Seolak, 14 Oct 2010, *Choi 8613* (JNU)]

°***Lophozia silvicoloides*** N.Kitag. – **HN** [Kim and Hwang 1991], **HB** [Kim and Hwang 1991], **Korea** [Hong 1997, 2003]

***Lophoziopsis* Konstant. et Vilnet** (Lophoziaceae)

*Lophoziopsis excisa (Dicks.) Konstant. et Vilnet – **JJ** [Jeju-si, Mt. Halla, Jindalraebat, 9 Jul 2012, *Choi 120701* (JNU)], **GW** [Inje-gun, Mt. Seolak, Socheong, 21 Sep 2009, *Choi 5189* (JNU)]

***Lunularia* Adans.** (Lunulariaceae)

*Lunularia cruciata (L.) Dumort. ex Lindb. – **JJ** [Jeju-si, Hyodon stream, 30 Oct 2011, *Choi 110361b* (JNU)], this taxon is an introduced weedy species from Mediterranean

***Makinoa* Miyake** (Makinoaceae)

***Makinoa crispata*** (Steph.) Miyake – **JJ** [Jeju-si, Gwangryeongcheon stream, 4 May 2012, *Choi 120466* (JNU); Horikawa 1935b; Hong 1962a; Choe 1980; Song and Yamada 2006], **JN** [Gurye-gun, Mt. Jiri, Nogodan, 29 Apr 2009, *Choi 3552* (JNU)], **JB** [Muju-gun, Mt. Deogyu, 2 Apr 2008, *Choi 11306* (JNU)], **GN** [Hamyang-gun, Mt. Jiri, Byeoksoryeong, 29 Apr 2010, *Choi 7350* (JNU)], **GB** [Ulleung-gun, Naesujeon valley, 21 Oct 2010, *Choi 8795* (JNU)], **CN** [Choe 1980], **GW** [Gao and Chang 1983a, b; Kim and Hwang 1991], **Korea** [Horikawa 1939; Hattori 1944a; Shin 1968, 1970; Iwatsuki and Mizutani 1972; Choe and Yamada 1974; Inoue 1976, 1981; Choe and Choi 1980; Gao and Chang 1981; Hong 1997]

***Mannia* Corda** (Aytoniaceae)

*Mannia androgyna (L.) A.Evans – **GW** [Yeongwol-gun, Yeongwol-eup, Donggang River, elev. 353 m, 4 Oct 2019, *Choi 1910004* (JNU)]

*Mannia fragrans (Balb.) Frye et L.Clark – **GW** [Yeongwol-gun, Yeongwol-eup, Donggang River, elev. 353 m, 4 Oct 2019, *Choi 1910005* (JNU)]

°***Mannia gracilis*** (F.Weber) D.B.Schill et D.G.Long – **GW** [Gao and Chang 1983a, b as *Asterella ludwigii*, Kim and Hwang 1991 as *Asterella ludwigii*, Kim et al. 1995 as *Asterella gracilis*], **PN** [Kim and Hwang 1991 as *Asterella ludwigii*], **PB** [Kim and Hwang 1991 as *Asterella ludwigii*], **HB** [Kim and Hwang 1991 as *Asterella ludwigii*], **Korea** [Hong 1997, 2003 as *Asterella gracilis*]

*Mannia triandra (Scop.) Grolle – **GW** [Yeongwol-gun, Yeongwol-eup, Donggang River, elev. 337 m, 26 Apr 2015, *Bakalin Kor-20-1-15* (VBGI)]

***Marchantia* L.** (Marchantiaceae)

**Marchantia emarginata subsp. tosana** (Steph.) Bischl. – **JJ** [Seogwipo-si, Andeok valley, 15 Mar 2012, *Choi 120028* (JNU); Choe 1980, 1983 as *Marchantia tosana*, Song and Yamada 2006], **Korea** [Choe and Choi 1980 as *Marchantia tosana*, Hong 1997, 2003]

**Marchantia paleacea subsp. paleacea** Bertol. – **JJ** [Seogwipo-si, Cheonjiyeon Valley, 20 Mar 2012, *Choi 120217* (JNU)], **JN** [Choe 1975; Song and Yamada 2009b], **GW** [Hong 1962a; Gao and Chang 1983a, b; Kim and Hwang 1991], **PN** [Kim and Hwang 1991], **PB** [Kim and Hwang 1991], **Korea** [Choe and Yamada 1974; Hong 1997, 2003]

**Marchantia paleacea subsp. diptera** (Nees et Mont.) Inoue – **JJ** [Seogwipo-si, Donnaeko stream, 31 Oct 2011, *Choi 111362* (JNU)], **JN** [Gurye-gun, Mt. Jiri, Nogodan, 19 Sep 2009, *Choi 5035* (JNU)], **CN** [Choe 1972 as *Marchantia diptera*, Choe 1975, 1979], **GB** [Cheongsong-gun, Mt. Juwang, Jabang waterfall, 8 Nov 2010, *Choi 8902* (JNU)], **GW** [Jeongseon-gun, Mt. Seokbyeong, ridge, 30 Sep 2009, *Choi 5267* (JNU); Hong and Kim 1960 as *Marchantia diptera*, Hong 1962a as *Marchantia diptera*, Choe 1975, 1980, 1983], **Korea** [Shin 1968 as *Marchantia diptera*, Choe and Yamada 1974 as *Marchantia diptera*, Choe and Choi 1980; Hong 1997, 2003]

**Marchantia polymorpha subsp. polymorpha** L.– **GW** [Hongcheon-gun, Mt. Odae, Jilmaeneap (swamp), 5 Jul 2019, *Choi 197020* (JNU)]

*Marchantia polymorpha subsp. montivagans Bischl. et Boissel.-Dub. – **JB** [Muju-gun, Mt. Deogyu, 24 Jun 2008, *Choi 10671* (JNU)]

**Marchantia polymorpha subsp. ruderalis** Bischl. et Boissel.-Dub. – **JJ** [Jeju-si, Musu stream, 19 Mar 2012, Choi 120199 (JNU)], **JN** [Gurye-gun, Mt. Jiri, Nogodan, 19 Sep 2009, Choi 5041 (JNU); Song and Yamada 2009b], **GB** [Ulleung-gun, Mireukbong, 21 Oct 2010, Choi 8794 (JNU)], **CN** [Choe 1979], **GG** [Hong and Kim 1961a; Hong 1962a], **GW** [Inje-gun, Mt. Seolak, Jungcheong, 21 Sep 2009, Choi 5136 (JNU); Gao and Chang 1983a, b; Kim et al. 1995], **PN** [Gao and Chang 1983a, b], **All the provinces** [Kim and Hwang 1991; Choe 1980], **Korea** [Horikawa 1939; Hattori 1944a; Choe and Yamada 1974; Choe and Choi 1980; Hong 1997, 2003]

***Marsupella* Dumort.** (Gymnomitriaceae)

***Marsupella apertifolia*** Steph. – **JJ** [Jeju-si, Mt. Halla, Baekrokdam, 21 Sep 2012, *Choi 120904* (JNU)], **JN** [Wando-gun, Sangwhangbong, valley, 9 Feb 2010, *Choi 3064* (JNU)], **JB** [Muju-gun, Mt. Deogyu, 22 Nov 2008, *Choi site2-35* (JNU)], **GN** [Sancheong-gun, Mt. Jiri, below Cheonwangbong, 14 Jun 2009, *Choi 3745* (JNU)], **CN** [Nonsan-si, Mt. Daedun, 31 Mar 2009, *Choi 3405* (JNU)], **GW** [Inje-gun, Mt. Seolak, 21 Sep 2009, *Choi 5174* (JNU)]

*Marsupella koreana Bakalin et Fedosov – **JJ** [Jeju-do, Mt. Halla, *Choi 111147*], **JN** [Haenam-gun, Mt. Dureun, 5 Feb 2009, *Choi 3058* (JNU)], **JB** [Muju-gun Mt. Deogyu, 22 May 2008, *Choi 509* (JNU)], **GN** [Sancheong-gun, Mt. Jiri, 7 May 2015, *Bakalin Kor-28-4-15* (VBGI)], **CN** [Nonsan-si, Mt. Daedun, 31 Mar 2009, *Choi 3407* (JNU)]

***Marsupella pseudofunckii*** S.Hatt. – **JJ** [Jeju-si, Mt. Halla, Baekrokdam, 8 Aug 2010, *Choi 7757* (JNU); Hattori et al. 1962a; Kitagawa 1963; Hong 1966; Choe 1980; Song and Yamada 2006], **JN** [Wando-gun, Sangwhangbong, valley, 9 Feb 2010, *Choi 3072* (JNU); Hong 1962a, 1966; Song and Yamada 2009b], **JB** [Jeongeup-si, Mt. Naejang, Geumseon valley, 6 Mar 2009, *Choi 3482* (JNU)], **GN** [Geochang-gun, Mt. Namdeogyu, top of mountain, 11 Nov 2010, *Choi 8953* (JNU); Hattori et al. 1962b], **GB** [Ulleung-gun, Seonginbong, 20 Oct 2010, *Choi 8731* (JNU); Hong 1962a, 1966, Choe 1980], **GG** [Hong 1962a, 1966, Choe 1980], **GW** [Inje-gun, Mt. Seolak, Hangyeoryeong, 28 Aug 2009, *Choi 4257* (JNU); Kim et al. 1995], **HN** [Kim and Hwang 1991], **Korea** [Choe and Yamada 1974; Choe and Choi 1980; Hong 1997, 2003]

***Marsupella tubulosa*** Steph. – **JJ** [Jeju-si, Musu stream, Goangryeong 2^nd^ Bridge, 17 Mar 2012, *Choi 120075a* (JNU); Hattori et al. 1962a; Hong 1962a, 1966 as Marsupella emarginata subsp. tubulosa, Song and Yamada 2006Marsupella emarginata subsp. tubulosa], **JN** [Gurye-gun, Mt. Jiri, Nogodan, 19 Sep 2009, *Choi 5043* (JNU); Hong 1962a, 1966Marsupella emarginata subsp. tubulosa], **JB** [Muju-gun, Mt. Jeoksang, below Waterfall, 19 Mar 2009, *Choi 3407* (JNU)], **GN** [Geochang-gun, Mt. Namdeogyu, top of mountain, 11 Nov 2010, *Choi 8972* (JNU); Hattori et al. 1962b; Kitagawa 1963 as Marsupella emarginata subsp. tubulosa], **GB** [Ulleung-gun, Seonginbong, 20 Oct 2010, *Choi 8712* (JNU); Hong 1962a, 1966Marsupella emarginata subsp. tubulosa], **CN** [Choe 1979 as *Marsupella emarginata*], **CB** [Hong 1962d], **GG** [Hong 1962a, 1966 as Marsupella emarginata subsp. tubulosa], **GW** [Inje-gun, Mt. Seolak, Socheong, 21 Sep 2009, *Choi 5174* (JNU); Kim et al. 1995 as *Marsupella emarginata*], **PB** [Kim and Hwang 1991 as *Marsupella emarginata*], **HN** [Kim and Hwang 1991 as *Marsupella emarginata*], **HB** [Kim and Hwang 1991 as *Marsupella emarginata*], **Korea** [Iwatsuki and Mizutani 1972 as Marsupella emarginata subsp. tubulosa, Choe and Yamada 1974 as Marsupella emarginata subsp. tubulosa, Hattori 1975a as *Marsupella emarginata*, Choe and Choi 1980 as Marsupella emarginata subsp. tubulosa, Kitagawa 1981 as *Marsupella emarginata*, Hong 1997, 2003 as *Marsupella emarginata*]

*Marsupella vermiformis (R.M.Schust.) Bakalin et Fedosov – **JJ** [Seogwipo-si, Mt. Halla, elev. 1861 m, 21 Sep 2012, *Choi 120911* (JNU, VBGI)]

***Marsupella yakushimensis*** (Horik.) S.Hatt. – **JJ** [Jeju-si, Seogeomeun Oreum, 20 Sep 2011, *Choi 111041* (JNU)], **JN** [Hong 1962a; Choe 1980, 1983; Song and Yamada 2009b], **JB** [Namwon-si, Mt. Jiri, Jeongryeongchi area, 26 Aug 2009, *Choi 4213* (JNU)], **GN** [Hamyang-gun, Mt. Jiri, Chilseon valley, 28 Sep 2010, *Choi 8166* (JNU)], **GW** [Inje-gun, Mt. Seolak, Jungcheongbong stony field, 12 May 2011, *Choi 110295* (JNU); Kim et al. 1995], **YG** [Kim and Hwang 1991], **Korea** [Choe and Yamada 1974; Choe and Choi 1980; Hong 1997, 2003]

***Mesoptychia* (Lindb.) A.Evans** (Jungermanniaceae)

°***Mesoptychia heterocolpos*** (Thed. ex Hartm.) L.Söderstr. et Váňa – **HB** [Gao and Chang 1983a, b as *Leicolea heterocolpos*, Kim and Hwang 1991 as *Leicolea heterocolpos*], **Korea** [Aur and Zhang 1985 as *Lophozia heterocolpos*, Hong 1997 as *Lophozia heterocolpos*, Hong 2003 as *Leicolea heterocolpos*]

***Mesoptychia mayebarae*** (S.Hatt.) L. Söderstr. et Váňa – **GW** [Jeongseon-gun, River Donggnag, Limstone, 7 Sep 2011, *Choi 110898b* (JNU)], **PB** [Kim and Hwang 1991 as *Lophizia mayebarae*], **Korea** [Hong 1997 as *Lophozia mayebarae*, Hong 2003 as *Leiocolea mayebarae*]

***Metacalypogeia* (S.Hatt.) Inoue** (Calypogeiaceae)

***Metacalypogeia cordifolia*** (Steph.) Inoue – **JJ** [Jeju-si, Mt. Halla, Seongpanak- Baekrokdam, 8 Aug 2010, *Choi 7713* (JNU)], **JN** [Suncheon-si, Mt. Jogyeo, Seonamsa Temple, 7 Dec 2010, *Choi 9105* (JNU)], **JB** [Namwon-si, Mt. Jiri, Simwon valley, 20 Jun 2009, *Choi 3960* (JNU)], **GN** [Hamyang-gun, Mt. Jiri, Chilseon valley, 28 Sep 2010, *Choi 8109* (JNU)], **GB** [Ulleung-gun, Mireukbong, 21 Oct 2010, *Choi 8781* (JNU)], **GW** [Inje-gun, Mt. Seolak, Kkeucheong, 21 Sep 2009, *Choi 5112* (JNU); Hong 1962a, 1966; Choe 1975, 1980], **Korea** [Choe and Yamada 1974; Aur and Zhang 1985; Choe and Choi 1980; Hong 1997, 2003]

***Metasolenostoma* Bakalin et Vilnet** (Solenostomataceae)

*Metasolenostoma ochotense Vilnet et Bakalin – **JJ** [Seogwipo-si, Hannam Experimental forest, 476 m, *Choi 111730* (JNU)], **JN** [Jeollanam-do, Heuksando, *Choi site2-3* (JNU)] **JB** [Jeollabuk-do, Mt. Deogyu, *Choi 633* (JNU)]

***Metzgeria* Raddi** (Metzgeriaceae)

***Metzgeria furcata*** (L.) Corda – **JJ** [Choe 1975, 1980, 1983 as *Metzgeria decipiens* and *Metzgeria fauriana*, Song and Yamada 2006 as *Metzgeria decipiens*], **JN** [Haenam-gun, Mt. Duryun, Duryunsa Temple, 23 Apr 2010, *Choi 7301* (JNU); Choe 1983, Song and Yamada 2009b], **JB** [Jeongeup-si, Mt. Naejang, Meokbaengigol valley, 28 Jun 2010, *Choi 7441* (JNU)], **GN** [Hamyang-gun, Mt. Jiri, Cheonwnagbong, 29 Sep 2010, *Choi 8259* (JNU); Hong 1962a, Choe 1975, 1980, 1983], **GB** [Ulleung-gun, Seonginbong, 20 Oct 2010, *Choi 8713* (JNU)], **CN** [Gongju-si, Mt. Gyeryong, Temple Donghaksa valley, *Choi 4076* (JNU)], **GG** [Hong 1962a as *Metzgeria quadriseriata*, Choe 1980, 1983 as *Metzgeria quadriseriata*], **GW** [Inje-gun, Mt. Seolak, Seolak waterfall, 21 Sep 2009, *Choi 5224* (JNU); Kim and Hwang 1991 as *Metzgeria fauriana*, Kim et al. 1995 as *Metzgeria decipiens*], **PB** [Kim and Hwang 1991], **PN** [Kim and Hwang 1991], **JG** [Kim and Hwang 1991], **YG** [Kim and Hwang 1991 as *Metzgeria decipiens*], **HN** [Kim and Hwang 1991], **HB** [Kim and Hwang 1991], **Korea** [Miller et al. 1983 as *Metzgeria decipiens*, Choe and Yamada 1974 as *Metzgeria decipiens*, Choe and Choi 1980 as *Metzgeria decipiens*, *Metzgeria quadriseriata* and *Metzgeria fauriana*, Hong 1997, 2003 as *Metzgeria decipiens* and *Metzgeria fauriana*]

°***Metzgeria leptoneura*** Spruce – **JJ** [Hattori et al. 1962a as *Metzgeria hamata*, Hong 1962a as *Metzgeria hamata*Choe 1975, 1983 as *Metzgeria hamata*, Song and Yamada 2006], **Korea** [Gao and Chang 1981 as *Metzgeria hamata*, Miller et al. 1983; Choe and Yamada 1974 as *Metzgeria hamata*, Choe and Choi 1980 as *Metzgeria hamata*, Li 1985 as *Metzgeria hamata*, Hong 1997, 2003]

***Metzgeria lindbergii*** Schiffn. – **JJ** [Jeju-si, Nokkome Oreum, 28 Oct 2010, *Choi 8802* (JNU); Hattori et al. 1962a, Song and Yamada 2006], **JN** [Goheung-gun, Is. Oenarodo, Mt. Bongrae, valley, 20 May 2011, *Choi 110597* (JNU); Hattori et al. 1962a as Metzgeria conjugata subsp. japonica, Hong 1962a as Metzgeria conjugata subsp. japonica, Song and Yamada 2009b], **JB** [Buan-gun, Mt. Naebyen, Jikso Waterfall, 10 Mar 2009, *Choi 3309* (JNU)], **GN** [Geoje-si, Oryung reservoir, 16 Mar 2011, *Choi 110001* (JNU); Horikawa 1934c as *Metzgeria conjugata*, Hattori et al. 1962b as Metzgeria conjugata subsp. japonica], **GB** [Hong 1962a, 1962c as Metzgeria conjugata subsp. japonica], **CN** [Gongju-si, Mt. Gyeryong, Temple Donghaksa valley, 8 Jul 2009, *Choi 4118* (JNU); Choe 1972, 1979 as Metzgeria conjugata subsp. japonica], **CB** [Hong 1962d as Metzgeria conjugata subsp. japonica], **GG** [Hong 1960b as *Metzgeria conjugata*, Hong and Kim 1961a as *Metzgeria conjugata*, Hong 1962a as Metzgeria conjugata subsp. japonica, Hong l 962b], **GW** [Sokcho-si, Mt. Seolak, Biseondae, 11 Oct 2010, *Choi 8345* (JNU); Hong 1962a, b as Metzgeria conjugata subsp. japonica, Kim et al. 1995 as *Metzgeria conjugata*], **PB** [Kim and Hwang 1991 as *Metzgeria conjugata*], **JG** [Kim and Hwang 1991 as *Metzgeria conjugata*], **YG** [Kim and Hwang 1991 as *Metzgeria conjugata*], **HN** [Kim and Hwang 1991 as *Metzgeria conjugata*], **HB** [Kim and Hwang 1991 as *Metzgeria conjugata*], **Korea** [Horikawa 1939 as *Metzgeria conjugata*, Shin 1970 as Metzgeria conjugata subsp. japonica, Iwatsuki and Mizutani 1972 as as Metzgeria conjugata subsp. japonica, Choe and Yamada 1974 as Metzgeria conjugata subsp. japonica, Hattori 1975a as Metzgeria conjugata subsp. japonica, Inoue 1976 as Metzgeria conjugata subsp. japonica, Choe and Choi 1980 as *Metzgeria conjugata* and Metzgeria conjugata subsp. japonica, Miller et al. 1983 as Metzgeria conjugata subsp. japonica, Hong 1997, 2003 as *Metzgeria conjugata*]

***Metzgeria pubescens*** (Schrank) Raddi – **JJ** [Jeju-si, Mt. Halla, Seongpanak-Baekrokdam, 8 Aug 2010, *Choi 7725* (JNU); Horikawa 1935a, Hattori et al. 1962a, Hong 1962a, Choe 1975, Song and Yamada 2006], **JN** [Song and Yamada 2009b], **JB** [Muju-gun, Mt. Deogyu, Chilyeon valley, 17 Mar 2010, *Choi 7249*], **GN** [Sancheong-gun, Mt. Jiri, Jungbong area, 3 Oct 2011, *Choi 111091* (JNU); Hattori et al. 1962b, Hong 1962a, Choe 1975], **GB** [Hong 1962c], **CN** [Choe 1979], **GW** [Inje-gun, Mt. Seolak, Bongjeongam valley, 11 May 2011, *Choi 110196* (JNU); Hong 1962a, 1962b, Gao and Chang 1983a, 1983b, Kim et al. 1995], **PB** [Kim and Hwang 1991], **JG** [Kim and Hwang 1991], **YG** [Gao and Chang 1983a, 1983b], **HN** [Kim and Hwang 1991], **HB** [Kim and Hwang 1991], **Korea** [Hattori 1944a, Kuwahara 1958, 1978, Choe and Yamada 1974 as *Apometzgeria pubescens*, Choe and Choi 1980 as *Apometzgeria pubescens*, Schuster 1992a; Hong 1997, 2003]

***Metzgeria temperata*** Kuwah. – **JJ** [Jeju-si, Musu stream, Goangryeong 2^nd^ Bridge, 17 Mar 2012, *Choi 120069* (JNU); Song and Yamada 2006], **JN** [Haenam-gun, Mt. Duryun, Taehengsa Temple valley, 18 May 2011,*Choi 110453* (JNU); Song and Yamada 2009b], **GN** [Hamyang-gun, Mt. Jiri, Chilseon valley, 28 Sep 2010, *Choi 8197* (JNU); Hattori et al. 1962b as *Metzgeria fruticulosa*], **GB** [Ulleung-gun, Seonginbong, 20 Oct 2010, *Choi 8711* (JNU); Hong 1962a, 1962c as *Metzgeria fruticulosa*, Choe 1983 as *Metzgeria fruticulosa*], **GW** [Taebaek-si, Mt. Taebaek, Janggunbong, 15 Sep 2009, *Choi 4430* (JNU)], **YG** [Kim and Hwang 1991], **JG** [Kim and Hwang 1991], **HN** [Gao and Chang 1983a], **Korea** [Shin 1970; Hong 1997, 2003]

***Microlejeunea* (Spruce) Steph.** (Lejeuneaceae)

***Microlejeunea ulicina*** (Taylor) Steph. – **JJ** [Jeju-si, Musu stream, Goangryeong 2^nd^ Bridge, 18 Mar 2012, *Choi 120134* (JNU); Choe 1980, 1983 as *Lejeunea ulicina*, Song and Yamada 2006 as *Lejeunea ulicina*], **JN** [Goheung-gun, Mt. Palyeoung, Forest lodge valley, 23 Jun 2009, *Choi 4019* (JNU)], **JB** [Musu-gun, Mt. Deogyu, Hyangjeokbong area, 10 Oct 2009, *Choi 5284* (JNU)], **GN** [Namhae-gun, Mt. Geum, top of mountain, 21 May 2011, *Choi 110641* (JNU); Song and Yamada 2009a as *Lejeunea ulicina*], **GW** [Inje-gun, Mt. Seolak, Taecheongbong, 21 Sep 2009, *Choi 5208* (JNU); Kim and Hwang 1991 as *Lejeunea ulicina*, Kim et al. 1995 as *Lejeunea ulicina*], **Korea** [Choe and Choi 1980 as *Lejeunea ulicina*, Hong 1997, 2003 as *Lejeunea ulicina*]

***Mylia* Gray** (Myliaceae)

***Mylia taylorii*** (Hook.) Gray – **GN** [Hamyang-gun, Mt. Jiri, Cheonwnagbong, 29 Sep 2010, *Choi 8233* (JNU)], **GW** [Inje-gun, Mt. Seolak, Hangyeoryeong, 21 Sep 2009, *Choi 5066* (JNU)], **YG** [Gao and Chang 1983a, b], HN [Kim and Hwang 1991], **HB** [Kim and Hwang 1991], **Korea** [Inoue 1981; Hong 1997, 2003]

*Mylia verrucosa Lindb. – **GN** [Hamyang-gun, Mt. Jiri, Hansin valley, 12 Oct 2019, *Choi 1910400* (JNU)], **GW** [Inje-gun, Mt. Seolak, Baekdamsam Temple valley, 11 May 2011, *Choi 110117* (JNU)]

***Myriocoleopsis* Schiffn.** (Lejeuneaceae)

***Myriocoleopsis minutissima*** (Sm.) R.L.Zhu, Y.Yu et Pócs – **JJ** [Jeju-si, Seogeomeun Oreum, 21 Jun 2011, *Choi 110830* (JNU); Song and Yamada 2006 as *Cololejeunea minutissima*], **JN** [Wando-gun, Is. Bogil, ridge, 16 Nov 2010, *Choi 9019*], **GW** [Yeongweol-gun, River donggang, near the Donggang, 29 Sep 2009, *Choi 5246* (JNU); Kim and Hwang 1991 as *Cololejeunea minutissima*], **Korea** [Hong 1997, 2003 as *Cololejeunea minutissima*]

***Nardia* Gray** (Gymnomitriaceae)

***Nardia assamica*** (Mitt.) Amakawa – **JJ** [Jeju-si, Mt. Halla, Seongpanak- Baekrokdam, 8 Aug 2010, *Choi 7708* (JNU); Hattori et al. 1962a as *Nardia sieboldii*, Song and Yamada 2006], **JN** [Goheung-gun, Mt. Palyeoung, ridge, 23 Jun 2009, *Choi 4034* (JNU); Song and Yamada 2009b], **JB** [Namwon-si, Mt. Jiri, Jeongryeongchi area, 26 Aug 2009, *Choi 4217* (JNU)], **GN** [Hamcheon-gun, Mt. Hwangmae, ridge, 3 Aug 2010, *Choi 7489* (JNU)], **GB** [Ulleung-gun, Seonginbong, 20 Oct 2010, *Choi 8757* (JNU); Amakawa 1959 as *Nardia sieboldii*, Hong 1962a, c, 1966 as *Nardia sieboldii*], **CN** [Choe 1979 as *Nardia sieboldii*], **GG** [Hong 1962a, 1966 as *Nardia sieboldii*], **GW** [Inje-gun, Mt. Seolak, Hangyeoryeong, 28 Aug 2009, *Choi 4265* (JNU); Hong 1962a, 1966 as *Nardia sieboldii*, Kim et al. 1995], **JG** [Kim and Hwang 1991], **HN** [Kim and Hwang 1991], **HB** [Kim and Hwang 1991], **Korea** [Iwatsuki and Mizutani 1972 as *Nardia sieboldii*, Váňa 1972; Choe and Yamada 1974; Inoue 1974; Váňa 1976; Choe and Choi 1980; Hong 1997, 2003]

*Nardia subclavata (Steph.) Amakawa – **CN** [Nonsan-si, Mt. Daedun, 31 Mar 2009, *Choi 3371* (JNU)]

***Neoorthocaulis* L.Söderstr., De Roo et Hedd.** (Anastrophyllaceae)

°***Neoorthocaulis attenuatus*** (Mart.) L.Söderstr., De Roo et Hedd. – **GW** [Kim and Hwang 1991 as *Barbilophozia gracilis*, Kim et al. 1995 as *Barbilophozia attenuata*], **HN** [Kim and Hwang 1991 as *Barbilophozia gracilis*], **HB** [Kim and Hwang 1991 as *Barbilophozia gracilis*], **Korea** [Hong 1997, 2003 as *Barbilophozia attenuata*]

***Neotrichocolea* S.Hatt.** (Neotrichocoleaceae)

*Neotrichocolea bissetii (Mitt.) S.Hatt. – **GN** [Hamyang-gun, Mt. Jiri, Chilseon valley, 28 Sep 2010, *Choi 8198* (JNU)]

***Nipponolejeunea* S.Hatt.** (Jubulaceae)

***Nipponolejeunea pilifera*** (Steph.) S.Hatt. – **JN** [Gurye-gun, Mt. Jiri, Nogodan, 29 Apr 2009, *Choi 3573* (JNU); Hong 1962a, 1966; Song and Yamada 2009b], **JB** [Musu-gun, Mt. Deogyu, Hyangjeokbong area, 10 Oct 2009, *Choi 4236* (JNU); Choe 1980], **GN** [Geochang-gun, Mt. Namdeogyu, top of mountain, 11 Nov 2010, *Choi 8957* (JNU); Hattori et al. 1962b, Choe 1980], **GB** [Ulleung-gun, Mireukbong, 21 Oct 2010, *Choi 8780* (JNU)], **Korea** [Mizutani 1961; Iwatsuki and Mizutani 1972; Choe and Yamada 1974; Inoue 1974, 1981; Choe and Choi 1980; Hong 1997, 2003]

***Nipponolejeunea subalpina*** (Horik.) S.Hatt. – **GW** [Inje-gun, Mt. Seolak, Socheong stony field, 12 May 2011, *Choi 110265* (JNU); Choe and Yamada 1979; Choe 1980, 1983], **Korea** [Inoue 1981; Choe and Choi 1980; Hong 1997, 2003]

***Nowellia* Mitt.** (Cephaloziaceae)

***Nowellia curvifolia*** (Dicks.) Mitt. – **JN** [Hong 1966, Choe 1983, Song and Yamada 2009b], **GN** [Hamyang-gun, Mt. Jiri, Chilseon valley, 28 Sep 2010, *Choi 8123* (JNU); Hattori et al. 1962b; Hong 1962a; Choe 1980, 1983], **GW** [Inje-gun, Mt. Seolak, Gugokdam valley, 14 Oct 2010, *Choi 8656Choi 8656* (JNU); Kim et al. 1995], **PN** [Gao and Chang 1983a, b], **JG** [Kim and Hwang 1991], **YG** [Gao and Chang 1983a, b; Kim and Hwang 1991], **Korea** [Schuster 1974; Choe and Yamada 1974; Choe and Choi 1980; Hong 1997, 2003]

***Odontoschisma* (Dumort.) Dumort.** (Odontoschismataceae)

***Odontoschisma denudatum*** (Mart.) Dumort. – **GN** [Hamyang-gun, Mt. Jiri, Chilseon valley, 28 Sep 2010, *Choi 8115* (JNU)], **GB** [Ulleung-gun, Mireukbong, 21 Oct 2010, *Choi 8782* (JNU)], **GW** [Inje-gun, Mt. Seolak, Gugokdam valley, 14 Oct 2010, *Choi 8656* (JNU); Kim and Hwang 1991; Kim et al. 1995], **PB** [Kim and Hwang 1991], **Korea** [Inoue 1981; Hong 1997, 2003]

°***Odontoschisma jishibae*** (Steph.) L.Söderstr. et Váňa – **GG** [Hong 1960b, 1962a, 1966 as *Cephaloziella jishibae*], **Korea** [Choe 1980 as *Iwatsukia jishibae*, Choe and Yamada 1974 as *Iwatsukia jishibae*, Choe and Choi 1980 as *Iwatsukia jishibae*, Hong 1997, 2003 as *Iwatsukia jishibae*]

***Odontoschisma pseudogrosseverrucosum*** Gradst., S.C.Aranda et Vanderp. – **JN** [Gurye-gun, Mt. Jiri, Nododan, 5 Aug 2010, *Choi 7555* (JNU)], **GN** [Geochang-gun, Mt. Namdeogyu, top of mountain, 11 Nov 2010, *Choi 8956* (JNU)], **GW** [Inje-gun, Mt. Seolak, Baekdamsam Temple valley, 11 May 2011, *Choi 110115* (JNU); Kim and Hwang 1991 as *Odontoschisma grosseverrucosum*], **Korea** [Hong 1997, 2003 as *Odontoschisma grosseverrucosum*]

***Pallavicinia* Gray** (Pallaviciniaceae)

***Pallavicinia subciliata*** (Austin) Steph. – **JJ** [Jeju-si, Musu stream, 28 Oct 2010, *Choi 8828* (JNU)]

***Pedinophyllum* Lindb. ex Nordst.** (Plagiochilaceae)

*Pedinophyllum interruptum (Nees.) Kaal. – **JB** [Muju-gun, Mt. Deogyu, 27 Jun 2008, *Choi 10858* (JNU)], **GW** [Samcheok-si, Mt. Deokhang, ridge, 20 Jul 2010, *Choi 7467* (JNU)]

***Pedinophyllum truncatum*** (Steph.) Inoue – **JJ** [Hong and Kim 1961b, Song and Yamada 2006], **JN** [Suncheon-si, Mt. Jogyeo, Seonamsa Temple, 7 Dec 2010, *Choi 9110* (JNU)], **JB** [Buan-gun, Mt. Naebyen, Beadrock near road, 10 Mar 2009, *Choi 9110* (JNU)], **GN** [Sancheong-gun, Mt. Jiri, Jangsanri valley, 13 Jun 2009, *Choi 3608* (JNU); Hong 1966 as Pedinophyllum interruptum subsp. truncatum, Song and Yamada 2009a], **GB** [Ulleung-gun, Seonginbong, 20 Oct 2010, *Choi 8763* (JNU); Hong 1966 as Pedinophyllum interruptum subsp. truncatum], **CB** [Hong 1962d], **GG** [Hong and Kim 1961a; Hong 1962a as Pedinophyllum interruptum subsp. truncatum], **GW** [Inje-gun, Mt. Seolak, Hangyeoryeong, 21 Sep 2009, *Choi 5099* (JNU); Hong 1962a, 1966 as Pedinophyllum interruptum subsp. truncatum and Pedinophyllum interruptum var. jungermannioides, Hong 1962b], **PN** [Gao and Chang 1983a, 1983b as *Pedinophyllum major-perianthium*], **JG** [Kim and Hwang 1991], **HB** [Kim and Hwang 1991], **Korea** [Iwatsuki and Mizutani 1972; Choe and Yamada 1974; Choe and Choi 1980; Hong 1997, 2003]

***Pellia* Raddi** (Pelliaceae)

°***Pellia epiphylla*** (L.) Corda – **HWN** [Kim and Hwang 1991], **HWB** [Kim and Hwang 1991], **PB** [Kim and Hwang 1991], **YG** [Kim and Hwang 1991], **HN** [Gao and Chang 1983a, b; Yamada and Choe 1997], **HB** [Kim and Hwang 1991], **Korea** [Hong 1997, 2003]

***Pellia neesiana*** (Gottsche) Limpr. – **JJ** [Jeju-si, Musu stream, Goangryeong 2^nd^ Bridge, 18 Mar 2012, *Choi 120118* (JNU); Horikawa 1935b, 1936b, Hattori 1944a; Hattori et al. 1962a; Hong 1962a; Choe 1980; Song and Yamada 2006], **JN** [Suncheon-si, Mt. Jogyeo, Seonamsa Temple, 7 Dec 2010, *Choi 9113* (JNU)], **JB** [Musu-gun, Mt. Deogyu, Hyangjeokbong area, 10 Oct 2009, *Choi 5281* (JNU)], **GN** [Sancheong-gun, Mt. Jiri, Hakseupwon area, 14 Jun 2009, *Choi 3694* (JNU)], **GB** [Ulsan-si, Mt. Sinbul, Danjo neup, 1 Oct 2010, *Choi 8327* (JNU)], **GW** [Inje-gun, Mt. Seolak, Ssangyoung waterfall, 14 Oct 2010, *Choi 8626* (JNU); Hong 1962a; Choe 1980; Kim et al. 1995], **PB** [Kim and Hwang 1991], **YG** [Kim and Hwang 1991], **HN** [Kim and Hwang 1991], **HB** [Kim and Hwang 1991], **Korea** [Horikawa 1939; Hattori 1944a; Choe and Yamada 1974; Choe and Choi 1980; Hong 1997, 2003]

***Plagiochasma* Lehm.** (Aytoniaceae)

***Plagiochasma japonicum*** (Steph.) C.Massal. – **GG** [Choe 1980], **GW** [Jeongseon-gun, River Donggang, near ther Donggang, 16 Aug 2010, *Choi 7929* (JNU); Choe 1980, 1983, Kim et al. 1995], **HWN** [Stephani 1917a as *Plagiochasma koreanum*, Bischler 1979, Kim and Hwang 1991], **PN** [Kim and Hwang 1991], **PB** [Kim and Hwang 1991], **HB** [Kim and Hwang 1991], **Korea** [Miller et al. 1983; Long and Grolle 1990; Choe and Choi 1980; Hong 1997, 2003]

***Plagiochasma pterospermum*** C.Massal. – **GB** [Choe 1975 as *Plagiochasma intermedium*], **GG** [Hong 1962a as *Plagiochasma intermedium*], **GW** [Jeongseon-gun, Mt. Seokbyeong, ridge, 30 Sep 2009, *Choi 5270* (JNU); Kim and Hwang 1991 as *Plagiochasma intermedium*], **HWN** [Kim and Hwang 1991 as *Plagiochasma intermedium*], **PN** [Kim and Hwang 1991 as *Plagiochasma intermedium*], **PB** [Kim and Hwang 1991 as *Plagiochasma intermedium*], **Korea** [Hattori 1950 as *Plagiochasma intermedium*, Hong 1997, 2003]

***Plagiochila*** (Dumort.) Dumort. (Plagiochilaceae)

°***Plagiochila fruticosa*** Mitt. – **HWN** [Kim and Hwang 1991], **YG** [Kim and Hwang 1991], **Korea** [Hong 1997, 2003]

*Plagiochila furcifolia Mitt. – **JJ** [Jeju-si, Bijarim, 2 May 2012, *Choi 120431* (JNU)]

***Plagiochila gracilis*** Lindenb. et Gottsche – **JJ** [Hattori et al. 1962a as Plagiochila firma subsp. rhizophora, Hong 1962a as *Plagiochila rhizophora*, Song and Yamada 2006], **JN** [Hong 1962a as *Plagiochila rhizophora*, Choe 1980 as Plagiochila firma subsp. rhizophora, Song and Yamada 2009b], **GN** [Hamyang-gun, Mt. Jiri, Cheonwnagbong, 29 Sep 2010, *Choi 8234* (JNU); Hattori et al 1962b as as Plagiochila firma subsp. rhizophora, Choe 1980 as Plagiochila firma subsp. rhizophora], **GW** [Inje-gun, Mt. Seolak, Hangyeoryeong, 21 Sep 2009, *Choi 5068* (JNU); Hong 1962a, 1962b as *Plagiochila rhizophora*, Choe 1980 as Plagiochila firma subsp. rhizophora,], **Korea** [Choe and Yamada 1974 as Plagiochila firma subsp. rhizophora, Choe and Choi 1980 as Plagiochila firma subsp. rhizophora, Hong 1997, 2003]

***Plagiochila hakkodensis*** Steph. – **JJ** [Jeju-si, Seogeomeun Oreum, 20 Sep 2011, *Choi 111005* (JNU); Hattori et al. 1962a; Hong 1962a; Choe 1980; Song and Yamada 2006], **JN** [Hong 1962a] , **GN** [Sancheong-gun, Mt. Jiri, below Cheonwangbong, 15 Jun 2009, *Choi 3818* (JNU); Hong 1962a; Choe 1980; Song and Yamada 2009a], **GG** [Hong 1962a], **GW** [Hong 1962a; Kim and Hwang 1991], **Korea** [Choe and Yamada 1974; Choe and Choi 1980; Hong 1997, 2003]

***Plagiochila ovalifolia*** Mitt. – **JJ** [Jeju-si, Musu stream, 28 Oct 2010, *Choi 8837* (JNU); Horikawa 1934d; Hattori et al. 1962a; Hong 1962a as Plagiochila ovalifolia var. miyoshiana, Inoue 1962 as *Plagiochila quelpaertensis*, Hong 1966 as Plagiochila asplenioides subsp. ovalifolia and *Plagiochila quelpaertensis*, Choe 1980, 1983 as *Plagiochila quelpaertensis*, Song and Yamada 2006], **JN** [Haenam-gun, Mt. Duryun, Duryunsa Temple, 23 Apr 2010, *Choi 7305* (JNU); Hong 1962a, 1966 as Plagiochila asplenioides subsp. ovalifolia, Song and Yamada 2009b], **JB** [Buan-gun, Mt. Naebyen, Jikso Waterfall, 10 Mar 2009, *Choi 3308* (JNU); Hong 1966 as Plagiochila asplenioides subsp. ovalifolia], **GN** [Geoje-si, Oryung reservoir, 16 Mar 2011, *Choi 110026* (JNU); Hattori et al. 1962b, Hong 1966 as as Plagiochila asplenioides subsp. ovalifolia, Song and Yamada 2009a], **GB** [Cheongsong-gun, Mt. Juwang, Weoloe valley, 9 Nov 2010, *Choi 8942* (JNU); Hong 1962a, 1966 as as Plagiochila asplenioides subsp. ovalifolia], **CN** [Horikawa 1934d; Hong 1962a, 1966 as as Plagiochila asplenioides subsp. ovalifolia, Choe 1979], **GG** [Hong and Kim 1961a; Hong 1962a, 1966 as as Plagiochila asplenioides subsp. ovalifolia], **GW** [Inje-gun, Mt. Seolak, Baekdamsa Temple, 28 Aug 2009, *Choi 5278* (JNU); Hong 1962a as Plagiochila ovalifolia var. miyoshiana, Hong 1966 as as Plagiochila asplenioides subsp. ovalifolia, Kim et al. 1995], **HWN** [Kim and Hwang 1991], **PN** [Kim and Hwang 1991], **JG** [Kim and Hwang 1991], **YG** [Kim and Hwang 1991], **HN** [Kim and Hwang 1991], **HB** [Kim and Hwang 1991], **Korea** [Horikawa 1939, Hattori 1944a as Plagiochila ovalifolia var. orbicularis, Shin 1968, 1970; Iwatsuki and Mizutani 1972; Choe and Yamada 1974; Inoue 1974, 1981; Choe and Choi 1980 as *Plagiochila quelpaertensis*, Li 1985; Hong 1997 as *Plagiochila quelpaertensis*, Hong 2003]

***Plagiochila parvifolia*** Lindenb. – **JN** [Sinan-gun, Is. Gageo, Mt. Doksil, 2 Mar 2010, *Choi 7187* (JNU); Hong 1962a, 1966 as *Plagiochila yokogurensis*, Song and Yamada 2009b], **GN** [Horikawa 1951a; Hattori et al. 1962b as *Plagiochila yokogurensis*], **CN** [Choe 1972, 1979 as *Plagiochila yokogurensis*], **PB** [Kim and Hwang 1991 as *Plagiochila yokogurensis*], **Korea** [Horikawa 1955 as *Plagiochila yokogurensis*, Shin 1968 as *Plagiochila yokogurensis*, Iwatsuki and Mizutani 1972 as *Plagiochila yokogurensis*, Choe and Yamada 1974 as *Plagiochila yokogurensis*, Hong 1997 as *Plagiochila yokogurensis*, Hong 2003]

***Plagiochila porelloides*** (Torr. ex Nees) Lindenb. – **JJ** [Jeju-si, Mt. Halla, Baekrokdam, 8 Aug 2010, *Choi 7753* (JNU); Hong and Kim 1961b as *Plagiochila satoi*, Hattori et al. 1962a as *Plagiochila satoi*, Hong 1962a, 1966 as *Plagiochila satoi*, Song and Yamada 2006], **JN** [Sinan-gun, Is. Gageodo, Bolryemi seashore 22 Apr 2012, *Choi 120401* (JNU); Hong 1962a, 1966 as *Plagiochila satoi*, Song and Yamada 2009b], **JB** [Jeongeup-si, Mt. Naejang, Geumseon valley, 16 Mar 2009, *Choi 3454* (JNU)], **GN** [Hamyang-gun, Mt. Jiri, Chilseon valley, 28 Sep 2010, *Choi 8147* (JNU); Hattori et al. 1962b as *Plagiochila satoi*, Hong 1966 as *Plagiochila satoi*, Song and Yamada 2009a], **GB** [Ulleung-gun, Mireukbong, 21 Oct 2010, *Choi 8791* (JNU); Hong 1966 as *Plagiochila satoi*], **GG** [Hong 1966 as *Plagiochila satoi*], **GW** [Inje-gun, Mt. Seolak, Kkeucheong, 21 Sep 2009, *Choi 5105* (JNU); Hong 1962a, 1966 as *Plagiochila satoi*, Kim et al. 1995 as *Plagiochila satoi*], **YG** [Kim and Hwang 1991 as *Plagiochila satoi*], **HN** [Kim and Hwang 1991 as *Plagiochila satoi*], **HB** [Kim and Hwang 1991 as *Plagiochila satoi*], **Korea** [Choe and Yamada 1974 as *Plagiochila satoi*, Choe and Choi 1980 as *Plagiochila satoi*, Hong 1997 as *Plagiochila satoi*, Hong 2003]

***Plagiochila sciophila*** Nees – **JJ** [Jeju-si, Nokkome Oreum, 28 Oct 2010, *Choi 8804* (JNU); Hattori et al. 1962a as *Plagiochila japonica*, Hong 1962a, 1966 as *Plagiochila japonica*, Song and Yamada 2006], **JN** [Sinan-gun, Is. Gageo, Lighthouse-Mt. Doksil, 2 Mar 2010, *Choi 7230* (JNU); Hong 1966 as *Plagiochila japonica*, Song and Yamada 2009b], **JB** [Hong 1966 as *Plagiochila japonica*], **GN** [Sancheong-gun, Mt. Jiri, Jangsanri valley, 13 Jun 2009, *Choi 3660* (JNU)], **GB** [Ulleung-gun, Seonginbong, 20 Oct 2010, *Choi 8719* (JNU); Hong 1962a, 1962c, 1966 as *Plagiochila japonica*], **CN** [Choe 1979 as Plagiochlia acanthophylla subsp. japonica], **GG** [Yamada and Choe 1997], **GW** [Hong 1966 as *Plagiochila japonica*, Kim and Hwang 1991 as Plagiochlia acanthophylla subsp. japonica, Kim et al. 1995 as Plagiochlia acanthophylla subsp. japonica], **Korea** [Iwatsuki and Mizutani 1972 as Plagiochlia acanthophylla subsp. japonica, Choe and Yamada 1974 as Plagiochlia acanthophylla subsp. japonica, Choe and Choi 1980 as Plagiochila acanthophylla subsp. japonica, Hong 1997, 2003]

***Plagiochila semidecurrens*** (Lehm. et Lindenb.) Lindenb. – **JJ** [Hattori et al. 1962a as Plagiochila semidecurrens var. grossidens, Hong 1962a, 1966; Song and Yamada 2006], **JN** [Hong 1962a, 1966 as Plagiochila semidecurrens var. grossidens, Song and Yamada 2009b], **GN** [Hamyang-gun, Mt. Jiri, Cheonwangbong area, 11 Oct 2019, *Choi 1910268* (JNU); Hattori et al. 1962b as Plagiochila semidecurrens var. grossidensChoe 1980], **PB** [Kim and Hwang 1991], **HB** [Kim and Hwang 1991], **Korea** [Iwatsuki and Mizutani 1972; Choe and Yamada 1974; Inoue 1976; Choe and Choi 1980; Hong 1997, 2003]

*Plagiochila shangaica Steph. – **JJ** [Seogwipo-si, Andeok valley, 16 Mar 2012, *Choi 120045* (JNU)], **JN** [Goheung-gun, Is. Oenarodo, Mt. Bongrae, valley, 20 May 2011, *Choi 110577* (JNU)], **GN** [Geoje-si, Mt. Noja, Forest lodge, 17 Mar 2011, *Choi 110041* (JNU)]

***Plagiochila trabeculata*** Steph. – **JJ** [Jeju-si, Seogeomeun Oreum, 21 Jun 2011, *Choi 110859* (JNU)], **JB** [Jeongeup-si, Mt. Naejang, Geumseon valley, 16 Mar 2009, *Choi 3453* (JNU)], **GN** [Sancheong-gun, Mt. Jiri, below Cheonwangbong, 15 Jun 2009, *Choi 3811* (JNU)] **GW** [Kim et al. 1995], **PB** [Kim and Hwang 1991], **JG** [Kim and Hwang 1991], **YG** [Kim and Hwang 1991], **HN** [Kim and Hwang 1991], **Korea** [Iwatsuki and Mizutani 1972; Choe and Yamada 1974; Inoue 1974; Choe 1980; Choe and Choi 1980; Inoue 1981; Hong 1997, 2003]

***Plagiochilion* S.Hatt.** (Plagiochilaceae)

***Plagiochilion mayebarae*** S.Hatt. – **JJ** [Jeju-si, Mt. Halla, Baekrokdam, 8 Aug 2010, *Choi 7764* (JNU); Choe and Yamada 1998], **GN** [Hamyang-gun, Mt. Jiri, Cheonwnagbong, 28 Sep 2010, *Choi 9132* (JNU); Choe and Yamada 1998; Song and Yamada 2009b]

***Plectocolea* (Mitt.) Mitt.** (Solenostomataceae)

***Plectocolea comata*** (Nees) S.Hatt. – **JJ** [Jeju-si, Che Oruem, 27 Aug 2010, *Choi 8061* (JNU)], **JN** [Haenam-gun, Mt. Duryun, Taehengsa Temple valley, 18 May 2011, *Choi 110495* (JNU)], **GB** [Choe 1980 as *Jungermannia comata*], **CB** [Choe 1980 as *Jungermannia comata*], **GG** [Hong and Kim 1961a as *Jungermannia comata*, Hong 1962a as *Jungermannia comata*, Choe 1980 as *Jungermannia comata*], **GW** [Hong 1966 as *Jungermannia comata*], **Korea** [Váňa 1972 as *Jungermannia comata*, Choe and Yamada 1974 as *Jungermannia comata*, Choe and Choi 1980 as *Jungermannia comata*, Miller et al. 1983 as *Jungermannia comata*, Hong 1997, 2003 as *Jungermannia comata*]

***Plectocolea erecta*** Amakawa – **JJ** [Mt. Halla, elev. 1,687 m, *Choi 110379* (JNU)], JN [Choe 1980 as *Jungermannia erecta*], **JB** [Muju-gun, Mt. Deogyu, 7 Jun 2008, *Choi 614* (JNU)], **GN** [Hapcheon-gun, Mt. Gaya, top of mountain, elev. 1,350 m, 22 Jun 2010, *Choi 7424a* (JNU)], **GB** [Ulleung-gun, Jeodong, Bongrae waterfall, *Choi site4-224* (JNU)], **CN** [Nonsan-si, Mt. Daedun, elev. 343 m, *Choi 3366* (JNU)], **GW** [Inje-gun, Mt. Seolak, Ssangyoung waterfall, 14 Oct 2010, elev. 937 m, *Choi 8628* (JNU); Kim et al. 1995 as *Jungermannia erecta*], **Korea** [Hong 2003 as *Jungermannia erecta*]

*Plectocolea granulata (Steph.) Bakalin – **JJ** [Seogwipo-si, Sumeunmulbyengdwi, elev. 992 m, 26 Aug 2010, *Choi 8019* (JNU)]

*Plectocolea grossitexta (Steph.) S.Hatt. – **JJ** [Seogwipo-si, Sumeunmulbyengdwi, elev. 992 m, 26 Aug 2010, *Choi 8057* (JNU)]

**Plectocolea infusca var. infusca** Mitt. – **JJ** [Seogwipo-si, Bolrae Oreum, elev. 1,230 m, 1 Nov 2011, *Choi 111429* (JNU); Hong 1966 as *Jungermannia infusca*, Miller et al. 1983 as *Jungermannia infusca*, Song and Yamada 2006 as *Jungermannia infusca*], **JN** [Haenam-gun, Mt. Dureun, elev. 262 m, 18 May 2011, *Choi 110500* (JNU); Hong 1962a, 1966 as *Jungermannia infusca*, Song and Yamada 2009b as *Jungermannia infusca*], **JB** [Muju-gun, Mt. Deogyu, 7 Jun 2008, *Choi 596* (JNU); Hong 1966 as *Jungermannia infusca*], **GN** [Mt. Hwangmae, elev. 908 m, 3 Aug 2010, *Choi 7500* (JNU)], **GB** [Mt. Sinbul, elev. 987 m, 1 Oct 2010, *Choi 8328* (JNU); Hong 1966 as *Jungermannia infusca*], **CN** [Hong 1962a, 1966 as *Jungermannia infusca*], **GG** [Hong 1962a, 1966 as *Jungermannia infusca*], **GW** [Hong 1966 as *Jungermannia infusca*], **PN** [Kim and Hwang 1991 as *Jungermannia infusca*], **JG** [Kim and Hwang 1991 as *Jungermannia infusca*], **Korea** [Váňa and Inoue 1983 as *Jungermannia infusca*, Choe and Yamada 1974; Choe and Choi 1980; Hong 1997, 2003 as *Jungermannia infusca*]

**Plectocolea infusca var. recondita** Bakalin – **JJ** [Seogwipo-si, Bolrae Oreum, elev. 1,230 m, 1 Nov 2011, *Choi 111419* (JNU); Hattori et al. 1962a as Jungermannia infusca var. ovicalyx, Choe 1980 as Jungermannia infusca var. ovicalyx, Song and Yamada 2006 as Jungermannia infusca var. ovicalyx], **JB** [Muju-gun, Mt. Deogyu, *Choi 417* (JNU)], GN [Mt. Hangmae, elev. 1,057 m, 9 Sep 2009, *Choi 4388* (JNU)], **CB** [Hong 1962a, 1966 as Jungermannia infusca var. ovicalyx, Choe 1980 as Jungermannia infusca var. ovicalyx], **GG** [Hong 1962a, 1966 as Jungermannia infusca var. ovicalyx, Choe 1980 as Jungermannia infusca var. ovicalyx], **GW** [Jeongseon-gun, Seokbyeong Mt. elev. 956 m, *Bakalin Kor-15-13-15* (JNU, VBGI)], **Korea** [Choe and Yamada 1974 as Jungermannia infusca var. ovicalyx, Choe and Choi 1980 as Jungermannia infusca var. ovicalyx, Hong 1997, 2003 as Jungermannia infusca var. ovicalyx]

*Plectocolea kurilensis (Bakalin) Bakalin et Vilnet – **JJ** [Seogwipo-si, Bolrae Oreum, elev. 1,230 m, *Choi 120737* (JNU)]

***Plectocolea ovalifolia*** (Amakawa) Bakalin et Vilnet – **JJ** [Hattori et al. 1962a as Jungermannia infusca var. ovalifolia, Hong 1962a, 1966 as Jungermannia infusca var. ovalifolia, Choe 1980 as Jungermannia infusca var. ovalifolia, Song and Yamada 2006 as Jungermannia infusca var. ovalifolia], **JN** [Gurye-gun, Jirisan Mt., elev. 1,222 m, *Choi 3522* (JNU)], **JB** [Namwon-si, Jirisan Mt., elev. 1,202 m, *Choi 8281* (JNU)], **GN** [Hapcheon-gun, Mt. Gaya, top of mountain, elev. 1,350 m, 22 Jun 2010, *Choi 7429* (JNU)], **GB** [Ulleung-gun, Jeo-dong, Bongrae waterfall, *Choi site 4-234* (JNU)], **CB** [Choe 1980 as Jungermannia infusca var. ovalifolia], **GG** [Hong 1962a as Jungermannia infusca var. ovalifolia, Choe 1980 as Jungermannia infusca var. ovalifolia], **GW** [Inje-gun, Mt. Seolak, Hangyeoryeong, 28 Aug 2009, *Choi 4255* (JNU)], **Korea** [Choe and Yamada 1974 as Jungermannia infusca var. ovalifolia, Choe and Choi 1980 as Jungermannia infusca var. ovalifolia, Hong 1997, 2003 as Jungermannia infusca var. ovalifolia]

***Plectocolea radicellosa*** (Mitt.) Mitt. – **JJ** [Jeju-si, Musu stream, 28 Oct 2010, *Choi 8824* (JNU); Váňa 1975 as *Jungermannia radicellosa*, Choe 1980, 1983 as *Jungermannia radicellosa*], **CN** [Gyeryong-si, Mt. Gyeryong, elev. 274 m, *Choi 4115* (JNU)], **Korea** [Choe and Choi 1980 as *Jungermannia radicellosa*, Hong 1997, 2003 as *Jungermannia radicellosa*]

***Plectocolea rosulans*** (Steph.) S.Hatt. – **JJ** [Seogwipo-si, Dongsu Brigde, 2 Nov 2011,*Choi 111455* (JNU)], **JN** [Goheung-gun, Mt. Palyeoung, Forest lodge valley, 23 Jun 2009, *Choi 4012* (JNU)], **JB** [Muju-gun, Mt. Deogyu, 25 Jun 2018, *Choi 724* (JNU)], **GN** [Sancheong-gun, Mt. Jiri, Jangsanri valley, 13 Jun 2009, *Choi 3656* (JNU)], **CN** [Gyeryong-si, Mt. Gyeryong, Temple Donghaksa valley, 8 Jul 2009, *Choi 4070* (JNU)].

*Plectocolea torticalyx (Steph.) S.Hatt. – **JB** [Namwon-si, Jirisan Mt., elev. 1,300 m, Choi 6071 (JNU)], **GN** [Sancheong-gun, Mt. Jiri, Jungsanri valley, 16 Jun 2009, *Choi 3856* (JNU)].

***Plectocolea truncata*** (Nees) Herzog – **JJ** [eju-si, Che Oruem, 27 Aug 2010, *Choi 8059* (JNU)], **CN** [Gyeryong-si, Mt. Gyeryong, elev. 290 m, Temple Donghaksa valley, 8 Jul 2009, *Choi 4096* (JNU)], **GG** [Hong 1962a, 1966 as *Jungermannia tsukushinensis*], **GW** [Choe 1980, 1983 as *Jungermannia truncata*], **Korea** [Choe and Yamada 1974 as *Jungermannia truncata*, Hattori 1975a as *Jungermannia virgata*, Choe and Choi 1980 as *Jungermannia truncata*, Hong 1997, 2003 as *Jungermannia truncata*]

***Plectocolea virgata*** Mitt. – **JJ** [Seogwipo-si, Hyodon stream, 29 Oct 2011, *Choi 111260* (JNU); Hattori et al. 1962a as *Jungermannia virgata*, Hong 1962a; Hong 1966 as *Jungermannia virgata*, Choe 1980 as *Jungermannia virgata*, Song and Yamada 2006 as *Jungermannia virgata*], **JN** [Hattori et al. 1962b as *Jungermannia virgata*, Hong 1962a as *Jungermannia virgata*, Song and Yamada 2009b as *Jungermannia virgata*], **GN** [Hattori et al. 1962b as *Jungermannia virgata*, Choe 1980 as *Jungermannia virgata*], **Korea** [Choe and Yamada 1974 as *Jungermannia virgata*, Choe and Choi 1980 as *Jungermannia virgata*, Váňa and Inoue 1983 as *Jungermannia virgata*, Hong 1997, 2003 as *Jungermannia virgata*]

***Plicanthus* R.M.Schust.** (Anastrophyllaceae)

***Plicanthus birmensis*** (Steph.) R.M.Schust. – **JJ** [Jeju-si, Seogeomeun Oreum, 20 Sep 2011, *Choi 111022b* (JNU)], **JN** [Goheung-gun, Is. Oenarodo, Mt. Bongrae, valley, 20 May 2011, *Choi 110595* (JNU); Song and Yamada 2009b], **JB** [Jangsu-gun, Mt. Jangan, Banghwadong valley, 30 Apr 2009, *Choi 3580* (JNU)], **GN** [Geoje-si, Oryung reservoir, 16 Mar 2011, *Choi 110105* (JNU); Horikawa 1951a as *Temnoma birmense*], **GB** [Cheongsong-gun, Mt. Juwang, The 2^nd^ waterfall, 8 Nov 2010, *Choi 8916* (JNU)], **CN** [Choe 1975, 1979 as *Chandonanthus birmensis*], **GG** [Hong 1960b as *Temnoma birmense*; Hong 1962a, 1966 as *Chandonanthus birmensis*,Choe 1975 as *Chandonanthus birmensis*], **GW** [Inje-gun, Mt. Seolak, Baekdamsam Temple valley, 11 May 2011, *Choi 110112* (JNU); Choe 1975, Gao and Chang 1983a, 1983b as *Chandonanthus birmensis*, Kim et al. 1995 as *Chandonanthus birmensis*], **HN** [Kim and Hwang 1991 as *Chandonanthus birmensis*], **HB** [Kim and Hwang 1991 as *Chandonanthus birmensis*], **Korea** [Choe and Yamada 1974 as *Chandonanthus birmensis*, Choe and Choi 1980 as *Chandonanthus birmensis*, Li 1985 as *Chandonanthus birmensis*, Hong 1997, 2003 as *Chandonanthus birmensis*]

***Plicanthus hirtellus*** (F.Weber) R.M.Schust. – **GW** [Sokcho-si, Mt. Seolak, Socheongbong, 12 Oct 2010, *Choi 8431* (JNU)], **HN** [Kim and Hwang 1991 as as *Chandonanthus hirtellus*], Korea [Hong 1997, 2003 as *Chandonanthus hirtellus*]

***Porella* L.** (Porellaceae)

**Porella acutifolia subsp. tosana** (Steph.) S.Hatt. – **JJ** [Seogwipo-si, Suak valley, 11 Oct 2012, *Choi 121048* (JNU)], **GN** [Hamyang-gun, Mt. Jiri, Hansin valley, 3 Apr 2010, *Choi 7270* (JNU)], **Korea** [Stephani 1910a as *Madotheca tosana*, Hong 1962a as *Porella tosana*, Iwatsuki and Mizutani 1972 as Porella campylophylla subsp. tosana, Choe and Yamada 1974 as Porella campylophylla subsp. tosana, Choe 1980 as Porella campylophylla subsp. tosana, Choe and Choi 1980 as Porella campylophylla subsp. tosana, Kim and Hwang 1991 as Porella campylophylla subsp. tosana, Hong 1997 as *Porella acutifolia*, Hong 2003]

**Porella caespitans var. cordifolia** (Steph.) S.Hatt. ex T.Katag. et T.Yamag. – **JJ** [Jeju-si, Mt. Halla, Seongpanak-Baekrokdam, 8 Aug 2010, *Choi 7712* (JNU); Hong et al. 1961 as *Porella setigera*, Hattori et al. 1962a as *Porella setigera*, Hong 1962a, 1966 as *Porella setigera*, Song and Yamada 2006], **JN** [Goheung-gun, Is. Oenarodo, Mt. Bongrae, 20 May 2011, *Choi 110587* (JNU); Uno and Takahashi 1940 as *Madotheca setigera*, Hong 1966 as *Porella setigera*, Song and Yamada 2009b], **JB** [Jeongeup-si, Mt. Naejang, Geumseon valley, 16 Mar 2009, *Choi 3484* (JNU); Hong 1966 as *Porella setigera*], **GN** [Hamyang-gun, Mt. Jiri, Chilseon valley, 28 Sep 2010, *Choi 8136* (JNU); Horikawa 1934c as *Madotheca setigera*, Hong 1966 as *Porella setigera*], **GB** [Yeongju-si, Mt. Sobaek, Birobong area, 2 Sep 2009, *Choi 4296* (JNU); Hong 1966 as *Porella setigera*], **CN** [Horikawa 1934c as *Madotheca setigera*], **CB** [Hong 1962a, d, 1966 as *Porella setigera*], **GG** [Hong 1960c as *Porella setigera*, Hong and Kim 1961a as *Porella setigera*, Hong 1962a, 1966 as *Porella setigera*], **GW** [Inje-gun, Mt. Seolak, Hangyeoryeong, 28 Aug 2009, *Choi 4281* (JNU); Hong 1962a as *Porella setigera*, Hattori 1970 as Porella caespitans var. setigera, Kim et al. 1995 as *Porella setigera*], **PB** [Kim and Hwang 1991 as *Porella setigera*], **JG** [Kim and Hwang 1991 as *Porella setigera*], **HN** [Kim and Hwang 1991 as *Porella setigera*], **HB** [Kim and Hwang 1991 as *Porella setigera*], **Korea** [Inoue 1955 as *Porella setigera*, Shin 1968 as *Porella setigera*, Iwatsuki and Mizutani 1972 as Porella caespitans var. setigera, Inoue 1974 as Porella caespitans var. setigera, Hattori 1975a, 1978 as Porella caespitans var. setigera, Hattori and Zhang 1985 as Porella caespitans var. setigera, Choe and Choi 1980 as Porella caepitans var. setigera, Long and Grolle 1990 as Porella caespitans var. setigera, Hong 1997 as *Porella setigera*, Hong 2003]

*Porella chinensis (Steph.) S.Hatt. – **JN** [Wando-gun, Sangwhangbong, valley, 9 Feb 2010, *Choi 7006* (JNU)], **GB** [Cheongsong-gun, Mt. Juwang, Jabang waterfall, 8 Nov 2010, *Choi 8904* (JNU)], **GW** [Samcheok-si, Mt. Deokhang, 14 Oct 2009, *Choi 7006* (JNU)]

°***Porella densifolia*** (Steph.) S.Hatt. – **JJ** [Song and Yamada 2006 as Porella densifolia var. fallax], **GW** [Kim et al. 1995], **PN** [Kim and Hwang 1991], **JG** [Kim and Hwang 1991], **HB** [Kim and Hwang 1991], **Korea** [Hattori and Zhang 1985; Li 1985; Luo 1989; Hong 1997, 2003]

***Porella fauriei*** (Steph.) S.Hatt. – **JJ** [Jeju-si, Mt. Halla, Seongpanak- Baekrokdam, 8 Aug 2010, *Choi 7717* (JNU); Ando 1960 as Porella vernicosa subsp. fauriei, Hong et al. 1961 as Porella vernicosa subsp. fauriei, Hattori et al. 1962a; Hong 1962a, 1966; Hattori 1978; Song and Yamada 2006], **JN** [Hong 1962a, 1966; Song and Yamada 2009b], **GN** [Sancheong-gun, Mt. Jiri, Jangteomok shelter, 16 Jun 2009, *Choi 3841* (JNU); Hattori et al. 1962b], **GB** [Ulleung-gun, Seonginbong, 20 Oct 2010, *Choi 8727* (JNU); Hong 1962a, 1966, Choe 1980], **GW** [Inje-gun, Mt. Seolak, Hangyeoryeong, 21 Sep 2009, *Choi 5090* (JNU); Hong and Kim 1960, Hong 1960a as as Porella vernicosa subsp. fauriei, Hong 1962a, 1966, Choe 1980, Kim et al. 1995], **JG** [Kim and Hwang 1991], **PB** [Kim and Hwang 1991], **HN** [Kim and Hwang 1991], **HB** [Kim and Hwang 1991], **Korea** [Iwatsuki and Mizutani 1972; Choe and Yamada 1974; Inoue 1976, 1981; Choe and Choi 1980; Hong 1997, 2003]

***Porella gracillima*** Mitt. – **JN** [Wando-gun, Sangwhangbong, valley, 9 Feb 2010, *Choi 7006* (JNU); Hong 1966], **JB** [Hong 1966, Choe 1980], **GB** [Hong 1966; Choe 1980], **GG** [Ando 1960 as Porella vernicosa subsp. gracillima, Hong 1960b, c as Porella vernicosa subsp. gracillima, Hong 1962a], **GW** [Jeongseon-gun, River Donggang, near ther Donggang, 16 Aug 2010, *Choi 7913* (JNU); Hong 1962a, 1966, Choe 1980, Kim et al 1995], **JG** [Kim and Hwang 1991], **PB** [Kim and Hwang 1991], **HB** [Kim and Hwang 1991], **Korea** [Hattori 1969; Iwatsuki and Mizutani 1972; Luo 1989; Choe and Yamada 1974; Choe and Choi 1980; Hong 1997, 2003]

***Porella grandiloba*** Lindb. – **JJ** [Jeju-si, Nokkome Oreum, 28 Oct 2010, *Choi 8812* (JNU); Hong et al. 1961; Hattori et al. 1962a; Hong 1962a, 1966; Song and Yamada 2006], **JN** [Goheung-gun, Is. Oenarodo, Mt. Bongrae, valley, 20 May 2011, *Choi 110575* (JNU); Hong 1966; Song and Yamada 2009b], **JB** [Namwon-si, Mt. Jiri, Baemsagol valley, 19 Jun 2009, *Choi 3884* (JNU); Hong 1966], **GN** [Hong 1966, Song and Yamada 2009a], **GB** [Ulleung-gun, Seonginbong, 20 Oct 2010, *Choi 8717* (JNU); Hong 1962a, c, 1966], **CN** [Choe 1972, 1979], **CB** [Hong 1962a, d, 1966], **GG** [Hong 1960b, c; Hong and Kim 1961a; Hong 1962a, 1966], **GW** [Hwancheon-gun, *Cypripedium japonicum* area, 24 Jul 2009, *Choi 4144* (JNU); Hong and Kim 1960; Hong 1960a, 1962a, 1966; Gao and Chang 1983a, b; Kim et al. 1995], **HWN** [Kim and Hwang 1991], **PN** [Kim and Hwang 1991], PB [Kim and Hwang 1991], **YG** [Kim and Hwang 1991], **HN** [Kim and Hwang 1991], **HB** [Kim and Hwang 1991], **Korea** [Iwatsuki and Mizutani 1972; Inoue 1976; Hattori 1978; Aur and Zhang 1985; Choe and Yamada 1974; Choe and Choi 1980; Hong 1997, 2003]

***Porella japonica*** (Sande Lac.) Mitt. – **JJ** [Jeju-si, Musu stream, Goangryeong 2^nd^ Bridge, 18 Mar 2012, *Choi 120103* (JNU); Hong et al. 1961; Hattori et al. 1962a; Hong 1962a, 1966; Song and Yamada 2006], **JN** [Goheung-gun, Is. Oenarodo, Mt. Bongrae, valley, 20 May 2011, *Choi 110583* (JNU); Song and Yamada 2009b], **JB** [Buan-gun, Mt. Naebyen, Beadrock near road, 10 Mar 2009, *Choi 3390* (JNU)], **GB** [Hong 1962a, c, 1966; Choe 1980], **GW** [Gao and Chang 1983a, b; Kim and Hwang 1991; Kim et al. 1995], PB [Kim and Hwang 1991], **JG** [Kim and Hwang 1991], **YG** [Kim and Hwang 1991], **HN** [Kim and Hwang 1991], **HB** [Kim and Hwang 1991], **Korea** [Hattori 1967; Choe and Yamada 1974; Inoue 1976; Choe and Choi 1980; Miller et al. 1983; Hattori and Zhang 1985; Li 1985; Luo 1989; Hong 1997, 2003]

***Porella oblongifolia*** S.Hatt. – **JJ** [Jeju-si, Seogeomeun Oreum, 21 Jun 2011, *Choi 110880* (JNU); Hong 1966, Choe 1980, Song and Yamada 2006], **JB** [Hong 1966 as Porella oblongifolia var. takakii, Choe 1980], **GB** [Hong 1966 as Porella oblongifolia var. takakii, Choe 1980], **GW** [Jeongseon-gun, River Donggang, near ther Donggang, 17 Aug 2010, *Choi 7938* (JNU); Hong 1962a, b, 1966 as Porella oblongifolia var. takakii, Choe 1980], **JG** [Kim and Hwang 1991], **HB** [Kim and Hwang 1991], **Korea** [Hattori 1967; Iwatsuki and Mizutani 1972; Choe and Yamada 1974; Choe and Choi 1980; Gao and Chang 1981; Hattori and Zhang 1985; Luo 1989; Long and Grolle 1990; Hong 1997, 2003]

°***Porella perrottetiana*** (Mont.) Trevis. – **JN** [Hong 1966], **JB** [Choe 1980], **GB** [Hong 1966; Choe 1980], **Korea** [Hattori 1967, 1971, 1978; Choe and Yamada 1974; Choe and Choi 1980; Hattori and Zhang 1985; Hong 1997, 2003]

°***Porella platyphylla*** (L.) Pfeiff. – **PN** [Kim and Hwang 1991], **PB** [Kim and Hwang 1991], **HN** [Kim and Hwang 1991], **Korea** [Hong 1997, 2003]

°***Porella spinulosa*** (Steph.) S.Hatt. – **JJ** [Hattori 1944aPorella vernicosa fo. spinulosa], **JN** [Choe 1980, 1983], **GG** [Hattori 1970], **HN** [Kim and Hwang 1991], **Korea** [Hattori 1944aPorella vernicosa fo. spinulosa, Choe and Yamada 1974; Hattori 1978; Choe and Choi 1980; Aur and Zhang 1985; Hong 1997, 2003]

*Porella stephaniana (C.Massal.) S.Hatt. – **GW** [Yeongweol-gun, River Donggang, 17 Aug 2010, *Choi 7937* (JNU)]

***Porella subobtusa*** (Steph.) S.Hatt. – **JN** [Haenam-gun, Mt. Duryun, Taehengsa Temple valley, 18 May 2011, *Choi 110460* (JNU)], **GB** [Hong 1966 as Porella setigera var. subobtusa, Choe 1980], **GW** [Hong 1962a as Porella setigera var. subobtusa], **Korea** [Choe and Choi 1980; Hong 1997, 2003]

***Porella ulophylla*** (Steph.) S.Hatt. – **JJ** [Jeju-si, Mt. Halla, Seongpanak-Baekrokdam, 8 Aug 2010, *Choi 7708* (JNU); Hong and Kim 1961b; Hong et al. 1961; Hattori et al. 1962a,; Hong 1962a, 1966; Song and Yamada 2006 as *Macvicaria ulophylla*], **JN** [Goheung-gun, Is. Oenarodo, Mt. Bongrae, valley, 20 May 2011, *Choi 110594* (JNU); Hattori et al. 1962b; Hong 1962a, 1966; Song and Yamada 2009b], **JB** [Buan-gun, Mt. Naebyen, Beadrock near road, 10 Mar 2009, *Choi 3389* (JNU); Hong 1966], **GN** [Hapcheon-gun, Mt, Gaya, Baekwondong area, 8 Sep 2009, *Choi 4339* (JNU); Hattori et al. l962b; Hong 1966; Song and Yamada 2009a], **GB** [Hong 1966], **CN** [Horikawa 1934c as *Madotheca ulophylla*, Choe 1979 as *Macvicaria ulophylla*], **CB** [Yeongdong-gun, Mt. Minjuji, Forest lodge, 19 May 2012, *Choi 120589* (JNU); Hong 1962a, 1962d, 1966], **GG** [Hong 1960b, Hong and Kim 1961a, Hong 1962a, 1966], **GW** [Inje-gun, Mt. Seolak, Hangyeoryeong, 21 Sep 2009, *Choi 5053* (JNU); Hong 1962a, 1966; Kim et al. 1995], **HWN** [Kim and Hwang 1991], **HWB** [Gao and Chang 1983a, b as *Macvicaria ulophylla*], **PN** [Gao and Chang 1983a, b as *Macvicaria ulophylla*], **PB** [Kim and Hwang 1991], **JG** [Kim and Hwang 1991], **YG** [Kim and Hwang 1991], **HN** [Kim and Hwang 1991], **Korea** [Horikawa 1939 as *Madotheca ulophylla*, Hattori 1944a, Inoue 1955 as *Macvicaria ulophylla*, Kitagawa 1960 as *Macvicaria ulophylla*, Shin 1968 as *Macvicaria ulophylla*, Iwatsuki and Mizutani 1972 as *Macvicaria ulophylla*, Choe and Yamada 1974 as *Macvicaria ulophylla*, Hattori 1978 as *Macvicaria ulophylla*, Choe and Choi 1980 as *Macvicaria ulophylla*, Gao and Chang 1981, Inoue 1981, Aur and Zhang 1985 as *Macvicaria ulophylla*, Luo 1989 as *Macvicaria ulophylla*, Hong 1997, 2003 as *Macvicaria ulophylla*]

***Porella vernicosa*** Lindb. – **JJ** [Jeju-si, Musu stream, Goangryeong 2^nd^ Bridge, 18 Mar 2012, *Choi 120120* (JNU); Horikawa 1935a, 1935b as *Madotheca vernicosa*, Hattori 1950, Hong et al. 1961, Hattori et al. 1962a, Hong 1962a, 1966, Song and Yamada 2006], **JN** [Gurye-gun, Mt. Jiri, Nogodan, 29 Apr 2009, *Choi 3553* (JNU); Hong 1962a, 1966, Song and Yamada 2009b], **JB** [Muju-gun, Mt. Deogyu, Chilyeon valley, 17 Mar 2010, *Choi 7246* (JNU); Hong 1966], **GN** [Geoje-si, Mt. Noja, Forest lodge, 17 Mar 2011, *Choi 110038* (JNU); Hattori et al. 1962b, Hong 1966, Song and Yamada 2009a], **GB** [Cheongsong-gun, Mt. Juwang, Jeolgol, 9 Nov 2010, *Choi 8925* (JNU); Hong 1962c, 1966, Hattori 1970], **CN** [Gongju-si, Mt. Gyeryong, Temple Donghaksa valley, 8 Jul 2009, *Choi 4074* (JNU); Horikawa 1935a as *Madotheca vernicosa*, Ando 1960, Choe 1972], **CB** [Hong 1962a, 1962d, 1966], **GG** [Hong 1960b, c, 1962a, 1966; Hong and Kim 1961a], **GW** [Inje-gun, Mt. Seolak, Bongjeongam valley, 11 May 2011, *Choi 110199b* (JNU); Hong 1962a, 1966; Kim et al. 1995], **HWN** [Kim and Hwang 1991], **PN** [Kim and Hwang 1991], **PB** [Kim and Hwang 1991], **JG** [Kim and Hwang 1991], **YG** [Kim and Hwang 1991], **HN** [Kim and Hwang 1991], **HB** [Kim and Hwang 1991], **Korea** [Horikawa 1935b as *Madotheca vernicosa*, Inoue 1955; Ando 1960; Iwatsuki and Mizutani 1972; Choe and Yamada 1974; Choe and Choi 1980; Gao and Chang 1981; Aur and Zhang 1985; Luo 1989; Hong 1997, 2003]

***Preissia* Corda** (Marchantiaceae)

*Preissia quadrata (Scop.) Nees – **GW** [Jeongseon-gun, River Donggnag, Limstone, 7 Sep 2011, *Choi 110901* (JNU)]

***Protosolenostoma* (Amakawa) Bakalin et Vilnet** (Solenostomataceae)

***Protosolenostoma fusiforme*** (Steph.) Vilnet et Bakalin – **JJ** [Seogwipo-si, Bolre Oreum, elev. 1,230 m, 1 Nov 2011, *Choi 111411* (JNU); Stephani 1917b as *Solenostoma koreanum*; Amakawa 1960 as *Jungermannia koreana*, Hong 1966 as *Jungermannia koreana*, Váňa 1975 as *Solenostoma koreanum*, Choe 1980, 1983 as *Jungermannia fusiformis*, Song and Yamada 2006 as *Jungermannia fusiformis*], **JN** [Song and Yamada 2009b as *Jungermannia fusiformis*], **JB** [Muju-gun, Mt. Deogyu, *Choi 1039* (JNU)], **GN** [Gurye-gun, Mt. Jiri, Nogodan, 19 Sep 2009, *Choi 5042* (JNU); Song and Yamada 2009a as *Jungermannia fusiformis*], **Korea** [Iwatsuki and Mizutani 1972 as *Jungermannia koreana*, Choe and Yamada 1974 as *Jungermannia koreana*, Choe and Choi 1980 as *Jungermannia fusiformis*, Hong 1997, 2003 as *Jungermannia fusiformis*]

***Pseudolophozia* Konstant. et Vilnet** (Anastrophyllaceae)

***Pseudolophozia sudetica*** (Nees ex Huebener) Konstant. et Vilnet – **JJ** [Jeju-si, Seogeomeun Oreum, 20 Sep 2011, *Choi 111032b* (JNU)], **YG** [Kim and Hwang 1991 as *Lophozia alpestirs*], **Korea** [Hong 1997, 2003]

***Ptilidium* Nees** (Ptilidiaceae)

°***Ptilidium ciliare*** (L.) Hampe – **YG** [Kim and Hwang 1991], **HN** [Kim and Hwang 1991], HB [Kim and Hwang 1991], **Korea** [Aur and Zhang 1985; Hong 1997, 2003]

***Ptilidium pulcherrimum*** (Weber) Vain. – **JJ** [Jeju-si, Mt. Halla, Seongpanak-Baekrokdam, 8 Aug 2010, *Choi 7718* (JNU); Horikawa 1935a, 1936b; Hong and Kim 1961b; Hattori et al 1962a; Hong 1962a, 1966; Choe 1975, 1980, 1982, 1983; Song and Yamada 2006], **GN** [Hamyang-gun, Mt. Jiri, Cheonwnagbong, 29 Sep 2010, *Choi 8236* (JNU)], **GW** [Inje-gun, Mt. Seolak, Jungcheong, 21 Sep 2009, *Choi 5134* (JNU)], **YG** [Kim and Hwang 1991], **HN** [Kim and Hwang 1991], **HB** [Horikawa 1936b; Kim and Hwang 1991], **Korea** [Horikawa 1939; Iwatsuki and Mizutani 1972; Choe and Yamada 1974; Choe and Choi 1980; Inoue 1981; Aur and Zhang 1985; Hong 1997, 2003]

***Radula* Dumort.** (Radulaceae)

***Radula auriculata*** Steph. – **JJ** [Jeju-si, Mt. Halla, Seongpanak- Baekrokdam, 8 Aug 2010, *Choi 7714* (JNU); Hong and Kim 1961b as *Radula boryana*, Hattori et al. 1962a as *Radula boryana*, Hong 1962a, 1966 as *Radula boryana*, Song and Yamada 2006], **JN** [Yesu-gun, Mt. Geumo, Yulso-ri valley, 24 Feb 2010, *Choi 7142* (JNU); Hong 1962a, 1966 as *Radula boryana*, Song and Yamada 2009b], **JB** [Jangsu-gun, Mt. Jangan, Banghwadong valley, 30 Apr 2009, *Choi 3579* (JNU)], **GN** [Sancheong-gun, Mt. Jiri, below Cheonwangbong, 15 Jun 2009, *Choi 3800* (JNU); Hattori et al. l 962b as *Radula boryana*, Yamada 1979, Choe 1980,], **GB** [Hong 1966 as *Radula boryana*], **GG** [Hong 1962a as *Radula boryana*], **GW** [Inje-gun, Mt. Seolak, Hangyeoryeong, 21 Sep 2009, *Choi 5093* (JNU); Hong 1962a as *Radula boryana*, Hong 1962b, Kim and Hwang 1991, Kim et al. 1995], **HN** [Kim and Hwang 1991], **Korea** [Hattori 1975a; Choe and Yamada 1974; Yamada 1979; Choe 1980; Choe and Choi 1980; Hong 1997, 2003]

*Radula brunnea Steph. – **JJ** [Seogwipo-si, Mt. Halla, Baekrokdam, 7 Aug 2012, *Choi 120814a* (JNU)]

***Radula cavifolia*** Hampe ex Gottsche – **JJ** [Horikawa 1935a, Hattori 1944a, Hong 1966, Choe 1980, Song and Yamada 2006], **JB** [Muju-gun, Mt. Deogyu, 30 Oct 2008, *Choi 11107*; Choe 1980], **GN** [Hapcheon-gun, Mt, Gaya, Sangwangbong, 8 Sep 2009, *Choi 4362* (JNU)], **GW** [Taebaek-si, Mt. Taebaek, Banjae area, 16 Sep 2009, *Choi 4522* (JNU); Kim and Hwang 1991, Kim et al. 1995], **Korea** [Hong 1962a; Iwatsuki and Mizutani 1972; Choe and Yamada 1974; Yamada 1979; Choe and Choi 1980; Miller et al. 1983; Hong 1997, 2003]

***Radula complanata*** (L.) Dumort. – **JN** [Wando-gun, Is. Bogil, ridge, 16 Nov 2010, *Choi 9017* (JNU)], **JB** [Buan-gun, Mt. Naebyen, Jikso Waterfall, 10 Mar 2009, *Choi 3300* (JNU); Hong 1966, Choe 1980, 1983], **Korea** [Choe and Choi 1980; Hong 1997, 2003]

***Radula constricta*** Steph. – **JJ** [Jeju-si, Tamla valley, 25 Sep 2012, *Choi 121005* (JNU); Hattori et al. 1962a, Hong 1962a, Song and Yamada 2006], **JN** [Gurye-gun, Mt. Jiri, Nogodan, 29 Apr 2009, *Choi 3551* (JNU); Hong 1962a, 1966, Song and Yamada 2009b], **JB** [Yamada 1979], **GN** [Hamcheon-gun, Mt. Hwangmae, ridge, 3 Aug 2010, *Choi 7504* (JNU); Hattori et al. 1962b; Hong 1966; Song and Yamada 2009a], **GB** [Ulleung-gun, Naribunji, 21 Oct 2010, *Choi 8771* (JNU); Hong 1966], **CB** [Hong 1962a, d, 1966], **GG** [Hong 1962a, 1966], **GW** [Inje-gun, Mt. Seolak, Hangyeoryeong, 21 Sep 2009, *Choi 4330* (JNU); Hong 1962a, 1966; Kim and Hwang 1991; Kim et al. 1995], **PN** [Gao and Chang 1983a, b; Kim and Hwang 1991], **PB** [Kim and Hwang 1991], **HN** [Kim and Hwang 1991], **HB** [Kim and Hwang 1991], **Korea** [Inoue 1981; Li 1985; Long and Grolle 1990; Choe and Yamada 1974; Choe and Choi 1980; Hong 1997, 2003]

°***Radula fauriana*** Steph. – **GW** [Kim and Hwang 1991; Kim et al. 1995], **YG** [Yamada 1989], HN [Kim and Hwang 1991], **Korea** [Hong 1997, 2003]

***Radula japonica*** Gottsche – **JJ** [Jeju-si, Che Oruem, 27 Aug 2010, *Choi 8069* (JNU); Hong 1962a, 1966, Song and Yamada 2006], **JN** [Goheung-gun, Is. Oenarodo, Mt. Bongrae, valley, 20 May 2011, *Choi 110581* (JNU); Hong 1966; Yamada 1979; Song and Yamada 2009b], **JB** [Buan-gun, Mt. Naebyen, Beadrock near road, 10 Mar 2009, *Choi 3381* (JNU); Hong 1966], **GN** [Namhae-gun, Mt. Geum, valley, 21 May 2011, *Choi 110634b* (JNU); Hattori et al. 1962b, Hong 1966], **GB** [Cheongsong-gun, Mt. Juwang, Jabang waterfall, 8 Nov 2010, *Choi 8901* (JNU); Hong 1962a, c, 1966], **CN** [Choe 1972, 1979], CB [Hong 1962a, d], **GG** [Hong 1962a, 1966], **GW** [Hong 1962a, 1966; Kim and Hwang 1991; Kim et al. 1995], **HWN** [Kim and Hwang 1991], **PB** [Kim and Hwang 1991], **YG** [Kim and Hwang 1991], **HN** [Kim and Hwang 1991], **HB** [Kim and Hwang 1991], **Korea** [Reimers 1931; Hattori 1944a; Iwatsuki and Mizutani 1972; Choe and Yamada 1974; Inoue 1976; Choe and Choi 1980; Miller et al. 1983; Hong 1997, 2003]

***Radula kojana*** Steph. – **JJ** [Jeju-si, Seongpanak area, 19 Jun 2011, *Choi 110677* (JNU); Horikawa 1934c; Hattori 1944a; Hattori 1953; Hattori et al. 1962a; Hong 1962a, 1966; Chen and Wu 1964; Choe 1980, 1983; Song and Yamada 2006], **JN** [Haenam-gun, Mt. Duryun, Duryunsa Temple, 23 Apr 2010, *Choi 7322* (JNU)], **JB** [Buan-gun, Mt. Naebyen, Jikso Waterfall, 10 Mar 2009, *Choi 3301* (JNU)], **CN** [Yamada 1979], **Korea** [Choe and Yamada 1974; Choe and Choi 1980; Hong 1997, 2003]

***Radula obtusiloba*** Steph. – **JJ** [Jeju-si, Gwangryeongcheon stream, 4 May 2012, *Choi 120507* (JNU); Hong 1962a; Song and Yamada 2006], **JN** [Hong 1966; Song and Yamada 2009b], GN [Hattori et al. 1962b; Choe 1980], **GB** [Cheongsong-gun, Mt. Juwang, The 2^nd^ waterfall, 8 Nov 2010, *Choi 8918* (JNU)], **GW** [Inje-gun, Mt. Seolak, Kkeucheong, 21 Sep 2009, *Choi 5103* (JNU); Hong 1962a, b, 1966; Yamada 1979; Choe 1980; Kim et al. 1995], **YG** [Yamada 1989], **HN** [Kim and Hwang 1991], **HB** [Kim and Hwang 1991], **Korea** [Aur and Zhang 1985; Choe and Yamada 1974; Choe and Choi 1980; Hong 1997, 2003]

***Radula oyamensis*** Steph. – **JJ** [Seogwipo-si, Suak valley, 11 Oct 2012, *Choi 121054* (JNU)], **JN** [Sinan-gun, Is. Gageodo The 2^nd^ village-lighthouse 20 Apr 2012, *Choi 121210* (JNU); Song and Yamada 2009b], **GN** [Song and Yamada 2009a].

°***Radula perrottetii*** Gottsche – **GW** [Yamada and Choe 1997], **JG** [Kim and Hwang 1991], HB [Kim and Hwang 1991], **Korea** [Hong 1997, 2003]

***Radula tokiensis*** Steph. – **JJ** [Seogwipo-si, Dongheung-dong valley 15 Oct 2012, *Choi 121124* (JNU); Hong 1966; Song and Yamada 2006], **JN** [Haenam-gun, Mt. Duryun, Taehengsa Temple valley, 18 May 2011, *Choi 110471* (JNU); Hong 1966], **JB** [Buan-gun, Mt. Naebyen, Namyeochi, 10 Mar 2009, *Choi 3339* (JNU); Hong 1966], **GN** [Geoje-si, Oryung reservoir, 16 Mar 2011, *Choi 110020* (JNU); Hong 1966], **GB** [Hong 1966], **CN** [Gongju-si, Mt. Gyeryong, Temple Donghaksa valley, 8 Jul 2009, *Choi 4075* (JNU); Yamada 1979; Choe 1980], **CB** [Yamada 1979], **GG** [Hong 1962a, 1966], **GW** [Hong 1966], **Korea** [Choe and Yamada 1974; Choe and Choi 1980; Hong 1997, 2003]

***Reboulia* Raddi** (Aytoniaceae)

**Reboulia hemisphaerica subsp. hemisphaerica** (L.) Raddi – **JJ** [Jeju-si, Tamla valley, 10 Apr 2012, *Choi 120327* (JNU); Horikawa 1934d], **JN** [Jangheung-gun, Mt. Suin, valley, 24 Jun 2009, *Choi 4038* (JNU)], **JB** [Buan-gun, Mt. Naebyen, Beadrock near road, 10 Mar 2009, *Choi 3386* (JNU)], **GN** [Sancheong-gun, Mt. Jiri, Beopgyesa Temple, 14 Jun 2009, *Choi 3709* (JNU); Hattori et al. 1962b; Choe 1980], **GB** [Choe 1980], **CN** [Choe 1972, 1979, 1980], **GG** [Hong 1960b; Hong and Kim 1961a; Hong 1962a], **GW** [Jeongseon-gun, River Donggang, near ther Donggang, 16 Aug 2010, *Choi 7909* (JNU); Hong and Kim 1960, Hong 1962a, Kim et al. 1995], **HWN** [Gao and Chang 1983a, 1983b, Kim and Hwang 1991], **PN** [Horikawa 1934d; Kim and Hwang 1991], **PB** [Kim and Hwang 1991], **JG** [Kim and Hwang 1991], **YG** [Kim and Hwang 1991], **HN** [Kim and Hwang 1991], **HB** [Kim and Hwang 1991], **Korea** [Horikawa 1939; Hattori 1944a; Shin1968, 1970; Hattori 1971; Inoue 1976; Choe and Yamada 1974; Choe and Choi 1980; Miller et al. 1983; Hong 1997]

**Reboulia hemisphaerica subsp. orientalis** R.M.Schust. – **JJ** [Jeju-si, Nokkome Oreum, 28 Oct 2010, *Choi 8806* (JNU); Song and Yamada 2006], **JN** [Song and Yamada 2009b], **JB** [Buan-gun, Mt. Naebyeon, Jikso waterfall, 20 Apr 2010, *Choi 7295* (JNU)], **GB** [Cheongsong-gun, Mt. Juwang, Jabang waterfall, 8 Nov 2010, *Choi 8903* (JNU)], **GG** [Choe 1980], **GW** [Jeongseon-gun, River Donggang, near ther Donggang, 17 Aug 2010, *Choi 7932* (JNU)], **Korea** [Hong 2003]

***Riccardia* Gray** (Aneuraceae)

*Riccardia aeruginosa Furuki – **JB** [Jeongeup-si, Mt. Naejang, 15 May 2011, *Choi 110413* (JNU)]

***Riccardia chamedryfolia*** (With.) Grolle – **JJ** [Choe 1980, Song and Yamada 2006], **JN** [Suncheon-si, Mt. Jogyeo, Seonamsa Temple, 7 Dec 2010, *Choi 9111* (JNU)], **GN** [Choe 1980], **GB** [Ulsan-si, Mt. Jeongjok, Mujechi 1 neup, 30 Sep 2010, *Choi 8308* (JNU)], **Korea** [Choe and Choi 1980; Hong 1997, 2003]

*Riccardia glauca Furuki – **JJ** [Seogwipo-si, Hyodon stream, 7 Aug 2010, *Choi 7631* (JNU)]

°***Riccardia latifrons*** (Lindb.) Lindb. – **JG** [Kim and Hwang 1991], **YG** [Kim and Hwang 1991], **HN** [Kim and Hwang 1991], **HB** [Kim and Hwang 1991], **Korea** [Hong 1997, 2003]

**Riccardia multifida subsp. decrescens** (Steph.) Furuki – **JN** [Haenam-gun, Mt. Duryun, Taehengsa Temple valley, 18 May 2011, *Choi 110466* (JNU)], **JB** [Muju-gun, Mt. Deogyu, Osujagul Cave, 14 Apr 2009, *Choi 3448* (JNU)], **GW** [Kim et al. 1995 as *Riccardia multifida*], **PB** [Kim and Hwang 1991 as *Riccardia multifida*], **JG** [Kim and Hwang 1991 as *Riccardia multifida*], **YG** [Gao and Chang 1983a, 1983b as *Riccardia multifida*], **HB** [Kim and Hwang 1991 as *Riccardia multifida*], **Korea** [Hong 1997 as *Riccarida multifida*, Hong 2003]

*Riccardia nagasakiensis (Steph.) S.Hatt. – **JJ** [Jeju-si, Mt. Halla, Tamla valley, 25 Sep 2012, *Choi 6042* (JNU)]

***Riccardia palmata*** (Hedw.) Carruth. – **GN** [Hamyang-gun, Mt. Jiri, Chilseon valley, *Choi 8287* (JNU)], **GW** [Pyeochang-gun, Mt. Odae, Jinbugogye, 29 Aug 2009, *Choi 4289* (JNU)], **YG** [Kim and Hwang 1991], **HN** [Kim and Hwang 1991], **HB** [Kim and Hwang 1991], **Korea** [Hong 1997, 2003]

*Riccardia planiflora (Steph.) S.Hatt. – **JJ** [Jeju-si, Mt. Halla, Tamla valley, 25 Sep 2012, *Choi 121000* (JNU)], **JN** [Wando-gun, Sangwhangbong, valley, 9 Feb 2010, *Choi 7010* (JNU)], **GW** [Inje-gun, Mt. Seolak, Baekdamsa Temple, 28 Aug 2009, *Choi 5277* (JNU)]

***Riccia* L.** (Ricciaceae)

*Riccia beyrichiana Hampe – **JJ** [Jeju-si, Gujwa-eup, Gimnyeong-ri, Manjangul lava tube, 18 Jan 2020, *Choi 201063* (JNU)]

*Riccia bifurca Hoffm. – **JJ** [Jeju-si, Gujwa-eup, Gimnyeong-ri, near Manjangul lava tube, 18 Jan 2020, *Choi 201065b* (JNU)]

***Riccia fluitans*** L. – **JJ** [Jeju-si, *Cryptomeria japonica* forest, 25 Aug 2010, *Choi 8009* (JNU)], **CN** [Choe 1975, 1979], **GN** [Horikawa 1934d], **PN** [Gao and Chang 1983a, b; Kim and Hwang 1991], **Korea** [Horikawa 1939; Hattori 1943b; Shin 1968, 1970; Hong 1962a; Choe and Yamada 1974; Choe and Choi 1980; Gao and Chang 1981; Miller et al. 1983; Hong 1997, 2003]

***Riccia glauca*** L. – **JJ** [Jeju-si, Che Oruem, 27 Aug 2010, *Choi 8060* (JNU); Choe 1980, Song and Yamada 2006], **JN** [Hwasun-gun, *Psilotun nudum* area, 1 Aug 2009, *Choi 4203* (JNU)], **GB** [Choe 1975, 1980], **CN** [Choe 1975, 1979, 1980, 1983 as Riccia glauca var. subinermis], **GW** [Choe 1983 as Riccia glauca var. subinermis, Gao and Chang 1983a, 1983b, Kim and Hwang 1991, Kim et al. 1995], **HWN** [Kim and Hwang 1991], **PN** [Kim and Hwang 1991], **PB** [Kim and Hwang 1991], **HB** [Kim and Hwang 1991], **Korea** [Horikawa 1939; Hattori 1943b; Hong 1962a; Shin 1970; Choe and Yamada 1974; Inoue 1976; Choe and Choi 1980 as Riccia glauca var. subinermis, Gao and Chang 1981; Miller et al. 1983; Hong 1997, 2003]

***Riccia huebeneriana*** Lindenb. – **JJ** [Jeju-si, *Cryptomeria japonica* forest, 25 Aug 2010, *Choi 8007* (JNU)], **GB** [Ulleung-gun, Seonginbong, 20 Oct 2010, *Choi 8768* (JNU)], **CN** [Choe 1975, 1979, 1980, 1983], **HWN** [Gao and Chang 1983a, b; Kim and Hwang 1991], **PN** [Kim and Hwang 1991], **PB** [Kim and Hwang 1991], **HB** [Kim and Hwang 1991], **Korea** [Choe and Choi 1980; Hong 1997, 2003]

°***Riccia pseudofluitans*** C.Gao et G.C.Zhang – **YG** [Choe 1983; Park and Choi 2007], **Korea** [Choe and Choi 1980; Hong 1997, 2003]

***Riccia sorocarpa*** Bisch. – **GN** [Choe 1975, 1980, 1983], **GB** [Ulleung-gun, Seonginbong, 20 Oct 2010, *Choi 8769* (JNU)], **Korea** [Choe and Choi 1980; Hong 1997, 2003]

***Ricciocarpos* Corda** (Ricciaceae)

***Ricciocarpos natans*** (L.) Corda – **JJ** [Jeju-si, Aewol-eup, Hagamot, 21 Nov 2009, *Choi 5243* (JNU)], **CN** [Choe 1975, 1977, 1979, 1980, 1983], **Korea** [Choe and Choi 1980; Gao and Chang 1981; Aur and Zhang 1985; Hong 1997, 2003]

***Scapania* (Dumort.) Dumort.** (Scapaniaceae)

***Scapania ampliata*** Steph. – **JJ** [Jeju-si, Mt. Halla, Jindalraebat, 9 Jul 2012, *Choi 120702* (JNU); Hong and Kim 1961b; Hattori et al. 1962a; Hong 1962a, 1966; Choe 1980; Song and Yamada 2006], **JN** [Hong 1966], **JB** [Muju-gun, Mt. Deogyu, 18 Sep 2008, *Choi 11060* (JNU); Hong 1966; Choe 1980], **GN** [Geochang-gun, Mt. Namdeogyu, top of mountain, 11 Nov 2010, *Choi 8956* (JNU)], **GG** [Hong 1966, Choe 1980], **GW** [Inje-gun, Mt. Seolak, Hangyeoryeong, 21 Sep 2009, *Choi 5072* (JNU); Kim et al. 1995], **PB** [Kim and Hwang 1991], **HN** [Kim and Hwang 1991], **Korea** [Iwatsuki and Mizutani 1972; Choe and Yamada 1974; Inoue 1974; Choe and Choi 1980; Hong 1997, 2003]

***Scapania apiculata*** Spruce – **JN** [Gurye-gun, Mt. Jiri, Nogodan, 19 Sep 2009, *Choi 5038* (JNU)], **GW** [Taebaek-si, Mt. Taebaek, Janggunbong, 15 Sep 2009, *Choi 4460* (JNU)], **YG** [Gao and Chang 1983a, b], **HN** [Kim and Hwang 1991], **Korea** [Hong 1997, 2003]

°***Scapania carinthiaca*** J.B. Jack ex Lindb. – **GW** [Kim et al. 1995], **YG** [Gao and Chang 1983a, 1983b], **HN** [Kim and Hwang 1991], **HB** [Kim and Hwang 1991], **Korea** [Hong 1997, 2003]

***Scapania ciliata*** Sande Lac. – **JJ** [Jeju-si, Tamla valley, 25 Sep 2012, *Choi 120973b* (JNU); Horikawa 1934c as *Scapania spinosa*, Hattori et al. 1962a as *Scapania spinosa*, Hong 1962a, 1966 as *Scapania spinosa*, Choe 1980, Song and Yamada 2006], **JB** [Namwon-si, Mt. Jiri, Simwon valley, 20 Jun 2009, *Choi 3963* (JNU); Choe 1980], **GN** [Hamyang-gun, Mt. Jiri, Cheonwnagbong, 28 Sep 2010, *Choi 9134* (JNU)], **GB** [Ulleung-gun, Seonginbong, 20 Oct 2010, *Choi 8750* (JNU); Hong 1966 as *Scapania spinosa*, Choe 1980], **CN** [Choe 1972 as *Scapania spinosa*], **GW** [Inje-gun, Mt. Seolak, Kkeucheong, 21 Sep 2009, *Choi 5116* (JNU)], **PB** [Kim and Hwang 1991], **HN** [Kim and Hwang 1991], **Korea** [Horikawa 1939 as *Scapania spinosa*, Hattori 1944a as *Scapania spinosa*, Amakawa and Hattori 1954 as *Scapania spinosa*, Amakawa 1967; Shin 1968, 1970; Iwatsuki and Mizutani 1972; Choe and Yamada 1974; Inoue 1974, 1981; Choe and Choi 1980; Li 1985 as *Scapania spinosa*, Long and Grolle 1990; Hong 1997, 2003]

***Scapania curta*** (Mart.) Dumort. – **JJ** [Hattori et al. 1962a, Hong 1962a, Song and Yamada 2006], **JN** [Hong 1962a, 1966; Song and Yamada 2009b], **GN** [Hattori et al. 1962b; Choe 1980], **GG** [Hong 1966; Choe 1980], **GW** [Hwacheon-gun, Sangseo-myeon, Guun-ri, 2 Sep 2012, *Kim Hwacheon-6* (JNU, KB)], **Korea** [Choe and Yamada 1974; Choe and Choi 1980; Hong 1997, 2003]

***Scapania integerrima*** Steph. – **JJ** [Jeju-si, Musu stream, Goangryeong 2^nd^ Bridge, 17 Mar 2012, *Choi 120074c* (JNU); Hong 1966 as *Scapania stephanii*, Song and Yamada 2006 as *Scapania ligulata*], **JN** [Goheung-gun, Mt. Palyeoung, Forest lodge valley, 23 Jun 2009, *Choi 4024* (JNU); Hong 1966 as *Scapania stephanii*, Song and Yamada 2009b as *Scapania ligulata*], **JB** [Buan-gun, Mt. Naebyen, Beadrock near road, 10 Mar 2009, *Choi 3355* (JNU); Hong 1966 as *Scapania stephanii*], **GN** [Geochang-gun, Mt. Namdeogyu, top of mountain, 11 Nov 2010, *Choi 8968* (JNU)], **GB** [Hong 1966 as *Scapania stephanii*], **CN** [Gongju-si, Mt. Gyeryong, Temple Donghaksa valley, 8 Jul 2009, *Choi 4087* (JNU); Choe 1979 as *Scapania stephanii*], **GG** [Hong 1962a, 1966 as *Scapania stephanii*, Choe 1980, 1983], **GW** [Inje-gun, Mt. Seolak, Bongjeongam valley, 11 May 2011, *Choi 110168* (JNU); Hong 1962a as *Scapania stephanii*, Kim and Hwang 1991 as *Scapania stephanii*, Kim et al. 1995 as *Scapania stephanii*], **Korea** [Choe and Yamada 1974 as *Scapania stephanii*, Choe and Choi 1980 as *Scapania stephanii*, Hong 1997, 2003 as *Scapania stephanii*]

***Scapania irrigua*** (Nees) Nees – **JJ** [Jeju-si, Mt. Halla, Seongpanak- Baekrokdam, 8 Aug 2010, *Choi 7736* (JNU); Song and Yamada 2006], **JN** [Haenam-gun, Mt. Duryun, Taehengsa Temple valley, 18 May 2011, *Choi 110501* (JNU)], **GN** [Sancheong-gun, Mt. Jiri, Jungbong area, 4 Oct 2011, *Choi 111162* (JNU)], **GW** [Inje-gun, Mt. Seolak, Jungcheongbong stony field, 12 May 2011, *Choi 110296* (JNU)], **YG** [Kim and Hwang 1991], **HN** [Kim and Hwang 1991], **HB** [Kim and Hwang 1991], **Korea** [Hong 1997, 2003]

***Scapania paludosa*** (Müll.Frib.) Müll.Frib. – **GN** [Hamyang-gun, Mt. Jiri, Chilseon valley, 28 Sep 2010, *Choi 8209* (JNU)], **GW** [Kim et al. 1995], **Korea** [Hong 1997, 2003]

***Scapania parvidens*** Steph. – **JJ** [Jeju-si, Gyoraeri stream, 11 Apr 2012, *Choi 120365b* (JNU)], **JN** [Sinan-gun, Is. Gageodo, Bolryemi seashore 22 Apr 2012, *Choi 120396* (JNU)], GB [Hong 1962a as Scapania parvitexta var. minor], **GW** [Inje-gun, Mt. Seolak, Baekdamsam Temple valley, 11 May 2011, *Choi 110153* (JNU); Kim and Hwang 1991, Kim et al. 1995], **Korea** [Hong 1997, 2003]

***Scapania parvitexta*** Steph. – **JJ** [Jeju-si, Musu stream, Goangryeong 2^nd^ Bridge, 18 Mar 2012, *Choi 120164* (JNU)], **JN** [Sinan-gun, Is. Gageodo, The 2^nd^ village, 20 Apr 2012, *Choi 120389* (JNU)], **JB** [Muju-gun, Mt. Deogyu, 1 Jul 2008, *Choi 10911* (JNU)], **GN** [Geochang-gun, Mt. Namdeogyu, top of mountain, 11 Nov 2010, *Choi 8973* (JNU)], **CN** [Nonsan-si, Mt. Daedun, Surak valley, 31 Mar 2009, *Choi 3419* (JNU)], GG [Hong 1962a], **GW** [Inje-gun, Mt. Seolak, Hangyeoryeong, 21 Sep 2009, *Choi 5070* (JNU); Hong and Kim 1960, Hong 1962a, Choe 1980, Kim et al. 1995], **YG** [Kim and Hwang 1991], **HN** [Kim and Hwang 1991], **Korea** [Iwatsuki and Mizutani 1972; Choe and Yamada 1974; Choe and Choi 1980; Hong 1997, 2003]

*Scapania scandica (Arnell et H.Buch) Macvicar – **GW** [Inje-gun, Mt. Seolak, Socheong stony field, 12 May 2011, *Choi 110268* (JNU)]

*Scapania sphaerifera H.Buch et Tuom – **GW** [Inje-gun, Mt. Seolak, Jungcheongbong stony field, 12 May 2011, *Choi 110308* (JNU)]

*Scapania subalpina (Nees ex Lindenb.) Dumort. – **GW** [Inje-gun, Mt. Seolak, Baekdamsa Temple, 28 Aug 2009, *Choi 5277* (JNU)]

***Scapania undulata*** (L.) Dumort. – **JJ** [Seogwipo-si, Bolrae Oreum, 5 Sep 2012, *Choi 120720* (JNU); Amakawa and Hattori 1953, Song and Yamada 2006], **JN** [Gurye-gun, Mt. Jiri, Nogodan, 29 Apr 2009, *Choi 3555*; Hong 1966, Song and Yamada 2009b], **JB** [Buan-gun, Mt. Naebyen, Beadrock near road, 10 Mar 2009, *Choi 3388* (JNU)], **GN** [Hamyang-gun, Mt. Jiri, Chilseon valley, 28 Sep 2010, *Choi 8118* (JNU); Hattori et al. 1962b, Choe 1980], **GB** [Yeongju-si, Mt. Sobaek, Birobong area, 2 Sep 2009, *Choi 4303* (JNU); Hong 1966, Choe 1980], **CN** [Gongju-si, Mt. Gyeryong, Temple Donghaksa valley, 8 Jul 2009, *Choi 4097* (JNU); Choe 1980], **GG** [Hong 1960b, 1962a, 1966; Choe 1980], **GW** [Inje-gun, Mt. Seolak, Socheong shelter valley, 12 May 2011, *Choi 110345* (JNU); Nakai 1918 as *Scapania dentata*, Hong and Kim 1960; Hong 1962a, 1966; Choe 1980; Kim et al. 1995], **YG** [Kim and Hwang 1991], **HN** [Kim and Hwang 1991], **HB** [Kim and Hwang 1991], **Korea** [Iwatsuki and Mizutani 1972; Inoue 1974; Choe and Yamada 1974; Schuster 1974; Choe and Choi 1980; Hong 1997, 2003]

***Schistochilopsis* (N.Kitag.) Konstant.** (Lophoziaceae)

***Schistochilopsis cornuta*** (Steph.) Konstant. – **JJ** [Jeju-si, Mt. Halla, Baekrokdam, 8 Aug 2010, *Choi 7769* (JNU); Hattori et al. 1962a as *Lophozia cornuta*, Hong 1962a, 1966 as *Lophozia cornuta*, Choe 1975, 1980, 1983 as *Lophozia cornuta*, Song and Yamada 2006 as *Lophozia cornuta*], **GN** [Geochang-gun, Mt. Namdeogyu, top of mountain, 11 Nov 2010, *Choi 8966* (JNU)], **GW** [Inje-gun, Mt. Seolak, Hangyeoryeong, 21 Sep 2009, *Choi 5069* (JNU); Horikawa 1936a as *Lophozia undulata*, Gao and Chang 1983a, 1983b as *Lophozia cornuta*, Kim et al. 1995 as *Lophozia cornuta*], **JG** [Kim and Hwang 1991 as *Lophozia cornuta*], **YG** [Kim and Hwang 1991 as *Lophozia cornuta*], **HN** [Kim and Hwang 1991 as *Lophozia cornuta*], **HB** [Kim and Hwang 1991 as *Lophozia cornuta*], **Korea** [Horikawa 1939 as *Lophozia undulata*, Kitagawa 1965b as *Lophozia cornuta*, Iwatsuki and Mizutani 1972 as *Lophozia cornuta*, Choe and Yamada 1974 as *Lophozia cornuta*, Inoue 1974 as *Lophozia cornuta*, Choe and Choi 1980 as *Lophozia cornuta*, Hong 1997, 2003 as *Lophozia cornuta*]

°***Schistochilopsis incisa*** (Schrad.) Konstant. – **GW** [Gao and Chang 1983a, b as *Lophozia incisa*, Kim et al. 1995 as *Lophozia incisa*], **PN** [Gao and Chang 1983a, b as *Lophozia incisa*], **PB** [Kim and Hwang 1991 as *Lophozia incisa*], **YG** [Kim and Hwang 1991 as *Lophozia incisa*], **HN** [Kim and Hwang 1991 as *Lophozia incisa*], **HB** [Kim and Hwang 1991 as *Lophozia incisa*], **Korea** [Kitagawa 1965b as *Lophozia incisa*, Schuster 1969 as *Lophozia incisa*, Iwatsuki and Mizutani 1972 as *Lophozia incisa*, Choe and Yamada 1974 as *Lophozia incisa*, Hattori 1975a as *Lophozia incisa*, Choe and Choi 1980 as *Lophozia incisa*, Aur and Zhang 1985 as *Lophozia incisa*, Li 1985 as *Lophozia incisa*, Hong 1997, 2003 as *Lophozia incisa*]

***Solenostoma* Mitt.** (Solenostomataceae)

*Solenostoma bilobum (Amakawa) Potemkin et Nyushko – **JJ** [Seogwipo-si, Seondol valley, 18 Sep 2011, *Choi 110919* (JNU)], **JB** [Muju-gun, Mt. Deogyu, elev. 700 m, *Choi 3593* (JNU)], **GN** [Sancheong-gun, Mt. Jiri, below Cheonwangbong, 15 Jun 2009, *Choi 3762* (JNU)]

*Solenostoma cyclops (S.Hatt.) R.M.Schust. – **GN** [Hamyang-gun, Mt. Jiri, Chilseon valley, 28 Sep 2010, *Choi 8204* (JNU)]

***Solenostoma faurieanum*** (Beauverd) R.M.Schust. – **JJ** [Quelpaert (=Jeju Island) 1906 *U. Faurie* (*No. 106*) *G00115348* (Holotype; G); Stephani 1917c as *Jungermannia decurrens*, Amakawa 1960 as *Jungermannia fauriana*, Hong 1962a, 1966 as *Jungermannia fauriana*, Choe 1980, 1983 as *Jungermannia fauriana*, Song and Yamada 2006 as *Jungermannia fauriana*], **Korea** [Choe and Yamada 1974 as *Jungermannia fauriana*, Choe and Choi 1980 as *Jungermannia fauriana*, Hong 1997, 2003 as *Jungermannia fauriana*]

*Solenostoma jirisanense Bakalin et S.S. Choi – **GN** [Mt. Jiri, elev. 1,620 m, *Choi 3747-3* (Holotype: JNU)]

*Solenostoma minutissimum (Amakawa) Bakalin – **JJ** [Seogwipo-si, Bolre Oreum, elev. 1,230 m, 5 Sep 2012, *Choi 120745* (JNU)]

*Solenostoma obscurum (A. Evans) R.M.Schust. – **GW** [Inje-gun, Mt. Seolak, Socheong shelter valley, elev. 1,407 m, 12 May 2011, *Choi 110353* (JNU)]

*Solenostoma purpuratum (Mitt.) Steph. var. ***koponenii*** Bakalin et Li Wei – **JJ** [Goangryeongcheon stream, elev. 766 m, 14 May 2012, *Choi 120375* (JNU)]

***Solenostoma pyriflorum*** Steph. – **JJ** [Seogwipo-si, Dulle route, elev. 600–800 m, *Bakalin Kor-29-66-15* (VBGI); Hattori et al. 1962a as *Jungermannia pyriflora*, Choe 1980 as *Jungermannia pyriflora*, Song and Yamada 2006 as *Jungermannia pyriflora*], **GB** [Ulleung-gun, Ulleung-eup, Seonginbong, 977 m, 20 Oct 2010, *Choi 8724* (JNU); Hong 1962a, 1962c, 1966 as *Jungermannia pyriflora*], **CN** [Choe 1980 as *Jungermannia pyriflora*], **GW** [Kim et al. 1995 as *Jungermannia pyriflora*, Choe 1980 as *Jungermannia pyriflora*], **YG** [Kim and Hwang 1991 as *Jungermannia pyriflora*], **Korea** [Choe and Yamada 1974 as *Jungermannia pyriflora*, Choe and Choi 1980 as *Jungermannia pyriflora*, Váňa and Inoue 1983 as *Jungermannia pyriflora*, Long and Grolle 1990 as *Jungermannia pyriflora*, Hong 1997, 2003 as *Jungermannia pyriflora*]

***Solenostoma rotundatum*** Amakawa – **JJ** [Seogwipo-si, Donnaeko stream, elev. 172 m, 31 Oct 2011, *Choi 111381* (JNU); Hong 1966 as *Jungermannia harana*, Song and Yamada 2006 as *Jungermannia rotundata*], **JN** [Goheung-gun, Is. Oenarodo, Mt. Bongrae, elev. 120 m, 20 May 2011, *Choi 110580* (JNU); Hong 1966 as *Jungermannia harana*, Song and Yamada 2009b as *Jungermannia rotundata*], **JB** [Muju-gun, Mt. Deogyu, 27 Jun 2008, *Choi 865* (JNU); Hong 1966 as *Jungermannia harana*], **CN** [Gyeoyong-si, Mt. Gyeryong, elev. 290 m, *Choi 4091* (JNU)], **GN** [Hapcheon-gun, Mt. Gaya, elev. 585 m, *Choi 4355* (JNU)], **GB** [Youngju-si, Mt. Sobaek, elev. 569 m, *Choi 4304* (JNU); Hong 1966 as *Jungermannia harana*], **GG** [Hong 1962a, 1966 as *Jungermannia harana*], **GW** [Jeongseon-gun, Mt. Haembaek, *Choi 4147* (JNU)], **Korea** [Choe and Yamada 1974 as *Jungermannia harana*, Choe and Choi 1980 as *Jungermannia harana*, Hong 1997, 2003 as *Jungermannia rotundata*]

***Solenostoma sunii*** Bakalin et Vilnet – **JJ** [Seogwipo-si, Bolre Oreum, elev. 1,230 m, *Choi 111425* (JNU)], **JB** [Muju-gun, Mt. Drogyu, 30 Jun 2008, *Choi 878* (JNU)] **GN** [Sancheong-gun, Mt. Jiri, Jangsanri valley, elev. 848 m, 13 Jun 2009, *Choi 3653* (JNU); Choe 1983 as Jungermannia pyriflora var. major], **CN** [Choe 1980, 1983 as as Jungermannia pyriflora var. major], **GG** [Hong 1962a, 1966 as Jungermannia pyriflora var. major], **GW** [Hong 1962a as Jungermannia pyriflora var. major], **Korea** [Choe and Choi 1980 as Jungermannia pyriflora var. major, Hong 1997 as Jungermannia pyriflora var. major]

***Sphenolobopsis* R.M.Schust. et N.Kitag.** (Anastrophyllaceae)

*Sphenolobopsis pearsonii (Spruce) R.M.Schust. – **GW** [Sokcho-si, Mt. Seolak, 13 May 2011, *Choi 110394* (JNU)]

***Sphenolobus* (Lindb.) Berggr.** (Anastrophyllaceae)

***Sphenolobus minutus*** (Schreb. ex D.Crantz) Berggr. – **GW** [Inje-gun, Mt. Seolak, Jungcheong, 21 Sep 2009, *Choi 5143* (JNU); Kim et al. 1995 as *Anastrophyllum minutum*], **PB** [Kim and Hwang 1991 as *Anastrophyllum minutum*], **JG** [Kim and Hwang 1991 as *Anastrophyllum minutum*], **YG** [Kim and Hwang 1991 as *Anastrophyllum minutum*], **HN** [Kim and Hwang 1991 as *Anastrophyllum minutum*], **HB** [Kim and Hwang 1991 as *Anastrophyllum minutum*], **Korea** [Hong 1997, 2003 as *Anastrophyllum minutum*]

***Sphenolobus saxicola*** (Schrad.) Steph. – **GN** [Hamyang-gun, Mt. Jiri, Chilseon valley, 28 Sep 2010, *Choi 8170* (JNU)], **GW** [Inje-gun, Mt. Seolak, Jungcheong, 21 Sep 2009, *Choi 5134* (JNU)], **HN** [Kim and Hwang 1991 as *Anastrophyllum saxicola*], **HB** [Kim and Hwang 1991 as *Anastrophyllum saxicola*], **Korea** [Hong 1997, 2003 as *Anastrophyllum saxicola*]

***Spruceanthus* Verd.** (Lejeuneaceae)

*Spruceanthus kiushianus (Horik.) X.Q.Shi, R.L.Zhu et Gradst. – **JJ** [Jeju-si, Hancheon Bridge, 14 Mar 2012, *Choi 120008* (JNU)], **GN** [Geoje-si, Oryung reservoir, 16 Mar 2011, *Choi 110009* (JNU)]

*Spruceanthus semirepandus (Nees) Verd. – **JN** [Sinan-gun, Is. Gageodo Mt. Doksil 2 Mar 2010, *Choi 7188* (JNU)]

***Syzygiella* Spruce** (Adelanthaceae)

***Syzygiella autumnalis*** (DC.) K.Feldberg, Váňa, Hentschel et Heinrichs – **JJ** [Jeju-si, Musu stream, Goangryeong 2^nd^ Bridge, 18 Mar 2012, *Choi 120128* (JNU); Hong and Kim 1961b as *Jamesoniella autumnalis*, Hattori et al. 1962a as *Jamesoniella autumnalis*, Song and Yamada 2006 as *Jamesoniella autumnalis*], **JN** [Gurye-gun, Mt. Jiri, Nogodan, 29 Apr 2009, *Choi 3561* (JNU); Hong 1962a, 1966 as *Jamesoniella autumnalis*, Song and Yamada 2009b as *Jamesoniella autumnalis*], **JB** [Musu-gun, Mt. Deogyu, Hyangjeokbong area, 10 Oct 2009, *Choi 5280* (JNU)], **GN** [Hamyang-gun, Mt. Jiri, Chilseon valley, 28 Sep 2010, *Choi 8147* (JNU); Hattori et al. 1962b as *Jamesoniella autumnalis*, Song and Yamada 2009a as *Jamesoniella autumnalis*], **GB** [Ulleung-gun, Seonginbong, 20 Oct 2010, *Choi 8729* (JNU); Hong 1962c, 1966 as *Jamesoniella autumnalis*], **CN** [Nonsan-si, Mt. Daedun, Surak valley, 31 Mar 2009, *Choi 3415* (JNU); Choe 1979 as *Jamesoniella autumnalis*], **GG** [Hong 1962a, 1966 as *Jamesoniella autumnalis*], **GW** [Inje-gun, Mt. Seolak, Hangyeoryeong, 28 Aug 2009, *Choi 4262* (JNU); Hong 1962a, 1966 as *Jamesoniella autumnalis*, Kim and Hwang 1991 as *Jamesoniella autumnalis*, Kim et al. 1995 as *Jamesoniella autumnalis*], **Korea** [Reimers 1931 as *Jamesoniella autumnalis*, Amakawa 1959 as *Jamesoniella autumnalis*, Choe and Yamada 1974 as *Jamesoniella autumnalis*, Inoue 1976, 1981 as *Jamesoniella autumnalis*, Choe and Choi 1980 as *Jamesoniella autumnalis*, Aur and Zhang 1985 as *Jamesoniella autumnalis*, Hong 1997, 2003 as *Jamesoniella autumnalis*]

***Syzygiella nipponica*** (S.Hatt.) K.Feldberg, Váňa, Hentschel et Heinrichs – **JJ** [Seogwipo-si, Yeongsil valley, 12 Oct 2012, *Choi 121062* (JNU)]

***Targionia* L.** (Targioniaceae)

***Targionia hypophylla*** L. – **GW** [Jeongseon-gun, River Donggang, near ther Donggang, 17 Aug 2010, *Choi 7961* (JNU)], **Korea** [Hattori 1948; Hattori and Mizutani 1969; Hong 1962a, 1997, 2003; Choe and Yamada 1974; Choe and Choi 1980; Choe 1980, 1983; Miller et al. 1983]

***Tetralophozia* (R.M.Schust.) Schljakov** (Anastrophyllaceae)

*Tetralophozia filiformis (Steph.) Urmi – **GN** [Hamyang-gun, Mt. Jiri, Chilseon valley, 28 Sep 2010, *Choi 8162* (JNU)], **GW** [Inje-gun, Mt. Seolak, Bongjeongam valley, 11 May 2011, *Choi 110169* (JNU)]

***Trichocolea* Dumort.** (Trichocoleaceae)

***Trichocolea tomentella*** (Ehrh.) Dumort. – **JJ** [Hong 1966, Choe 1975, 1980, 1983], **GN** [Hamyang-gun, Mt. Jiri, Hansin valley, 7 Oct 2009, *Choi 6036* (JNU)], **JG** [Kim and Hwang 1991], **Korea** [Shin 1968, 1970; Iwatsuki and Mizutani 1972; Choe and Yamada 1974; Hattori 1975a; Choe and Choi 1980; Miller et al. 1983; Aur and Zhang 1985; Hong 1997, 2003]

***Trichocoleopsis* S.Okamura** (Neotrichocoleaceae)

***Trichocoleopsis sacculata*** (Mitt.) S.Okamura – **JJ** [Jeju-si, Gwangryeongcheon stream, 4 May 2012, *Choi 120509* (JNU); Hattori 1944a, Hattori et al 1962a; Hong 1966; Choe 1975, 1980; Song and Yamada 2006], **JN** [Gurye-gun, Mt. Jiri, Nogodan, 29 Apr 2009, *Choi 3524* (JNU); Hong 1962a, 1966; Song and Yamada 2009b], **JB** [Jangsu-gun, Mt. Jangan, Banghwadong valley, 30 Apr 2009, *Choi 3579* (JNU)], **GN** [Hamyang-gun, Mt. Jiri, Chilseon valley, 28 Sep 2010, *Choi 8137* (JNU); Hong and Yoo 1961; Hattori et al. 1962b; Hong 1962a, 1966; Choe 1975, 1980], **GB** [Cheongsong-gun, Mt. Juwang, The 2^nd^ waterfall, 8 Nov 2010, *Choi 8913* (JNU)], **CN** [Choe 1979], **GG** [Hong 1962a, 1966, Choe 1975, 1980], **GW** [Inje-gun, Mt. Seolak, Gugokdam valley, 14 Oct 2010, *Choi 8647* (JNU); Horikawa 1951a, Hong and Kim 1960, Hong 1962a, 1966, Choe 1975, 1980, Kim et al. 1995], **PN** [Kim and Hwang 1991], **PB** [Kim and Hwang 1991], **JG** [Kim and Hwang 1991], **YG** [Kim and Hwang 1991], **HN** [Gao and Chang 1983a, 1983b], **Korea** [Stephani 1909*as Ptilidium sacculatum*, Reimers 1931 as *Ptilidium sacculatum*, Horikawa 1934a, 1955; Hattori 1944a; Iwatsuki and Mizutani 1972; Choe and Yamada 1974; Inoue 1974; Choe and Choi 1980; Hong 1997, 2003]

***Trilophozia* (R.M.Schust.) Bakalin** (Lophoziaceae)

***Trilophozia quinquedentata*** (Huds.) Bakalin – **GB** [Choe 1980, 1983 as *Tritomaria quinquedentata*], **GW** [Inje-gun, Mt. Seolak, Baekdamsam Temple valley, *Choi 110156* (JNU)], **PB** [Kim and Hwang 1991 as *Tritomaria quinquedentata*], **JG** [Kim and Hwang 1991 as *Tritomaria quinquedentata*], **YG** [Kim and Hwang 1991 as *Tritomaria quinquedentata*], **HN** [Kim and Hwang 1991 as *Tritomaria quinquedentata*], **HB** [Kim and Hwang 1991 as *Tritomaria quinquedentata*], **Korea** [Choe and Choi 1980 as *Tritomaria quinquedentata*, Hong 1997, 2003 as *Tritomaria quinquedentata*]

***Tritomaria* Schiffn. ex Loeske** (Lophoziaceae)

***Tritomaria exsecta*** (Schmidel) Schiffn. ex Loeske – **JJ** [Jeju-si, Mt. Halla, Baekrokdam, 8 Aug 2010, *Choi 7749* (JNU)], **JN** [Hong 1962a, 1966; Choe 1983; Song and Yamada 2009b], **JB** [Namwon-si, Mt. Jiri, Simwon valley, 20 Jun 2009, *Choi 3999* (JNU)], GN [Hamyang-gun, Mt. Jiri, Cheonwnagbong, 29 Sep 2010, *Choi 8251* (JNU); Hong and Yoo 1961, Choe 1975, 1980], **GW** [Inje-gun, Mt. Seolak, Hangyeoryeong, 21 Sep 2009, *Choi 5084* (JNU); Kim et al. 1995], **PB** [Kim and Hwang 1991], **JG** [Kim and Hwang 1991], **YG** [Kim and Hwang 1991], **HN** [Kim and Hwang 1991], **HB** [Kim and Hwang 1991], **Korea** [Hattori 1975a; Choe and Yamada 1974; Choe and Choi 1980; Hong 1997, 2003]

*Tritomaria koreana Bakalin, S.S.Choi et B.Y.Sun – **GN** [Hamyang-gun, Mt. Jiri, Cheonwnagbong, 29 Sep 2010, *Choi 8225* (JNU)]

***Tuzibeanthus* S.Hatt.** (Lejeuneaceae)

***Tuzibeanthus chinensis*** (Steph.) Mizut. – **GB** [Cheongsong-gun, Mt. Juwang, Jabang waterfall, 8 Nov 2010, *Choi 8905* (JNU)], **GW** [Hong 1962a, 1966; Choe 1980, 1983], **Korea** [Choe and Yamada 1974; Choe and Choi 1980; Hong 1997, 2003]

***Wiesnerella* Schiffn.** (Wiesnerellaceae)

***Wiesnerella denudata*** (Mitt.) Steph. – **JJ** [Jeju-si, Musu stream, 28 Oct 2010, *Choi 8833* (JNU); Horikawa 1934b; Miller et al. 1983; Hong 1962a; Song and Yamada 2006], **Korea** [Hattori 1944a; Iwatsuki and Mizutani 1972; Choe and Yamada 1974; Inoue 1976; Choe and Choi 1980; Choe 1983; Hong 1997, 2003]

***Xenochila* R.M.Schust.** (Delavayellaceae)

***Xenochila integrifolia*** (Mitt.) Inoue – **JB** [Muju-gun, Mt. Deogyu, 2 Apr 2008, *Choi 10302* (JNU)], **GN** [Song and Yamada 2009a], **GB** [Yeongju-si, Mt. Sobaek, Birobong area, 2 Sep 2009, *Choi 4316* (JNU)], **GW** [Taebaek-si, Mt. Taebaek, Janggunbong, 15 Sep 2009, *Choi 4448* (JNU)], **HWN** [Kim and Hwang 1991], **JG** [Kim and Hwang 1991], **Korea** [Iwatsuki and Mizutani 1972; Inoue 1974; Choe and Yamada 1974; Choe 1980; Choe and Choi 1980; Long and Grolle 1990; Hong 1997, 2003]

### Excluded and doubtful records

***Bazzania bidentula*** (Steph.) Yasuda – This species was recorded as for **JJ** [Hong 1962a], **GW** [Hong 1962a], **Korea** [Reimers 1931 as *Mastiogbryum bidentulum* Steph.]; however, all records of this taxon from temperate East Asia should belong to *B. parabidentula* (Bakalin 2016a).

***Bazzania flaccida*** (Dumort.) Grolle – This species was recorded for **Korea** [Hong 2003], however, we were not able to find the references to literature and the location of vouchers; we exclude this species from the checklist.

***Calycularia crispula*** Mitt. – This species was recorded for **GN** [Hattori et al. l962b], **JG** [Kim and Hwang 1991], **Korea** [Iwatsuki and Mizutani 1972], however, as it was shown by Konstantinova and Mamontov (2010) all reports of the species from the Russian Far East, Korea and Japan belong to *C. laxa*.

***Calypogeia azurea*** Stotler et Crotz – This species was recorded for **JJ** [Hong 1966 as *Calypogeia trichomanis*, Choe 1975, 1980 as *Calypogeia trichomanis*, Song and Yamada 2006], **JN** [Hong 1962a, 1966 as *Calypogeia trichomanis*, Song and Yamada 2009b], **GN** [Choe 1975, 1980 as *Calypogeia trichomanis*], **PB** [Kim and Hwang 1991 as *Calypogeia trichomanis*], **YG** [Kim and Hwang 1991 as *Calypogeia trichomanis*], **HN** [Kim and Hwang 1991 as *Calypogeia trichomanis*], **HB** [Kim and Hwang 1991 as *Calypogeia trichomanis*], **Korea** [Choe and Yamada 1974 as *Calypogeia trichomanis*, Choe and Choi 1980 as *Calypogeia trichomanis*, Hong 1997, 2003], however, as it was recently shown, all reports of this species from temperate East Asia refer to *Calypogea orientalis* Buczkowska & Bakalin (Buczkowska et al. 2018).

***Calypogeia granulata*** Inoue – This species was recorded for **Korea** [Park and Choi 2007], however, we were not able to find the references to literature and the location of vouchers; we exclude this species from the checklist.

***Cephalozia hamatiloba*** Steph. – This species was recorded for **Korea** [Park and Choi 2007], however, we were not able to find the references to literature and the location of vouchers; we exclude this species from the checklist. Also, this species is a southerly distributed taxon stretching to Indochina.

***Cololejeunea latilobula*** (Herzog) Tixier. – This species was recorded for **Korea** [Hong 1997, 2003], however, we were not able to find the references to literature and the location of vouchers; we exclude this species from the checklist.

***Conocephalum conicum*** (L.) Dumort. – This species was recorded for **JJ** [Horikawa 1935a], **JN** [Hong 1962a], **GG** [Hong 1962a], **GW** [Hong 1962a], **All the provinces** [Choe 1980, Kim and Hwang 1991], however, this is mainly a European species (Szweykowski et al. 2005), whereas all reports from East Asia belong to *Conocephalum salebrosum* Szweyk., Buczk. et Odrzyk.

***Delavayella serrata*** Steph. – This species was recorded for **Korea** [Park and Choi 2007], however, we were not able to find the references to literature and the location of vouchers; we exclude this species from the checklist.

***Diplophyllum obtusifolium*** (Hook) Dumort. – This species was recorded for **GW** [Kim and Hwang 1991], however, records of this taxon from North Asia should belong to *Diplophyllum sibiricum* Vilnet et Bakalin (Bakalin and Vilnet 2018).

**Fossombronia foveolata var. cristula** (Austin) R.M.Schust. – This species was recorded for **Korea** [Hong 1997], however, all reports of this species from temperate East Asia referable to *Fossombronia japonica* Schiffn. (Borovichev and Bakalin 2017).

***Fossombronia cristula*** Austin – This species was recorded for **Korea** [Choe 1983], however, all reports of this species from temperate East Asia refer to *Fossombronia japonica* Schiffn. (Borovichev and Bakalin 2017)

***Frullania bolanderi*** Austin – This species was recorded for **GW** [Kim and Hwang 1991], **HN** [Kim and Hwang 1991], **HB** [Kim and Hwang 1991], **Korea** [Choe 1980; Hong 1997, 2003], however, this is an endemic to North America (Mamontov et al. 2020), whereas all reports from East Asia belong to *Frullania austinii* J.J.Atwood, Vilnet, Mamontov et Konstant.

***Gongylanthus ericetorum*** (Raddi) Nees –This species was recorded for **Korea** [Park and Choi 2007], however, we were not able to find the references to literature and the location of vouchers; we exclude this species from the checklist.

***Gymnomitrion concinnatum*** (Lightf.) Corda – This species was recorded for **YG** [Kim and Hwang 1991], **PN** [Kim and Hwang 1991], **Korea** [Hong 1997, 2003], however, all reports of this species from temperate East Asia refer to *Gymnomitrion faurianum* (Steph.) Horik. (Bakalin 2016b)

***Gymnomitrion corallioides*** Nees – This species was recorded for **JJ** [Choe 1980, 1982, 1983; Song and Yamada 2006], **Korea** [Choe and Choi 1980; Hong 1997, 2003], however, all reports of this species from temperate East Asia refer to *Gymnomitrion faurianum* (Steph.) Horik. (Bakalin 2016b)

***Gymnomitrion revolutum*** (Nees) H.Philib – This species was recorded for **YG** [Kim and Hwang 1991 as *Marsupella revoluta*], **Korea** [Hong 1997, 2003 as *Marsupella revoluta*]. Not confirmed. The records from Korea probably belong to *Gymnomitrion parvitextum* (Steph.) Mamontov, Konstant. et Potemkin.

***Jungermannia cordifolia*** Hook. nom. nud. – This species was recorded for **HWN** [Kim and Hwang 1991], however, the records from Korea are erroneous and probably belong to *Jungermannia exsertifolia* Steph.

***Lejeunea cavifolia*** (Ehrh.) Lindb. – This species was recorded from **GW** [Kim et al. 1995], **PB** [Kim and Hwang 1991], **YG** [Gao and Chang 1983a, b; Kim and Hwang 1991], HN [Kim and Hwang 1991], **HB** [Kim and Hwang 1991], **Korea** [Reimers 1931; Choe and Choi 1980; Hong 1997, 2003]. However, as it was shown by M. Mizutani (1961)*L. cavifolia* was reported for many times in Japan, but all or nearly all reports are belonging to *L. japonica*. Almost the same situation applies to this species in the Russian Far East: although it was many times reported, but all reports are based on *L. japonica* (Bakalin 2019). Within the Korean Peninsula we checked many specimens and we did not find ‘true’ *L. cavifolia*, which is probably limited to Europe and North America.

***Lepidozia filamentosa*** (Lehm. et Lindenb.) Lehm. et Lindenb. – This species was recorded for **GW** [Hong 1962a], however, if the narrow species treatment would be adopted in the group, this name should be referred to *L. subtransversa* in East Asia, whereas ‘true’ *L. filamentosa* occurs in western North America

***Lopholejeunea subfusca*** (Nees) Schiffn. – This species was recorded for **JN** [Hong 1962a], **Korea** [Hong 1966, 1997, 2003; Choe and Yamada 1974], however, the distribution of *L. subfusca* covers tropical and subtropical areas, so its occurrence in Mt. Jiri of Jeollanam-do province is doubtful. The plants most probably belong to *Acanthocoleus yoshinagana* (S. Hatt.) Mizut., which is relatively common on Mt. Jiri, but it was not recorded by Hong (1962a).

***Marchantia pinnata*** Steph. – This species was recorded for **Korea** [Park and Choi 2007], however, we were not able to find the references to literature and the location of vouchers; we exclude this species from the checklist.

***Marsupella emarginata*** (Ehrh.) Dumort. – This species was recorded for **PB** [Kim and Hwang 1991 as], **HN** [Kim and Hwang 1991], **HB** [Kim and Hwang 1991], however, the records from Korea are erroneous and probably belong to *Marsupella tubulosa* Steph.

***Marsupella sphacelata*** (Giesecke ex Lindenb.) Dumort. – This species was recorded for **GW** [Kim and Hwang 1991], **YG** [Kim and Hwang 1991], **Korea** [Hong 2003], however, the records from Korea are erroneous and probably belong to *Marsupella apertifolia* Steph.

***Metalejeunea cucullata*** (Reinw., Blume et Nees) Grolle – This species was recorded for **Korea** [Park and Choi 2007], however, we were not able to find the references to literature and the location of vouchers; we exclude this species from the checklist.

***Metzgeria consanguinea*** Schiffn. – This species was recorded for **JG** [Kim and Hwang 1991], **YG** [Kim and Hwang 1991], **HN** [Gao and Chang 1983a, b], **Korea** [Shin 1970; Iwatsuki and Mizutani 1972; Choe and Yamada 1974; Choe 1975, 1980; Choe and Choi 1980; Inoue 1981; Hong 1997], however, the records from Korea are misidentified *Metzgeria temperata* (Kuwahara 1976)

***Odontoschisma grosseverrucosum*** Steph. – This species was recorded for **GW** [Kim and Hwang 1991], however, the record from Korea is erroneous and probably belongs to *Odontoschisma pseudogrosseverrucosum* Gradst., Aranda et Vanderp.

***Plagiochasma intermedium*** Lindenb. et Gottsche – This species was recorded for **GG** [Hong 1962a], **Korea** [Choe and Yamada 1974], however, the report refers to *Plagiochasma pterospermum* C. Massal.

***Plaiochila delavayi*** Steph. – This species was recorded for **Korea** [Reimers 1931, Park and Choi 2007], however, the records from Korea and Japan are doubtful and probably belong to *Plagiochila ovalifolia* Mitt. (Hattori 1949; So 2001).

***Plectocolea flagellata*** S.Hatt. This species was recorded for **GG** [Hong 1966, 1997, 2003 as *Jungermannia flagellata* (S.Hatt.) Amakawa]. Not confirmed. The species is a Japanese temperate lowland endemic (Yakushima Island, Kyushu), and may be easily recognized due to its common ventral leafless geotropic stolons and botryoidal oil-bodies (Bakalin et al. 2020a [Solenostomataceae]).

***Plectocolea hyalina*** (Lyell) Mitt. – This species was recorded for **CB** [Choe 1980, 1983 as *Jungermannia hyalina* Lyell], **GG** [Hong 1966, 1997, 2003 as *Jungermannia hyalina*], **GW** [Kim et al. 1995 as *Jungermannia hyalina*], **JN** [Bakalin 2014], however, the plants most probably belong to *Metasolenostoma ochotense* and *Plectocolea infusca* (Bakalin et al. 2020a [Solenostomataceae]).

***Plectocolea otiana*** S.Hatt. – This species was recorded for **GG** [Hong 1962a as *Jungermannia otiana*], **GW** [Choe and Yamada 1974, 1962a as *Jungermannia otiana*]. Not confirmed. The species is a Japanese temperate lowland endemic and may be easily recognized due to its monoicous inflorescence (Bakalin et al. 2020a [Solenostomataceae]).

***Plectocolea horikawana*** Amakawa – This species was recorded for **JB** [Choi et al. 2012c as *Solenostoma horikawanum*], however, the species is based on misidentification of weakly developed, prostrate modification of *Plectocolea virgata* (Bakalin et al. 2020a [Solenostomataceae]).

***Porella pinnata*** L. – This species was recorded for **GW** [Kim et al. 1995], **JG** [Kim and Hwang 1991], **HN** [Kim and Hwang 1991], however, the distribution of this species is European and hardly occurred in East Asia.

***Radula boryana*** (F.Weber) Nees ex Mont. – This species was recorded for **JJ** [Hong 1962a], **JN** [Hong 1962a], **GG** [Hong 1962a], **GW** [Hong 1962a]; however, there reports belong to *R. auriculata* or *R. chinensis* (Yamada 1979), whereas true *R. boroyana* is an African taxon.

***Radula chinensis*** Steph. – This species was recorded for **Korea** [Park and Choi 2007], however, we were not able to find the references to literature and the location of vouchers; we exclude this species from the checklist.

***Riccardia incurvata*** Lindb. – This species was recorded for **HWN** [Kim and Hwang 1991], **HB** [Kim and Hwang 1991], however the distribution of these three species is European-North American and hardly occurred in East Asia.

***Riccia frostii*** Austin – This species was recorded for **Korea** [Amnokgang River: Park and Choi 2007], however, we were not able to find the references to literature and the location of vouchers; we exclude this species from the checklist.

***Riccia nipponica*** S.Hatt. – This species was recorded for **Korea** [Park and Choi 2007], however, we were not able to find the references to literature and the location of vouchers; we exclude this species from the checklist.

***Sandeothallus japonicus*** (Inoue) Crand.-Stotl. et Stotler – This species was recorded for **Korea** [Park and Choi 2007 as *Moerckia japonica* Inoue], however, we were not able to find the references to literature and the location of vouchers; we exclude this species from the checklist.

***Scapania ligulata*** Steph. – This species was recorded for **Korea** [Park and Choi 2007 as *Scapania stephanii* Müll.Frib.], however, we identified as *S. parvitexta*, *S. intergerrima* and *S. parvides* in Korean specimens. The taxonomy of Scapania sect. Stephanii is quite controversial; we checked all available materials of *Scapania* in Korea and found *S. parvitexta*, *S. intergerrima* and *S. parvidens*, but not *S. ligulata*.

***Solenostoma plagiochilaceum*** (Grolle) Váňa et D.G.Long – This species was recorded for **Korea** [Park and Choi 2007 as *Jungermannia plagiochilacea* Grolle], however, we were not able to find the references to literature and the location of vouchers; we exclude this species from the checklist.

### Synonyms

*Anastrophyllum minutum* (Schreb. ex D.Crantz) R.M.Schust. ≡ *Sphenolobus minutus*

*Anastrophyllum reichardtii* (Gottsche) Steph. = *Anastrophyllum assimile*

*Anastrophyllum saxicola* (Schrad.) R.M.Schust ≡ *Sphenolobus saxicola*

*Anthoceros koreanus* Steph. = *Phaeoceros carolinianus*

*Anthoceros laevis* L. ≡ *Phaeoceros laevis*

*Apometzgeria pubescens* (Schrank) Kuwah. ≡ *Metzgeria pubescens*

*Archilejeunea kiushiana* (Horik.) Verd. ≡ *Spruceanthus kiushianus*

*Athalamia nana* (Shimizu et S.Hatt.) S.Hatt. ≡ *Clevea nana*

*Asterella chichibuensis* Shimizu et S.Hatt. = *Asterella cruciata*

*Asterella gracilis* (EWeber) Underw. ≡ *Mannia gracilis*

*Asterella koreana* (Horik.) Horik. ≡ *Asterella leptophylla*

*Asterella ludwigii* (Schwägr.) Underw. = *Mannia gracilis*

*Asterella odora* S.Hatt. = *Asterella cruciata*

*Barbilophozia attenuata* (Mart.) Loeske ≡ *Neoorthocaulis attenuatus*

*Barbilophozia gracilis* (Schleich.) Müll.Frib. ≡ *Neoorthocaulis attenuatus*

*Bazzania albicans* Steph. = *Bazzania tridens*

Bazzania denudata subsp. ovifolia (Steph.) S.Hatt. = *Bazzania denudata*

*Bazzania ovifolia* (Steph.) S.Hatt. = *Bazzania denudata*

*Brachiolejeunea sandvicensis* (Gottsche) A.Evans ≡ *Acrolejeunea sandvicensis*

*Calypogeia trichomanes* (L.) Corda nom. rej. = *Calypogeia azurea*

Cephalozia bicuspidata subsp. otaruensis (Steph.) S.Hatt. ≡ *Cephalozia otaruensis*

*Cephalozia catenulata* (Huebener) Lindb. ≡ *Fuscocephaloziopsis catenulata*

*Cephalozia nipponica* S.Hatt. ≡ Fuscocephaloziopsis catenulata subsp. nipponica

Cephalozia catenulata subsp. nipponica (S.Hatt.) Inoue ≡ Fuscocephaloziopsis catenulata subsp. nipponica

*Cephalozia leucantha* Spruce ≡ *Fuscocephaloziopsis leucantha*

*Cephalozia lunulifolia* (Dumort.) Dumort. ≡ *Fuscocephaloziopsis lunulifolia*

*Cephaloziella byssacea* (Roth.) Warnst. = *Cephaloziella divaricata*

*Cephaloziella echinata* S.Hatt. = *Cephaloziella spinicaulis*

*Cephaloziella jishibae* (Steph.) Horik. ≡ *Odontoschisma jishibae*

*Cephaloziella revurvifolia* Steph. ≡ *Cylindrocolea recurvifolia*

*Cephaloziella subdentata* Warnst. = *Cephaloziella spinigera*

*Chandonanthus birmensis* Steph. ≡ *Plicanthus birmensis*

*Chandonanthus hirtellus* (F.Weber) Mitt. ≡ *Plicanthus hirtellus*

*Cheilolejeunea imbricata* (Nees) S.Hatt. = *Cheilolejeunea trapezia*

*Cololejeunea aoshimensis* (Horik.) S.Hatt. = *Cololejeunea planissima*

*Cololejeunea goebelii* (Schiffn.) Schiffn. = *Cololejeunea trichomanis*

*Cololejeunea minuta* (Mitt.) Steph. = *Cololejeunea longifolia*

*Cololejeunea minutissima* (Sm.) Schiffn. = *Myriocoleopsis minutissima*

*Conocephalum supradecompositum* (Lindb.) Steph. = *Conocephalum japonicum*

*Dicranolejeunea yoshinagana* (S.Hatt.) Mizut. ≡ *Acanthocoleus yoshinaganus*

*Diplophyllum plcatum* Lindb. ≡ *Douinia plicata*

*Euosmolejeunea auriculata* Steph. = *Lejeunea compacta*

*Fimbriaria koreana* Horik. = *Asterella leptophylla*

Frullania brittoniae subsp. truncatifolia (Steph.) R.M.Schust. et S.Hatt. = *Frullania muscicola*

*Frullania clavellata* Mitt. = *Frullania appendiculata*

*Frullania delavayi* Steph. = *Frullania inflexa*

*Frullania jackii* Gottsche = *Frullania davurica*

Frullania jackii Gottsche subsp. japonica (Sande Lac.) S.Hatt = *Frullania davurica*

*Frullania japonica* Sande Lac. = *Frullania davurica*

*Frullania koreana* Steph. = *Frullania hamatiloba*

*Frullania mayebarae* S.Hatt. = *Frullania inflata*

*Frullania moniliata* (Reinw., Blume et Nees) Mont. auct. = *Frullania appendiculata*

Frullania moniliata subsp. obscura Verd. = *Frullania appendiculata*

Frullania muscicola var. inuena (Steph.) Kamim. = *Frullania muscicola*

Frullania nepalensis var. nishiyamensis (Steph) S.Hatt. = *Frullania nepalensis*

*Frullania nishiyamensis* Steph = *Frullania nepalensis*

Frullania osumiensis var. orbiculata Kamim. = *Frullania osumiensis*

*Frullania squarrosa* (Reinw., Blume et Nees) Dumort. = *Frullania ericoides*

Frullania tamarisci subsp. monilata (Reinw., Blume et Nees) Kamin. = *Frullania appendiculata*

Frullania tamarisci subsp. obscura (Verd.) S.Hatt. = *Frullania appendiculata*

Herbertus hutchinsiae subsp. schusteri H.A.Mill. et Scott. = *Herbertus aduncus*

*Herbertus pusillus* (Steph.) S.Hatt. = *Herbertus aduncus*

*Heteroscyphus bescherellei* (Steph.) S.Hatt. = *Heteroscyphus coalitus*

*Iwatsukia jishibae* (Steph.) N.Kitag. ≡ *Odontoschisma jishibae*

*Jamesoniella autumnalis* (DC.) Steph. ≡ *Syzygiella autumnalis*

*Jubula japonica* Steph. ≡ Jubula hutchinsiae subsp. japonica

*Jungermannia amakawana* Grolle = *Liochlaena subulata*

*Jungermannia comamta* Ness ≡ *Plectocolea comata*

Jungermannia cordifolia subsp. excertifolia (Steph.) Amakawa ≡ *Jungermannia exsertifolia*

*Jungermannia cylindrica* (Steph.) S.Hatt. = *Liochlaena subulata*

*Jungermannia decurrens* Steph. = *Solenostoma faurianum*

*Jungermannia erecta* (Amakawa) Amakawa ≡ *Plectocolea erecta*

*Jungermannia fauriana* Beauverd ≡ *Solenostoma faurianum*

*Jungermannia fusiformis* (Steph.) Steph. ≡ *Protosolenostoma fusiforme*

*Jungermannia harana* (Amakawa) Amakawa = *Solenostoma rotundatum*

*Jungermannia koreana* (Steph.) Amakawa = *Protosolenostoma fusiforme*

*Jungermannia infusca* (Mitt.) Steph. ≡ Plectocolea infusca var. infusca

Jungermannia infusca var. ovalifolia (Amakawa) Amakawa ≡ *Plectocolea ovalifolia*

Jungermannia infusca var. ovicalyx (Steph.) Amakawa auct.= Plectocolea infusca var. recondita

Jungermannia lanceolata subsp. stephanii Amakawa = *Liochlaena subulata*

*Jungermannia radicellosa* (Mitt.) Steph. ≡ *Plectocolea radicellosa*

*Jungermannia rotundata* (Amakawa) Amakawa ≡ *Solenostoma rotundatum*

*Jungermannia polyanthos* L. ≡ *Chiloscyphus polyanthos*

*Jungermannia pyriflora* Steph. ≡ *Solenostoma pyriflorum*

Jungermannia pyriflora var. major (S.Hatt.) Amakawa = *Solenostoma sunii*

*Jungermannia subulata* Evans ≡ *Liochlaena subulata*

*Jungermannia tristis* Nees = *Jungermannia atrovirens*

*Jungermannia truncata* Nees ≡ *Plectocolea truncata*

*Jungermannia tsukushinensis* (Amakawa) Amakawa = *Plectocolea truncata*

*Jungermannia virgata* (Mitt.) Steph. ≡ *Plectocolea virgata*

*Leicolea heterocolpos* (Thed. ex Hartm.) H.Buch ≡ *Mesoptychia heterocolpos*

*Leiocolea mayebarae* (S.Hatt.) Furuki et Mizut. = *Mesoptychia mayebarae*

*Lejeunea auriculata* (Steph.) S.Hatt. = *Lejeunea compacta*

*Lejeunea claviflora* (Steph.) S.Hatt. = *Lejeunea neelgherriana*

*Lejeunea rotundistipula* (Steph.) S.Hatt. = *Lejeunea parva*

*Lejeunea vaginata* Steph. = *Lejeunea discreta*

*Lejeunea ulicina* (Taylor) Gottsche, Lindenb. et Nees = *Microlejeunea ulicina*

*Lepidozia coreana* Steph. = *Lepidozia subransversa*

*Leptocolea longilobula* Horik. = *Cololejeunea raduliloba*

*Lophocolea compacta* Mitt. ≡ *Cryptolophocolea compacta*

*Lophocolea cuspidata* (Nees) Limpr. ≡ *Lophocolea bidentata*

*Lophozia alpestirs* (Schleich.) A.Evans nom. rej. = *Pseudolophozia sudetica*

*Lophozia cornuta* (Steph.) S.Hatt. ≡ *Schistochilopsis cornuta*

*Lophozia heterocolpos* (Thed. in Hartm.) Howe ≡ *Mesoptychia heterocolpos*

*Lophozia incisa* (Schrad.) Dumort. ≡ *Schistochilopsis incisa*

*Lophozia porphyroleuca* (Nees) Schiffn. auct. non = *Lophozia guttulata*

*Lophozia fauriana* Steph. = *Lophozia guttulata*

*Lophozia longiflora* (Nees) Schiffn. = *Lophozia guttulata*

*Lophozia mayebarae* (S.Hatt.) N.Kitag. = *Mesoptychia mayebarae*

*Lophozia undulata* Horik. = *Schistochilopsis cornuta*

*Madotheca setigera* (Steph.) S.Hatt. = Porella caespitans var. cordifolia

*Madotheca tosana* Steph. ≡ Porella acutifolia subsp. tosana

*Madotheca ulophylla* Steph. ≡ *Porella ulophylla*

*Madotheca vernicosa* (Lindb.) Steph. ≡ *Porella vernicosa*

*Macvicaria ulophylla* (Steph.) S.Hatt. ≡ *Porella ulophylla*

*Macrodiplophyllum plicatum* (Lindb.) Perss. ≡ *Douinia plicata*

*Marchantia tosana* Steph. ≡ Marchantia emarginata subsp. tosana

*Marsupella commutata* (Limpr.) Bernet ≡ *Gymnomitrion commutatum*

Marsupella emarginata subsp. tubulosa (Steph.) N.Kitag. ≡ *Marsupella tubulosa*

*Marsupella parvitexta* Steph. = *Gymnomitrion commutatum*

*Marsupella revoluta* (Nees) Dumort. = *Gymnomitrion revolutum*

*Mastiogbryum bidentulum* Steph. ≡ *Bazzania bidentula*

*Mastigobryum coreanum* Steph. = *Bazzania tridens*

*Metacalypogeia quelpaertensis* S.Hatt et Inoue = *Eocalypogeia quelpaertensis*

*Metzgeria conjugata* Lindb. = *Metzgeria lindbergii*

Metzgeria conjugata subsp. japonica (S.Hatt.) Kuwah. = *Metzgeria lindbergii*

*Metzgeria decipiens* (C.Massal) Schiffn. = *Metzgeria furcata*

*Metzgeria fauriana* Steph. = *Metzgeria furcata*

*Metzgeria fruticulosa* (Dicks.) A.Evans auct. = *Metzgeria temperata*

*Metzgeria hamata* Lindenb. = *Metzgeria leptoneura*

*Metzgeria quadriseriata* A.Evans = *Metzgeria furcata*

*Microlepidozia makinoana* (Steph.) S.Hatt. ≡ *Kurzia makinoana*

*Moerckia erimona* (Steph.) S.Hatt. ≡ *Hattorianthus erimonus*

*Nardia sieboldii* (Sande Lac.) Steph. = *Nardia assamica*

*Notothylas japonica* Horik. = *Notothylas orbicularis*

*Pedinolejeunea aoshimensis* (Horik.) P.C.Chen et P.C.Wu ≡ *Cololejeunea planissima*

Pedinophyllum interruptum subsp. truncatum (Steph.) Inoue ≡ *Pedinophyllum truncatum*

Pedinophyllum interruptum var. jungermannioides (Steph.) Inoue = *Pedinophyllum truncatum*

*Pedinophyllum major-perianthium* Gao et Chang = *Pedinophyllum truncatum*

*Pellia endiviifolia* (Dicks.) Dumort. ≡ *Apopellia endiviifolia*

*Pellia fabbroniana* Raddi = *Apopellia endiviifolia*

Phaeoceros laevis subsp. carolinianus (Michx.) Prosk. = *Phaeoceros carolinianus*

*Phaeoceros miyakeanus* (Schiffn.) J.Haseg. = *Phaeoceros laevis*

*Physocolea leptolejeuneoides* Schiffn. = *Cololejeunea longifolia*

*Plagiochasma koreanum* Steph. = *Plagiochasma japonicum*

Plagiochila acanthophylla subsp. japonica (Sande Lac.) Inoue = *Plagiochila sciophila*

Plagiochila asplenioides subsp. ovalifolia (Mitt.) Inoue ≡ *Plagiochila ovalifolia*

*Plagiochila dendroides* (Nees) Lindenb. ≡ *Chiastocaulon dendroides*

Plagiochila firma Mitt. subsp. rhizophora (S.Hatt.) Inoue = *Plagiochila gracilis*

*Plagiochila japonica* Sande Lac. = *Plagiochila sciophylla*

Plagiochila ovalifolia var. miyoshiana (Steph.) S.Hatt. = *Plagiochila ovalifolia*

Plagiochila ovalifolia var. orbicularis S.Hatt. = *Plagiochila ovalifolia*

*Plagiochila quelpaertensis* Inoue = *Plagiochila ovalifolia*

*Plagiochila rhizophora* S.Hatt. = *Plagiochila gracilis*

*Plagiochila satoi* S.Hatt. = *Plagiochila porelloides*

Plagiochila semidecurrens var. grossidens Herzog = *Plagiochila semidecurrens*

*Plagiochila yokogurensis* Steph. = *Plagiochila parvifolia*

*Plagiochilion mayebarae* S.Hatt. ≡ *Chiastocaulon mayebarae*

Porella caespitans var. setigera (Steph.) S.Hatt. = Porella caespitans var. cordifolia

Porella campylophylla subsp. tosana (Steph.) S.Hatt. ≡ *Porella acutifolia*

Porella densifolia var. fallax = *Porella densifolia*

Porella oblongifolia var. takakii (S.Hatt.) Inoue = *Porella oblongifolia*

*Porella setigera* (Steph.) S.Hatt. = Porella caespitans var. cordifolia

Porella setigera var. subobtusa (Steph.) S.Hatt. ≡ *Porella subobtusa*

*Porella tosana* (Steph.) S.Hatt. ≡ Porella acutifolia subsp. tosana

Porella vernicosa subsp. fauriei (Steph.) M.Hara ≡ *Porella fauriei*

Porella vernicosa subsp. gracillima (Mitt.) Ando ≡ *Porella gracillima*

Porella vernicosa fo. spinulosa (Steph.) S.Hatt. ≡ *Porella spinulosa*

*Ptilidium sacculatum* (Mitt.) Steph ≡ *Trichocoleopsis sacculata*

*Ptychocoleus nipponicus* S.Hatt. = *Acrolejeunea pusilla*

*Riccardia pinguis* (L.) Gray = *Aneura pinguis*

Riccia glauca var. subinermis (Lindb.) Warnst. = *Riccia glauca*

*Scapania dentata* Dumort. = *Scapania undulata*

Scapania parvitexta var. minor S.Hatt. = *Scapania parvidens*

*Scapania spinosa* Steph. = *Scapania ciliata*

*Scapania stephanii* Müll.Frib. = *Scapania integerrima*

*Solenostoma cordifolium* (Dumort.) Steph. = Jungermannia exsertifolia subsp. cordifolia

*Solenostoma decurrens* (Steph.) S.Hatt. = *Solenostoma faurianum*

*Solenostoma koreanum* Steph. ≡ *Protosolenostoma fusiforme*

*Temnoma birmense* (Steph.) Horik. ≡ *Plicanthus birmensis*

*Tritomaria quinquedentata* (Huds.) H.Buch ≡ *Trilophozia quinquedentata*

*Trocholejeunea sandvicensis* Mizut. ≡ *Acrolejeunea sandvicensis*

## Discussion

The infra-regional differences inside Korea are quite significant, the richest liverwort flora being found in Jeju-do Province that houses 223 taxa. Although other provinces are less rich taxonomically, they commonly house some unique species not occurring in other administrative subunits in Korea. This is strongly evident in Gangwon Province which houses several calciphilous taxa not found in other Provinces, including *Mannia androgyna*, *M. fragrans*, *Mesoptychia ussuriensis*, *Lejeunea neelgherriana*, *Porella stephaniana* etc.

Some observations could be made in the course of comparison of Korean flora with the flora of adjacent lands, including Japan, the Russian Far East and China. The differences are most significant with the Russian Far East flora and reflect the far more northern position of the Russian Far East in comparison with the Korean Peninsula. The differences with other adjacent countries are smaller. The defined series of taxa of temperate and subtropical distribution known in Korea are absent in the Russian Far East: *Acrobolbus ciliatus*, *Acrolejeunea pusilla*, *Anastrepta orcadensis* etc. Some taxa, present in Korean peninsula, but absent in the Russian Far East, belong to the broadly Sino-Himalayan element, including *Gymnomitrion revolutum* (although the occurrence of this species in Korea is uncertain). Certain taxa, like *Mannia gracilis* and *Bazzania manczurica*, are known in all compared flora, with the exception of China. However, such taxa should likely be found in China in further studies. The differences with Japanese flora are minor and include some northern elements present in Korea, but absent in Japan, like *Biantheridion undulifolium* and *Cephalozia ambigua* (this report may be doubted), recorded in Chanbai Mt. in the northernmost North Korea or even southwards, like *Cephaloziella hampeana*, *C. varians*, *Isopaches bicrenatus* and *Scapania sphaerifera* (the latter three taxa are also not known in China). *Cephalozia lacinulata* – a rare boreal species is also known in the China mainland and Korea, but not known in Japan. *Porella chinensis* is another ‘continental’ species not known in Japan, although present in other adjacent flora. The temperate Pacific *Cavicularia densa* is not known, either in China and the Russian Far East, but is present in Korea and Japan. *Radula brunnea*, Japanese-Korean-Kuril Island-British Columbia taxon is not known in China. *Syzygiella nipponica*, a mainly Japanese species, is not known in China, although present in the Russian Far East and Korea.

Therefore, Korean hepatic flora includes several species present westwards and, for those, the Korean Peninsula is the most eastern outpost. The opposite situation occurs with some ‘Japanese’ taxa and for those, Korea is the western most outpost. Besides, some taxa, occurring northwards in the Russian Far East, penetrate the Korean Peninsula, but were not present (at least were never recorded) in Japan and China. Despite the relatively small size of the Peninsula, it houses two narrow endemic taxa of liverworts hitherto not known outside Korea. These facts show the great value of the Korean Peninsula for the conservation of natural resources and genetic potential of East Asian biota.
